# Climate signals in river flood damages emerge under sound regional disaggregation

**DOI:** 10.1038/s41467-021-22153-9

**Published:** 2021-04-09

**Authors:** Inga J. Sauer, Ronja Reese, Christian Otto, Tobias Geiger, Sven N. Willner, Benoit P. Guillod, David N. Bresch, Katja Frieler

**Affiliations:** 1 Potsdam Institute for Climate Impact Research, Potsdam, Germany; 2 Institute for Environmental Decisions, ETH Zurich, Zurich, Switzerland; 3 Deutscher Wetterdienst (DWD), Climate and Environment Consultancy, Stahnsdorf, Germany; 4 Institute for Atmospheric and Climate Science, ETH Zurich, Zurich, Switzerland; 5 Federal Office of Meteorology and Climatology MeteoSwiss, Zurich-Airport, Switzerland

**Keywords:** Attribution, Climate-change impacts, Natural hazards

## Abstract

Climate change affects precipitation patterns. Here, we investigate whether its signals are already detectable in reported river flood damages. We develop an empirical model to reconstruct observed damages and quantify the contributions of climate and socio-economic drivers to observed trends. We show that, on the level of nine world regions, trends in damages are dominated by increasing exposure and modulated by changes in vulnerability, while climate-induced trends are comparably small and mostly statistically insignificant, with the exception of South & Sub-Saharan Africa and Eastern Asia. However, when disaggregating the world regions into subregions based on river-basins with homogenous historical discharge trends, climate contributions to damages become statistically significant globally, in Asia and Latin America. In most regions, we find monotonous climate-induced damage trends but more years of observations would be needed to distinguish between the impacts of anthropogenic climate forcing and multidecadal oscillations.

## Abstract (web summary)

This study introduces an empirical modeling approach allowing to separate climate and socio-economic drivers of damages by fluvial floods. It shows that climate signals are clearly detectable in Asia and Latin America.

## Introduction

Since 1980, fluvial floods have caused more than 200,000 fatalities and more than $790 bn in direct economic damages globally^1^, placing them among the most socially and economically devastating natural disasters. Theoretical considerations on the global surface energy budget suggest that global mean precipitation increases with global mean temperature (GMT) at a rate of 1–2% per thousand of global mean warming^2^. However, the intensity of extreme precipitation events is most relevant for fluvial flooding^3^ and increases with the moisture of air that can be precipitated out according to the Clausius–Clapeyron relationship^4^. Therefore, extreme daily precipitation is expected to increase at a substantially higher rate of ~6–7% per degree of global mean warming^5^. These theoretical considerations were recently confirmed by observations showing a global median increase in annual maximum daily precipitation of 5.9–7.7% per degree of global warming^6^. In addition, there are more record-breaking rainfall events observed than would be expected in a stationary climate (12% increase in 1981–2010^7^) and the observed intensification of extreme daily precipitation events since the 1980s has been attributed to anthropogenic global warming^8^. Observed annual discharge maxima show regionally varying trends with significant increases in most stations of Asia, Europe, and Latin America and with mostly decreasing trends in Africa, Australia, and North America^9^. Globally, 1985–2009 flood frequency has first increased, peaked around 2003, and decreased afterwards^10^. Extreme flood events show a similar non-monotonous temporal evolution with strongest long-term trends in Europe and the United States of America^11^. On global and latitudinal scales, the observed variation in flood frequencies can be statistically explained by variations of four decadal and multidecadal climate oscillations: the El Niño–Southern Oscillation (ENSO), the Pacific Decadal Oscillation (PDO), the North Atlantic Oscillation (NAO), and the Atlantic Multidecadal Oscillation (AMO)^10^.

So far, trends in global flood-induced economic damages have been shown to be dominated by increasing exposure and decreasing vulnerability^12–14^, with no detectable climate-driven trend remaining after removal of socio-economic effects at the global scale as well as on the level of world regions. This finding is independent of the regions’ development level^12,15–17^ and income groups^12^. However, these studies focus on the detection of changes in vulnerability and therefore considered world regions that have been defined to be homogeneous with respect to socio-economic indicators, but not with regard to climate-induced changes in weather-related hazard indicators. This aggregation across heterogeneous trends in hazards could hide the signal of climate change.

**Fig. 1:**
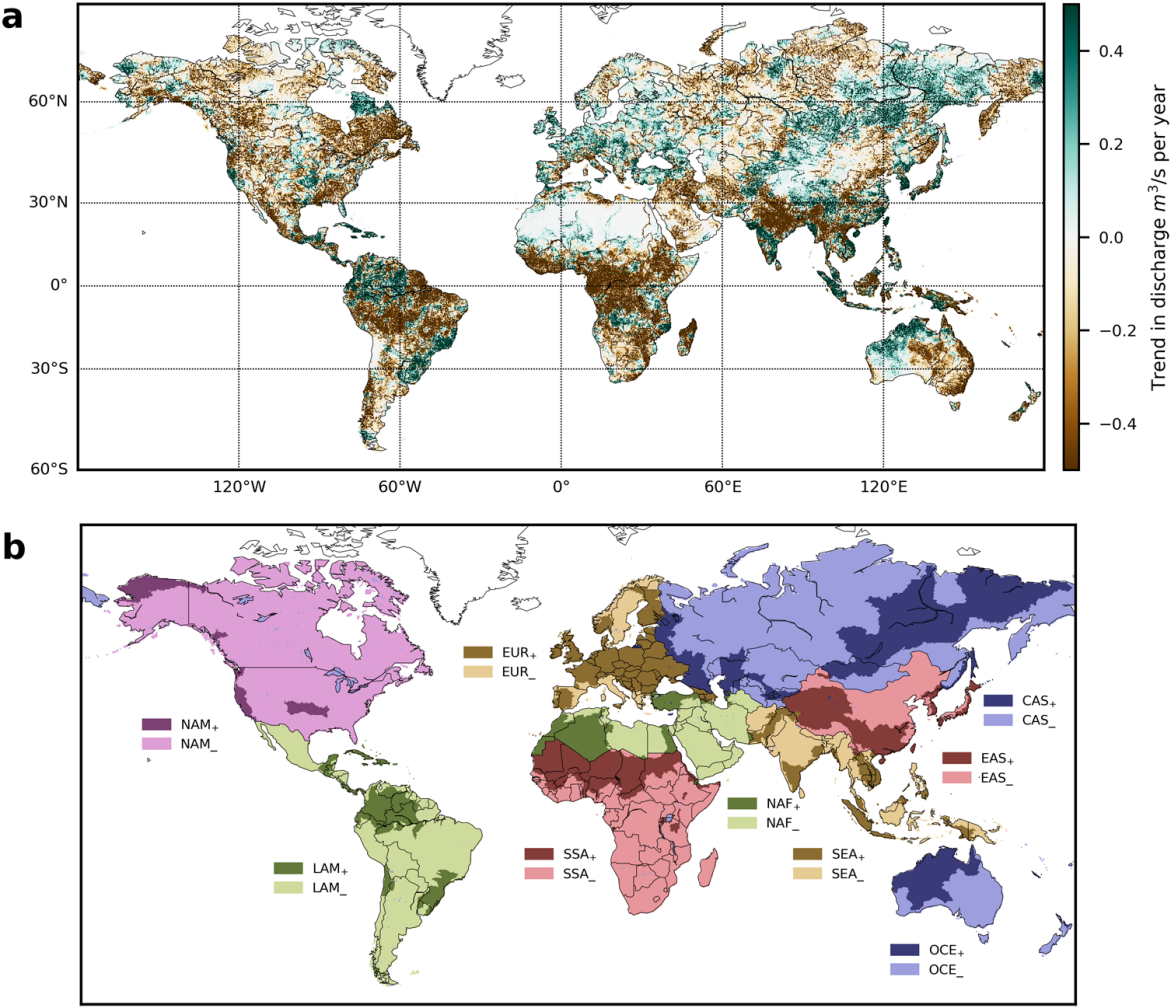
Discharge trends and definition of regions. **a** Absolute trends in annual maximum daily discharge in the time period 1971–2010 (significance levels are shown in Supplementary Fig. 3). **b** Map of the nine geographical world regions: North America (NAM), Eastern Asia (EAS), Europe (EUR), Latin America (LAM), Central Asia & Russia (CAS), South & Sub-Saharan Africa (SSA), South & South-East Asia (SEA), North Africa & Middle East (NAF), Oceania (OCE) chosen according to geographical proximity and similarity of socio-economic structure. These regions are then further divided into subregions assembled of river basins with positive (*R*_+_, dark colors) and negative discharge trends (*R*_–_, light colors) (Supplementary Fig. 4).

In this work, we investigate to what extent the observed changes in climate have already induced long-term trends in economic damages caused by fluvial flooding. To disentangle the impact of climate-induced changes in weather-related hazards (flood extent and depth) from changes in exposure of assets, and their vulnerability, we develop a hybrid process-based and empirical model. First, it overlays annual flooded areas derived from hydrological simulations forced by observational weather data^12,13^ with spatially and temporally explicit asset distributions. The exposed assets are then translated into direct economic damages by combining continental depth-damage functions^18^ with time-dependent vulnerability factors^12–14^ (Methods and Supplementary Figs. 1 and 2). Addressing the question to what degree reported time series of damages are influenced by climate change, we first verify that discharge trends modeled by the considered global hydrological models (GHMs) (Fig. 1a and Supplementary Fig. 3) compare well with observed trends^9^^19–21^, when the GHMs are driven by observed weather data (Methods and Supplementary Note 1).

We then use the modeled trends in discharge to disaggregate nine standard, socio-economically homogeneous world regions (*R*)^22^ into subregions *R*_+_ and *R*_–_ comprising the river basins with positive and negative basin-average trends in discharge, respectively (Fig. 1b, Supplementary Fig. 4, and Methods). While on the level of world regions, climate-induced damage trends are mostly averaged out, they become clearly detectable and significant in the subregions with homogeneous discharge trends in the studied historical period.

## Results

### Climate signal in flood damages

**Fig. 2:**
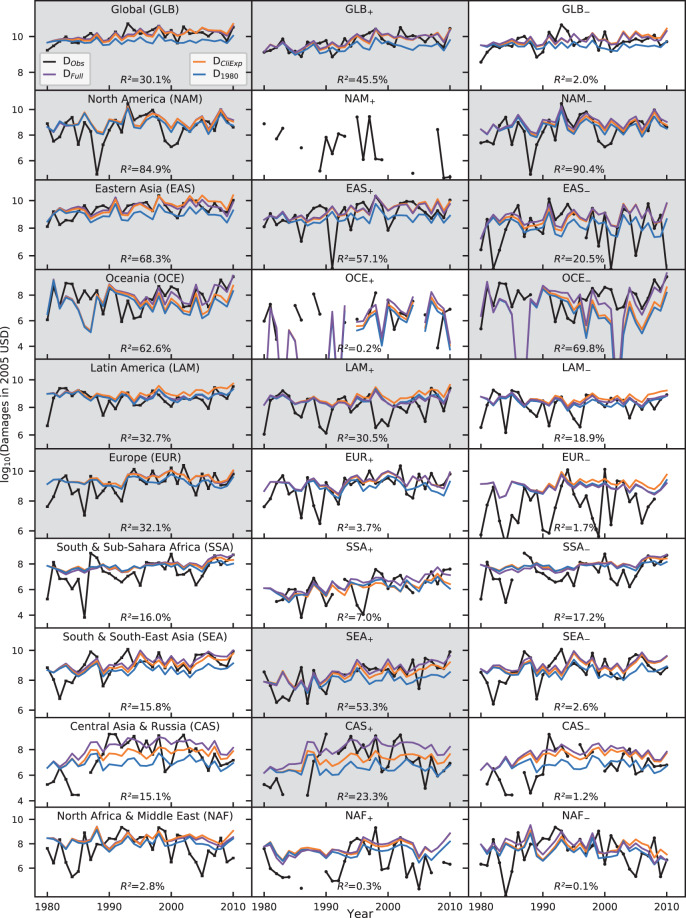
Observed and modeled time series of river flood damages (1980–2010). Time series of observed damages from the NatCatService database^1^ (*D*_Obs_, black) as well as modeled damages (multi-model median) when accounting for changes in (i) climate only (constant 1980 socio-economic conditions, *D*_1980_, blue), (ii) climate and exposure (*D*_CliExp_, orange) keeping vulnerability at 1980 conditions, and (iii) in climate, exposure, and vulnerability (*D*_Full_, purple) for the nine world regions (left panel), as well as their subregions with homogeneous positive and negative trends in river discharge (middle and right panels) (cf. Fig. 1). Time series indicating the GHM-spread are provided in Supplementary Fig. 5. Explained variances *R*^2^ are derived from the Pearson correlation coefficients between damages *D*_Full_ and *D*_Obs_. Gray background colors highlight the regions where the explained variance of the full model is higher than 20%.

When analyzing the contributions of the individual drivers (climate-induced changes in weather-related hazards, changes in exposure, and changes in the vulnerability of assets) to damage trends, we focus on regions where the full model accounting for all three drivers explains at least 20% of the variance in reported damages (in units of inflation adjusted 2005 purchasing power parities (PPP) USD) (gray panels in Fig. 2) indicating that at least parts of the critical processes determining the variability in damages are captured. In North America (NAM), the explanatory power is exceptionally high in the entire region and in the subregion with negative discharge trend ($R_{{\mathrm{NAM}}}^2$ > 80%, $R_{{\mathrm{NAM}} - }^2$ > 90%). Furthermore, high explanatory powers of more than 50% are reached in Eastern Asia (EAS) and its subregion with positive discharge trend (EAS_+_), in Oceania (OCE) and its subregion with negative discharge trend (OCE_–_), as well as in the subregion of South & South Eastern Asia with positive discharge trend (SEA_+_). Furthermore, acceptable explanatory power (*R*^2^ > 20%) is reached in Europe (EUR), Latin America (LAM), and its subregion with positive discharge trend (LAM_+_), as well as in the positive (negative) discharge subregions of Central Asia (CAS_+_) (Eastern Asia (EAS_–_)). Globally, the explanatory power of the model exceeds 30% and is slightly higher across basins with positive discharge trends ($R_{{\mathrm{GLB}} + }^2 = 45.5\%$).

To analyze how much of the variability can be explained by what driver, we additionally provide the explained variances of modeled time series accounting for (i) changes in flood hazards only and for (ii) changes in hazard and exposure (Supplementary Table 2). In most regions and subregions, accounting for climate-induced variability and trends is key for reproducing observed damages. In most cases, the explained variance only gets slightly improved by additionally considering changes in either only exposure or both exposure and vulnerability with the exception of CAS_+_, SEA_+_, OCE, and OCE_–_ where the explained variance increases strongly, and EAS_–_ and LAM_–_ where it decreases.

Climate-induced trends in damages are estimated from a restricted model accounting only for observed changes in climate while keeping exposure (in units of inflation adjusted 2005 PPP USD) and vulnerability at 1980 levels (*D*_1980_). Damage trends induced by changes in exposure are then estimated from the difference between the trend in *D*_1980_ and the trend derived from an extended model additionally accounting for changes in exposure (*D*_CliExp_). Finally, damage trends induced by changes in vulnerability are estimated from the difference in trends between *D*_CliExp_ and the full model (*D*_Full_) (Methods).

**Fig. 3:**
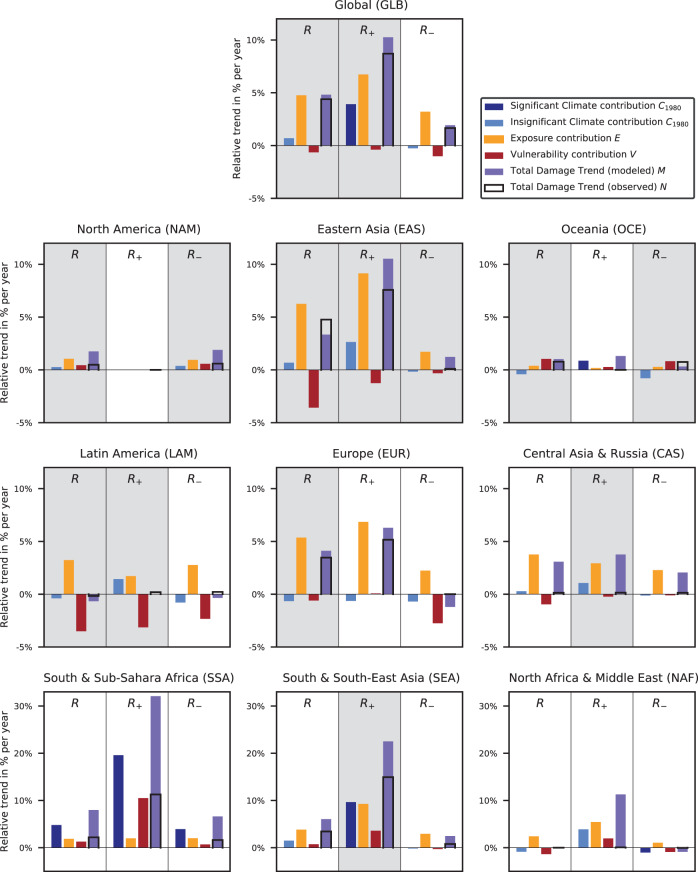
Contributions of changes in climate, exposure, and vulnerability to damages induced by river floods (1980–2010). Bars indicate the relative trend in annual modeled *M* (purple) and observed damages *N* (black frames) and the individual contributions of each driver: climate *C*_1980_ (blue), exposure *E* (orange), vulnerability *V* (red) relative to the recorded annual mean damage of the baseline period 1980–1995. From left to right, we present the trends in the entire world regions (*R*), and the subregions with positive (*R*_+_) and (negative, *R*_–_) discharge trends. Gray background colors highlight the regions where the explained variance of the full model is higher than 20%.

**Fig. 4:**
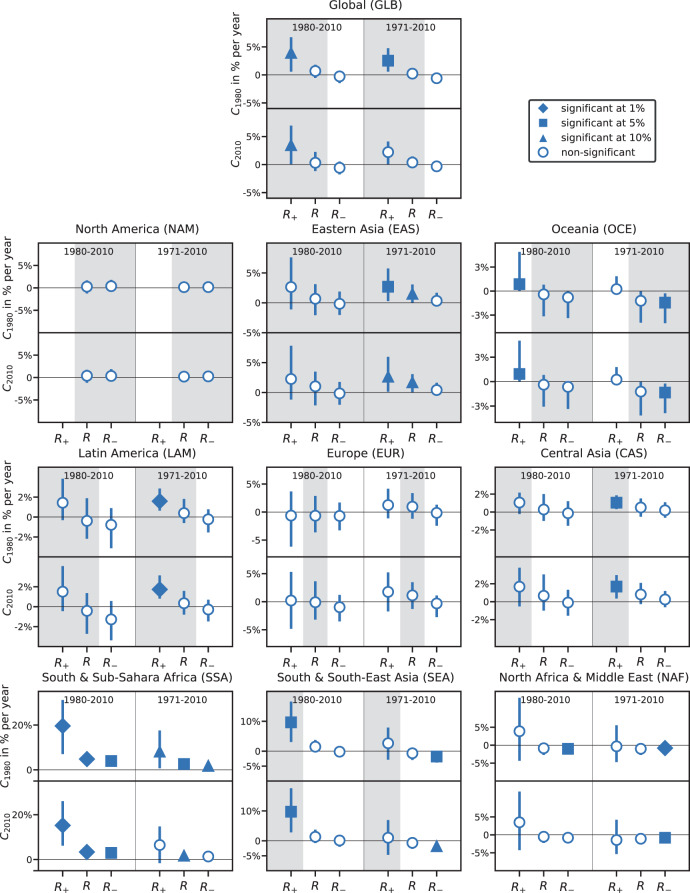
Comparison of climate-induced trends in economic damages over 1980–2010 and 1971–2010. Shown are trends for each geographical world region (*R*) as well as in the subregions with positive (*R*_+_) and negative discharge trends (*R*_–_). Error bars indicate the 90% confidence interval of the Theil–Sen slope estimation. Symbols indicate the statistical significance of the climate trends at various levels. Climate-induced trends *C* derived from simulated damages assuming fixed 1980 exposure *D*_1980_ and fixed 2010 exposure *D*_2010_ are expressed relatively to the recorded annual mean damage of the baseline period 1980–1995 in the region or subregion (*C*_1980_ and *C*_2010_). Gray background colors highlight the regions where the explained variance of the full model is higher than 20%.

In the full regions, comprising divergent trends in discharge, climate-induced trends in damages are small compared to exposure and vulnerability-induced trends and mostly insignificant except for SSA. However, when dividing the world regions into subregions with homogeneous discharge trends, climate-induced trends in damages become clearly detectable (Figs. 3 and 4) suggesting that in most regions trends in annual maximum discharge are a good proxy for climate-induced damage trends. On global level, a significant positive climate-induced trend emerges in GLB_+_ compared to the small and insignificant climate-induced global trend. In GLB_+_, as well as in SEA_+_, CAS_+_, and EAS_+_, the climate-induced trends are comparable or even larger than the trends induced by the socio-economic drivers. The same holds true for the climate-induced trends in SSA_+_, OCE_+_, and NAF_+_, where however the explanatory power of the full model is considered too low (*R*^2^ < 20%) to allow for an attribution of observed damages. In most *R*_+_ regions, climate-induced trends are positive and often significant, but mostly negative in the corresponding *R*_–_ region (Fig. 4).

However, there are some regions where trends in annual maximum discharge do not translate into similar trends in damages. For instance, we find a significant positive damage trend in SSA_-_. This can be explained by changes in the distribution of annual maximum discharge over time; the region became drier on average, but positive discharge extremes that exceed protection standards and drive economic damages intensified.

While the damage reporting by Munich Re’s NatCatSERVICE^1^ begins only in 1980, the hydrological simulations generated in the second phase of the Intersectoral Inter Model Intercomparison Project (ISIMIP2a) are available from 1971 onward. This allows for a backward extension of the simulated climate-induced damages to the period 1971–2010 and a robustness check of the climate-induced trends as obtained for the period 1980–2010. Overall, we find climate-induced trends to be robust against the choice of the measurement period (upper panels in Fig. 4): in the global positive discharge region GLB_+_, NAF_–_, and SSA and both of its subregions we find significant (at least at the 10% level) trends for both periods that agree in sign and magnitude. Good agreement between climate-induced trends is also observed in EAS_+_, OCE_–_, LAM_+_, CAS_+_, SEA_–_, where trends become significant for the longer period. Only in OCE_+_ and SEA_+_ positive climate-induced trends lose their significance from 1971 to 2010.

In addition, we test for the dependence of the climate-induced trends on the choice of the baseline year for the socio-economic forcing (Supplementary Note 2). To this end, we compare the climate-induced trends derived from *D*_1980_ to the ones derived from *D*_2010_ (cf. upper and lower panels in Fig. 4 and Supplementary Fig. 6). Differences in these two trends arise when there are assets in 2010 in areas where no assets at all existed in 1980. Assuming 2010-fixed exposure, all damages that were caused on these assets contribute to the climate-induced trend, while changes in damages on these assets in the 1980-fixed exposure are attributed to the exposure trends. However, differences between both estimates of “climate-induced damage trends” are found to be minor (Fig. 4). The calculation of the trends for fixed 2010 exposure further allows for the quantification of the contribution of climate change from 1980 to 2010 (or 1971 to 2010) to median damages in 2010 as difference between the start level of the regression line and its end level (Eq. (5) and Supplementary Table 3). This is closest to the definition of “climate impact attribution” as defined in Ch18 of the WGII contribution to the Intergovernmental Panel on Climate Change (IPCC) AR5^23^ that would require the comparison of observed damages to damages in a counterfactual situation assuming observed socio-economic conditions but a stationary climate.

### Drivers of climate-induced damage trends

**Fig. 5:**
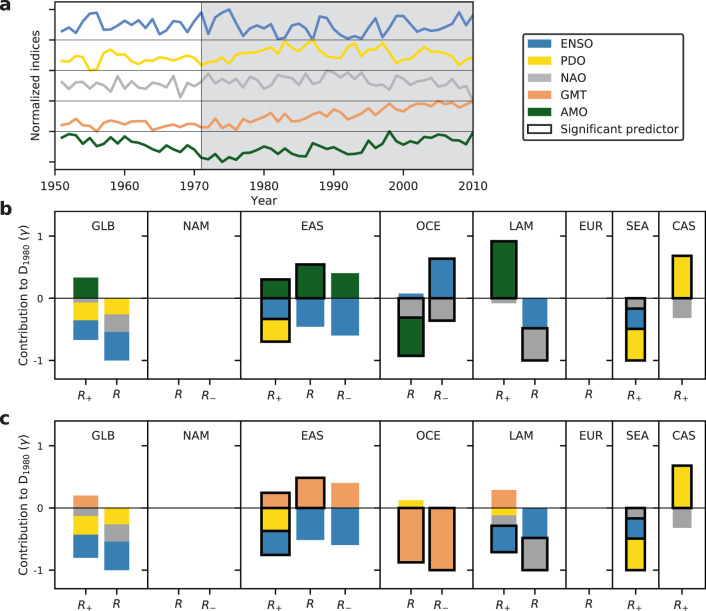
Predictors of climate-induced trends in flood damages. **a** Normalized indices of ENSO, PDO, NAO, AMO, and GMT from 1971 to 2010. Only the period 1971–2010 is used for the analysis (gray shading). **b** Relative contribution ($\gamma$) of each climate-oscillation indicator in the best GLM to the damage time series *D*_1980_ accounting for climate-induced trends and variability only (fixed 1980 exposure and vulnerability) (cf. Methods). Black boxes indicate significant predictors at 10% level. Shown are only regions with *R*^2^ > 20% for the full model. **c** Same as **b**, but using ENSO, PDO, NAO, and GMT as predictors.

To assess whether climate-induced trends in damages will persist in the future due to ongoing anthropogenic climate change or whether they are temporary and caused by climate oscillations (which would make them highly sensitive to the considered time period), we test to what degree the modeled time series of climate-induced damages for the full period 1971–2010 (*D*_1980_) can be explained by variations in ENSO, PDO, NAO, AMO, and GMT. The latter is considered a proxy for long-term anthropogenic climate change^24^. In the given time period from 1971 to 2010, the AMO index is highly correlated with GMT (Pearson correlation coefficient *r* = 0.92) and shows a similar monotonous increase (Fig. 5a) such that it can be considered a replacement of GMT in many cases. Therefore, we identify the model providing the best representation of the simulated damages while avoiding overfitting based on a leave-one-out-cross-validation (LooCV)^25^ for two separate trend analyses; in the first, the best predictors are chosen among ENSO, PDO, NAO, and GMT (Fig. 5b), and, in the second, the best predictors are chosen among all four climate oscillations (replacing GMT by AMO) (Fig. 5c) (Methods). Since all predictors are normalized, the relative shares of their coefficients show the importance of one predictor compared to the others (Fig. 4). When choosing the best model, we allow for up to 1 year lag in the response to the climate oscillations ENSO, PDO, and NAO. Among all regions where the explained variance of the full model (*D*_Full_) is higher than 20% and significant climate trends have been identified (GLB_+_, EAS, EAS_+_, OCE, OCE_–_, and LAM_+_), either GMT or AMO are predictors for the monotonous long-term trend in climate-induced damages (Fig. 5c). Only in CAS_+_, the significant climate trend can be best explained only by NAO and PDO. The signs of the coefficient of GMT or AMO are always in line with the underlying long-term trend in the damage time series, in the sense that a positive trend in observed damages is associated with a positive coefficient of GMT or AMO, respectively, and vice versa for negative trends. As the predictors have been normalized all vary from 0 to 1 in the considered period and the magnitude of the coefficients also informs about the strength of the influence of the individual predictors (Methods). In most of the above regions, the relative importance of AMO and GMT among the set of chosen predictors is similar (cf. Fig. 5b, c). However, in LAM_+_ we find AMO to be more important than GMT whereas the opposite holds true for OCE and OCE_–_.

In all regions with significant climate-induced trends, AMO or GMT are sufficient to explain the long-term trends with no significant trends remaining in the residuals of the identified most parsimonious models. The explanatory power of the best models are similar, regardless whether AMO or GMT are contained in the set of predictors (Supplementary Tables 4 and 5). Thus, the available data do not allow for a decision in favor of one of both predictors. In addition, regarding the question whether the observed climate-induced trends are expected to continue due to anthropogenic climate forcing or vanish in line with some long-term climate oscillation, even a dependence on AMO does not rule out the continuation of the observed trends as the AMO itself does not only describe the internal variability of climate but may be also affected by anthropogenic climate forcings^26–28^. Thus, while we cannot distinguish between the effects of GMT and AMO, our analysis should be considered as a test for a long-term monotonous trend even after adjusting for ENSO, PDO, and NAO.

## Discussion

In many regions, the quantification of the contribution of climate change to observed trends in flood-induced economic damages is still limited by an insufficient understanding of the observed damage time series. First of all, coarse and uncalibrated hydrological simulations such as those used here may not be able to properly reproduce actual historical flood extents linked to general limitations and uncertainties of the modeling approach (Supplementary Discussion). In addition, due to the use of multi-model medians of damage time series, modeled time series are assumed to have a relatively smaller variability than the recorded damages, explaining the differences in the significance levels between observed and modeled trends in damages. However, the excellent reproduction of observed fluctuations in damages in North America underlines the general power of the considered modeling approach. The general performance of the hydrological models is also demonstrated by the close qualitative agreement between simulated and observed trends in discharge^9,19–21^ (Supplementary Note 1). Especially for large-scale climate change impact assessments, as the trend analysis undertaken here, they have been found to be a suitable tool^29^. Qualitatively, observed and modeled damage trends match in all world regions and subregions, except for EUR_–_ and LAM_–_. Unexplained variances of observed damage data may result from regional deficits in reported damages, observational climate forcings, representation of protection standards, or asset distributions. Our analysis highlights the importance of subregional differences in impacts and the need for spatially explicit and event-specific damage records to allow for a high-regional detail in the assignment of damages. The geographical locations provided in the NatCatSERVICE database are a good starting point in this regard, but more accurate event footprints are desirable for a better regional assignment of damages.

Here, we estimate vulnerability from the ratio of observed and simulated damages as obtained when accounting for climate and exposure-driven changes in damages (*D*_CliExp_). In consequence, misrepresentation of trends in either the reported, or simulated climate and exposure-driven damages would translate into erroneous trends in vulnerability over time. For example, an underreporting of damages in early years would erroneously translate into low vulnerabilities in the early phase. Similarly, too low estimates of the climate-induced trends could be compensated by increasing vulnerability estimates. In most regions, where the explanatory power of our model is acceptable (*R*^2^ > 20%), trends in vulnerability are negative indicating that both effects may only play a limited role. However, in NAM, NAM_+_, OCE, OCE_–_, and SEA_+_, we find increasing vulnerability trends that could result from an underestimation of climate-induced trends or an underreporting of damages in the 1980s (Fig. 3 and Supplementary Figs. 1 and 2). However, increasing vulnerabilities in highly developed regions such as NAM may also be real^30^ and due to behavioral changes caused by overestimating protection, e.g., the levee effect^31^. In previous studies, mostly decreasing vulnerabilities with overall converging trends between high- and low-income countries have been found^12,14^. However, increasing vulnerability levels have also been reported for higher-middle-income countries^13^. Differences to our findings may be explained by the aggregation over countries by income and not with regard to homogeneity or discharge trends and considerations of different time periods and by the averaging method used to express the aggregated vulnerability for an entire region. Our quantification of climate change contributions to observed trends in flood-induced damages differs from “climate impacts attribution” as defined in Chapter 18 of the 5th assessment report (AR) of the IPCC AR5^23^: “Detection of impacts’ of climate change addresses the question of whether a natural or human system is changing beyond a specified baseline that characterizes its behavior in the absence of climate change.”^32^ where this “baseline may be stationary or non-stationary (e.g., due to land use change).” According to this definition, the impacts of climate change on observed trends in flood-induced damages would have to be estimated as the difference between observations and damages derived from simulation assuming stationary climate and observed changes in asset distributions and vulnerabilities. In contrast, we estimate them from a varying climate but with fixed asset distributions and vulnerabilities. However, the contributions of climate change to average damages at the end of the considered time period (2010)—estimated by multiplying climate-induced trends (1980–2010) assuming fixed 2010 socio-economic conditions with the length of this time period—basically correspond to the AR5 definition of impact attribution in 2010. As such, our approach certainly only quantifies the contribution of climate change over the 1980–2010 period and not the full contribution of climate change compared to pre-industrial levels.

It is critical to identify the individual drivers of flood-induced damages since their reduction may require different mitigation and adaptation strategies. We demonstrate that averaging across regions with heterogeneous climate-induced trends in flood hazards can hide the signal of climate change in reported time series of flood-induced damages. While previous global studies suggest that the contributions of climate to changes in flood damages have been minor compared to socio-economic drivers, we show that the impacts of climate change become detectable when disaggregating world regions into subregions with homogeneous trends in annual maximum discharge in the historical period. This works especially well for the global subregion with positive discharge trend as well as the subregions of South & South-East Asia, Eastern Asia, Central Asia & Russia, and Latin America with positive discharge trends. However, the explanatory power of the considered modeling approach is still low in these regions. In general, the considered hybrid modeling approach building upon process-based hydrological simulations and empirical estimates of vulnerabilities proves to be a powerful tool to attribute observed damages induced by river floods. While remote sensing may allow for the identification of flooded areas in recent years making use of the MODerate resolution Imaging Spectro-radiometer instruments on the NASA Aqua and Terra satellites^33^, process-based modeling remains critical, both for backward extensions required for the attribution of long-term trends and for future climate impacts projections.

Being constrained by the simulation period (1971–2010) of the ISIMIP2a hydrological model ensemble, it was not yet possible to decide whether the climate-induced change in damages is attributable to long-term warming or natural climate variability, the inclusion of the recent decade (as done in the ongoing modeling round of the ISIMIP project, ISIMIP3a; https://www.isimip.org/protocol/) may already enable us to provide a clearer answer. Nonetheless, our analysis clearly reveals an underlying monotonous climate-induced trend in damages in many regions even under adjustment for ENSO, NAO, and PDO as shorter-term climate oscillations. The generation of stationary counterfactual historical climate forcing data^34^ and their translation into flooded areas based on hydrological simulations will also allow us to apply our framework to the attribution of observed impacts as defined by the AR5^23^.

## Methods

### Climate forcings and hydrological data

For the modeling of fluvial floods, we rely on the runoff output from 12 GHMs participating in phase 2a of the Inter-Sectoral Impact Model Intercomparison Project (ISIMIP2a)^35^ (Supplementary Methods). The 12 GHMs were driven by four separate observational (atmospheric) weather data products for the period 1971–2010 providing daily runoff at 30’ (~50 km × 50 km) resolution including the Global Soil Wetness Project version 3 (GSWP3; http://hydro.iis.u-tokyo.ac.jp/GSWP3)^36^, the Princeton Global Meteorological Forcing Dataset version 2.1 (PGMFD; http://hydrology.princeton.edu/data.pgf.php)^37^, the Water and Global Change Forcing Data based on the reanalysis data set ERA-40 (WATCH; 10.1029/2006gl026047, 10.1175/jhm-d-15-0002.1)^38^, and the ERA-Interim data (WATCH-WFDEI)^39^ (Supplementary Table 1).

### Socio-economic data sources

We use gridded Gross Domestic Product (GDP) data reported in PPP in 2005 USD from the ISIMIP project^40^ with a spatial resolution of 5’ from 1971 to 2010 as a proxy for the distribution of assets. Gridded GDP data were obtained using a downscaling methodology^41^ in combination with spatially explicit population distributions from the History Database of the Global Environment (HYDE v3.2)^42,43^ and national GDP estimates^44^ at a 5’ resolution (~10 km × 10 km). Downscaled GDP data are available in 10-year increments and linearly interpolated across decades. To estimate asset values more precisely, we convert gridded GDP data into gridded capital stock (in PPP 2005 USD), using annual national data on capital stock and GDP from the Penn World Table (version 9.1, https://www.rug.nl/ggdc/productivity/pwt/). For each country the annual ratio of national GDP and capital stock was calculated and smoothed with a 10-year running mean to generate a conversion factor, which was then applied to translate exposed GDP into asset values.

Observed asset damages are taken from reported flood damages from the NatCatSERVICE^1^ database collected by Munich Re since 1980, excluding flash flood events or flooding caused by tropical cyclones. We adjusted all flood damage estimates for inflation to the reference year 2005 using country-specific consumer price indices (CPI), i.e., expressing them in the same base year as the GDP data. To do so, we constructed a conversion factor for each country based on all reported damages for a country-specific event in 2005 and the regularly CPI-adjusted values reported in Munich Re’s NatCatSERVICE database in the base year 2016. Multiplying CPI-adjusted reported flood damages by this conversion factor results in CPI-adjusted damages for 2005. In order to ensure that recorded damages are provided in the same unit as the asset data, we additionally convert recorded damages for each country *i* and each year *j* to PPP 2005 USD according to

1 $${\mathrm{D}}_{{\mathrm{Obs}}\,i,j}({USD ,CPI\,2005\,PPP})={\mathrm{D}}_{{\mathrm{Obs}}\,i,j} ({\mathrm{USD }},{\mathrm{CPI}}\,2016) \cdot \frac{{{\mathrm{D}}_{{\mathrm{Obs}}\,2005,i}({\mathrm{USD }},{\mathrm{CPI}}\,2016)}}{{{\mathrm{D}}_{{\mathrm{Obs}}\,2005,i}({\mathrm{USD }},{\mathrm{nominal}})}} \cdot p_{i,j}$$

where

2 $$p_{i,j} = \frac{{{\mathrm{GDP}}({\mathrm{real}}\,{\mathrm{PPP}}\,2005)}}{{{\mathrm{GDP}}\,({\mathrm{real}}\,2005)}}$$

denotes a country- and year-specific conversion factor. Event-specific damage estimates were then aggregated to year–country and year–region level in order to be comparable with simulated river floods for which only the annual maximum was considered. Thereby we assumed that only one flood event is observed at each grid cell during a calendar year.

### Data on climate oscillations and global mean temperature

In order to avoid interferences with long-term temperature increase, we use the pressure based Southern Oscillation Index as a predictor for ENSO (https://www.ncdc.noaa.gov/teleconnections/enso/enso-tech.php). Monthly data for AMO, NAO, and PDO were extracted from the NOAA/Climate Prediction Center (https://www.psl.noaa.gov/data/climateindices/list/). We derived annual GMT (daily mean Near-Surface Air Temperature) as the mean of three of the four input climate forcings provided in ISIMIP2a. We excluded the WATCH data set because it does not capture the full historical period.

### Flood modeling

We derive spatially explicit river discharge, flooded areas, and flood depth from the harmonized multi-model simulations of the 12 global gridded GHMs participating in ISIMIP2a (Supplementary Methods 1). We here apply the naturalized experiment referred to as “NOSOC” in the ISIMIP2a protocol, meaning that no human impacts, such as dams and water abstractions, on river flow were considered. This is legitimate for two reasons: (1) to ensure consistency with river routing simulations that do not account for human regulation of rivers, and (2) based on a previous study for some major basins that showed that the shape of the hydrograph, for peak daily flow, is not significantly different between natural and human impact experiments^45^. Furthermore, this allows us to better isolate climate-induced changes in river discharge and flood damages. For this ensemble of 46 climate data/GHM combinations (Supplementary Table 1), we follow the methodology applied previously in Willner et al.^46,47^, and first harmonize the output of the different GHMs with respect to their fluvial network using the fluvial routing model CaMa-Flood (version 3.6.2)^48^ yielding daily fluvial discharge at 15’ (~25 km × 25 km) resolution (Supplementary Methods 2). Especially for peak discharges, CaMa-Flood agrees better with observed fluvial discharges than the direct output of the hydrological models^49^. For the subsequent analysis, we then select the annual maximum daily discharge for each grid cell. For each of the 46 simulations of daily fluvial discharge and each grid cell on 15’ resolution, we fit a generalized extreme value distribution to the historical time series of the annual maximum discharge using L-moment estimators of the distribution parameters allowing for a model bias correction following the approach by Hirabayashi et al.^50^ (Supplementary Methods 2). It has been shown in several recent publications that such a hydrological modeling chain is able to reproduce patterns in observed flood impacts^12,13^. In addition to these previous studies, we account for current flood protection standards at the sub-national scale from the FLOPROS database^51^. For the final assessment, we re-aggregate the high-resolution flood depth data from 0.3’ to a 2.5’ resolution (~5 km × 5 km) by retaining the maximum flood depth as well as the flooded area fraction, defined as the fraction of all underlying high-resolution grid cells where the flood depth was larger than zero.

### Economic damage assessment

For the estimation of direct asset damages, we apply the regional residential flood depth-damage functions developed by Huizinga et al.^18^ (Supplementary Methods 3). The quantification of flood damages consists of the following steps: (1) determine exposed assets on the grid-level (2.5’ resolution) based on the flooded fraction obtained from the inundation modeling; (2) determine the grid-level damage by multiplying the exposed assets by the flood depth and the flood-depth-damage function; (3) aggregate the estimated damages spatially to the regional/subregional level, and (4) analyze the aggregated damages across different GHM simulations, assessing model medians and model spread. For steps 1–3, the open-source probabilistic natural catastrophe damage framework CLIMADA was used^52^. To account for the inhomogeneous but a priori unknown distribution of assets within a grid cell, we additionally assume that no assets are exposed to a 2-year flood event, thus subtracting the 2-year flooded fractions from the modeled flooded fraction before multiplying with the asset value. This is equivalent to assuming that nobody would construct valuable assets in regions flooded every 2 years.

In this work, we define vulnerability as the ratio of observed *D*_Obs_ to modeled damages *D*_CliExp_. Thereby, we take into account static vulnerability estimates, due to the application of continent-level depth-damage functions and the FLOPROS protection standards. In order to additionally account for dynamic vulnerability changes, we further estimate a time-dependent vulnerability behavior for each region and subregion.

The annual modeled damages for each GHM and grid cell are then aggregated to the country and regional (or subregional) level. The model median from all combinations of GHM and climate forcings then presents a time series that accounts for varying climate, exposure and, static vulnerability *D*_CliExp_. We derive the full model accounting for time variable climate, exposure, and dynamic vulnerability *D*_Full_, by including the time-dependent vulnerability function as detailed in the next paragraph. For comparability reasons, we first aggregate to nine world regions constructed by grouping countries with geographical proximity and similar socio-economic structure following the income group classification of the Worldbank^22^ (Fig. 1b). For regions and subregions, the median across all GHMs is then compared to reported damages from Munich Re’s NatCatSERVICE (Fig. 2).

### Assessing and accounting for vulnerability

To include time-varying vulnerability, we apply an approach proposed in previous vulnerability studies^12–14^. Comparing modeled and observed damages, a time trend in the ratio of recorded and modeled damages is observed that can most likely be explained by changes in socio-economic vulnerability and/or adaptive capacity. These changes are not properly reflected within the modeling chain and are, e.g., caused by the fact that the protection standards underlying the FLOPROS database are stationary in time. We apply an 11-year smoothing on the ratio of reported and modeled damages using Singular Spectrum Analysis (Figs. SI1 and SI2)^53^. Before applying the Singular Spectrum Analysis, we undertake a consistent outlier removal by excluding data points that are more than five times the 70th (*Q*_0.7_) to 30th inter-quantile range, *Q*_0.7_–*Q*_0.3_, apart from the borders of this range,

$$Q_{0.3} - 5 \cdot (Q_{0.7} - Q_{0.3}) \ < \ \frac{{{D}_{{\mathrm{Obs}}}}}{{{D}_{{\mathrm{CliExp}}}}}\ <\ Q_{0.7} + 5 \cdot \left. {(Q_{0.7} - Q_{0.3})} \right.$$

In order to achieve consistency across regions and subregions, we additionally remove data points from the set of vulnerabilities for an entire region when they are outliers with regard to the distribution in one of the subregions.

Missing yearly vulnerability values are replaced by the median vulnerability for the period 1980–2010. In entire regions, we find a low number of missing data points, with a maximum number of four missing data points in CAS. Among the subregions with sufficiently high explanatory power of the full model (*R*^2^ > 20%), we find a maximum of six missing data points in CAS_+_ and OCE_–_. In regions that were excluded from further analysis, such as NAF_+_ and OCE_+_, we see higher numbers of missing values. The resulting vulnerability functions then provide a vulnerability factor for each year that is multiplied with *D*_CliExp_ in order to derive the full model *D*_Full_. It is important to note that the applied definition of regional vulnerability as the ratio of regionally aggregated observed damages and associated simulated damages cannot be considered a spatial average of individual vulnerabilities of assets that may be subject to strong variations according to income levels^12–14^. As the aggregated simulated exposed assets may be dominated by highly valuable assets in high-income regions the vulnerability factor derived here is also expected to be strongly influenced by associated individual vulnerabilities.

### Trend estimation

Throughout this work, we use the Theil–Sen slope estimator^54^ to quantify trends and apply the non-parametric Mann–Kendall test^55^ to evaluate significance levels. In the damage analyses, trends are relative to the annual mean damage of the reference period 1980–1995 in the corresponding region or subregion. Prior to the trend estimation, we tested the time series for autocorrelation building an autocorrelation function (ACF) based on a full convolution. Low levels of autocorrelation are only detectable in residuals analyzed in the test for teleconnections, but not in damage time series. In cases where we observed autocorrelation, we additionally applied an Hamed and Rao Modified Mann–Kendall Test^56^.

### Definition of subregions by discharge trends

We subdivide the nine geographical world regions into subregions with positive and negative trends in annual discharge maxima over the period 1971–2010. To build subregions, we make use of the WMO basin and subbasin classification provided by the Global Runoff Data Centre^57^, assigning each basin to a subregion with either positive discharge trend or negative discharge trend by comparing the number of grid cells with trends of the same sign. Each subregion encompasses all river basins with predominantly positive (negative) discharge trends *R*_+_ (*R*_–_).

Studies on changes in global discharge patterns are rare and data coverage is not evenly distributed around the globe. Furthermore, the susceptibility of discharge to human intervention affects discharge records and complicates disentangling human and climatic forces in observations. We therefore derive trends in annual maximum discharge from the daily fluvial discharge at 15’ provided by the CaMa-Flood simulation described in the Methods section on Flood modeling. To resolve recorded damages in the refined subregional analysis, we make use of the event location (country, longitude, and latitude) given for each general flood event in the NatCatSERVICE data set and assign the damage to the river basin that surrounds the given event location. Damages are then aggregated across all basins that belong to the same subregion. We provide the exact number of events recorded in the NatCatSERVICE database for each region and subregion in Supplementary Table 6.

### Attributing damages to individual drivers

Given that the overall trend in damage time series is a superposition of the trends from each individual driver, we can separate the contributions from each driver for each region (subregion) *R* by calculating the trend *α* of each time series *D*_1980_, *D*_CliExp_, and *D*_Full_ and extract normalized climate-induced trends *C*, contributions from exposure *E,* and vulnerability *V* as well as trends in observed damages *N* and modeled damages *M* according to:

3 $$\begin{array}{l}C_{1980} = \frac{{\alpha _{1980}}}{n},\\ E = \frac{{\alpha _{{\mathrm{CliExp}}} - \alpha _{1980}}}{n},\\ V = \frac{{\alpha _{{\mathrm{Full}}} - \alpha _{{\mathrm{CliExp}}}}}{n}\\ N = \frac{{\alpha _{\mathrm{Obs}}}}{n},\\ M = \frac{{\alpha _{{\mathrm{Full}}}}}{n},\end{array}$$

where years in the indices denote the year of the socio-economic conditions that were kept fixed throughout the simulations (i.e., either 1980 or 2010).

We apply a non-parametric trend analysis (Theil–Sen slope estimator) to estimate *α*. Trends are given relative to the annual reported average damages in the time period 1980–1995 (*n*) in each region or subregion (Fig. 3). We additionally provide climate-induced trends from time series with 2010 fixed socio-economic conditions (Fig. 4):

4 $$C_{2010} = \frac{{\alpha _{2010}}}{n}.$$

Socio-economic trends are assessed for the period 1980–2010. As climate-induced trends are independent from observational data, we can extend it backward, making use of the full ISIMIP2a time period and additionally assess trends from 1971 to 2010. We derive the climate contribution to median damages in 2010 (Δ_2010_) compared to the start year 1971 (1980), *t*_start_’ according to

5 $${\it{\Delta }}_{2010} = C_{2010} \cdot \left( {2010 - t_{{\mathrm{start}}}} \right)$$

### Analyzing drivers for climate-induced trends in damage

Following the methodology introduced by Najibi and Devineni^10^, we apply generalized linear models (GLM) assuming damages to be log-normally distributed and assuming fixed 1980 socio-economic conditions (*D*_1980_)^10^ to assess to what degree climate-induced trends can be explained by natural climate variability and GMT. We extend the approach by Najibi and Devineni considering the four large-scale climate oscillations as predictors in a GLM by including GMT in the set of possible predictors. In this sense, our approach is similar to Armal et al.^24^. However, in contrast to Armal et al., we allow that only AMO or GMT are included as predictor in the GLM due to their strong correlation during the considered time period. In a stepwise procedure, we calculate GLMs from all possible combinations of the predictors ENSO, PDO, NAO, AMO, and GMT and a constant ε,

6 $${D}_{1980} = \beta _{{\mathrm{ENSO}}} \cdot {\mathrm{ENSO}} + \beta _{{\mathrm{PDO}}} \cdot {\mathrm{PDO}} + \beta _{{\mathrm{NAO}}} \cdot {\mathrm{NAO}} + \beta _{{\mathrm{GMT}}} \cdot {\mathrm{AMO}} + \varepsilon _1,$$

7 $${D}_{{\mathrm{1980}}} = \beta _{{\mathrm{ENSO}}} \cdot \mathrm{ENSO} + \beta _{{\mathrm{PDO}}} \cdot {\mathrm{PDO}} + \beta _{{\mathrm{NAO}}} \cdot {\mathrm{NAO}} + \beta _{{\mathrm{AMO}}} \cdot {\mathrm{GMT}} + \varepsilon _2$$

For the shorter-term oscillations ENSO, PDO, and NAO, we additionally allow for a time-lag of 1 year, in order to account for values of these predictors in one calendar year to contribute to the damages accounted for in the following calendar year.

We then select the best model applying a LooCV^25^, which allows to assess model quality outside the fitting period calculating the out-of-sample error (Supplementary Table 4). The best model is the one with the smallest out-of-sample error, we additionally test different link functions (inverse-power, identity, log). To compare the contributions of the different predictors across the different link functions, we compare the partial derivatives of the model with regard to the individual predictors (*γ*_ENSO_, *γ*_PDO_, *γ*_NAO_, *γ*_AMO_, and *γ*_GMT_) (Fig. 5). Finally, we test the residuals for remaining trends applying the non-parametric trend analysis. Previously, we applied an ACF basing on a full convolution and found very few cases with a low level of autocorrelation (GLB, GLB_+_, OCE, OCE_–_, EAS). In these regions, we additionally applied an Hamed and Rao Modified Mann–Kendall Test^56^.

## Supplementary information

Supplementary Information

Peer Review File

Description of Additional Supplementary Files

Supplementary Data 1

## Data Availability

All the data generated during this study and required to reproduce the findings are publicly available. The data on recorded damage are available from Munich Re’s NatCatSERVICE but restrictions apply to the availability of these data, which were provided by Munich Re only for the current study, and so are not publicly available. The source data for damage modeling including flooded fractions, flooded areas, and annual maximum discharge and socio-economic input data for asset generation provided within the ISIMIP framework are available at 10.5281/zenodo.4446364^58^. The shapefiles for the river basins provided by the Global Runoff Data Centre are available at https://www.bafg.de/GRDC/EN/02_srvcs/22_gslrs/223_WMO/wmo_regions_2020.html?nn=201570. The data for climate oscillations were obtained from https://www.ncdc.noaa.gov/teleconnections/enso/enso-tech.php (ENSO) and https://www.psl.noaa.gov/data/climateindices/list/ (AMO, NAO, PDO). The data supporting the results of the study and findings presented in the figures are provided in Supplementary Data 1.

## Code availability

All implementations, input, and output data used for modeling are publicly available. The detailed implementation of the flood modeling can be accessed at 10.5281/zenodo.1241051^59^.

For damage assessment, we used the natural catastrophe damage framework CLIMADA v1.5.1 available at https://github.com/CLIMADA-project/climada_python.

All supporting scripts needed to reconstruct the analysis, including a detailed description of all the data used as input and demo tutorials, are available at 10.5281/zenodo.4508783^60^.

## References

[1] Munich Re. *NatCatSERVICE Database* (Munich Reinsurance Company, Geo Risks Research, Munich) (2016).

[2] Trenberth KE (1999). Conceptual framework for changes of extremes of the hydrological cycle with climate change. Clim. Change.

[3] Ivancic T, Shaw S (2015). Examining why trends in very heavy precipitation should not be mistaken for trends in very high river discharge. Clim. Change.

[4] Boer GJ (1993). Climate change and the regulation of the surface moisture and energy budgets. Clim. Dyn..

[5] Allen MR, Ingram WJ (2002). Constraints on future changes in climate and the hydrologic cycle. Nature.

[6] Westra S, Alexander LV, Zwiers FW (2013). Global increasing trends in annual maximum daily precipitation. J. Clim..

[7] Lehmann J, Coumou D, Frieler K (2015). Increased record-breaking precipitation events under global warming. Clim. Change.

[8] Fischer EM, Knutti R (2016). Observed heavy precipitation increase confirms theory and early models. Nat. Clim. Change.

[9] Do HX, Westra S, Leonard M (2017). A global-scale investigation of trends in annual maximum streamflow. J. Hydrol..

[10] Najibi N, Devineni N (2018). Recent trends in the frequency and duration of global floods. Earth Syst. Dyn..

[11] Berghuijs WR, Aalbers EE, Larsen JR, Trancoso R, Woods RA (2017). Recent changes in extreme floods across multiple continents. Environ. Res. Lett..

[12] Jongman B (2015). Declining vulnerability to river floods and the global benefits of adaptation. Proc. Natl Acad. Sci. USA.

[13] Tanoue M, Hirabayashi Y, Ikeuchi H (2016). Global-scale river flood vulnerability in the last 50 years. Sci. Rep..

[14] Formetta G, Feyen L (2019). Empirical evidence of declining global vulnerability to climate-related hazards. Glob. Environ. Change.

[15] Barredo JI (2009). Normalised flood losses in Europe: 1970–2006. Nat. Hazards Earth Syst. Sci..

[16] Bouwer LMHave (2011). Disaster losses increased due to anthropogenic climate change?. Bull. Am. Meteorol. Soc..

[17] Paprotny D, Sebastian A, Morales-Nápoles O, Jonkman SN (2018). Trends in flood losses in Europe over the past 150 years. Nat. Commun..

[18] Huizinga, J., De Moel, H. & Szewczyk, W. Global flood depth-damage functions: Methodology and the database with guidelines. https://publications.jrc.ec.europa.eu/repository/handle/111111111/45730 (2017).

[19] Blöschl G (2019). Changing climate both increases and decreases European river floods. Nature.

[20] Gudmundsson L, Leonard M, Do HX, Westra S, Seneviratne SI (2019). Observed trends in global indicators of mean and extreme streamflow. Geophys. Res. Lett..

[21] Mediero L, Santillán D, Garrote L, Granados A (2014). Detection and attribution of trends in magnitude, frequency and timing of floods in Spain. J. Hydrol..

[22] Fantom, N. & Serajuddin, U. The World Bank’s classification of countries by income. (2016) 10.1596/1813-9450-7528.

[23] Cramer, W. et al. Detection and attribution of observed impacts. Climate Change 2014–Impacts, Adaptation and Vulnerability 979–1038 (2014) 10.1017/CBO9781107415379.023.

[24] Armal S, Devineni N, Khanbilvardi R (2018). Trends in extreme rainfall frequency in the contiguous United States: attribution to climate change and climate variability modes. J. Clim..

[25] Witten, I. H., Frank, E., Hall, M. A. & Pal, C. J. *Data Mining: Practical Machine Learning Tools and Techniques*. (Elsevier Inc., 2016). 10.1016/c2009-0-19715-5.

[26] Booth BBB, Dunstone NJ, Halloran PR, Andrews T, Bellouin N (2012). Aerosols implicated as a prime driver of twentieth-century North Atlantic climate variability. Nature.

[27] Terray, L. Evidence for multiple drivers of North Atlantic multi-decadal climate variability. *Geophys. Res. Lett*. **39**, (2012).

[28] Ting M, Kushnir Y, Li C (2014). North Atlantic Multidecadal SST Oscillation: External forcing versus internal variability. J. Mar. Syst..

[29] Hattermann FF (2017). Cross‐scale intercomparison of climate change impacts simulated by regional and global hydrological models in eleven large river basins. Clim. Change.

[30] Geiger T, Frieler K, Levermann A (2016). High-income does not protect against hurricane losses. Environ. Res. Lett..

[31] Di Baldassarre G (2015). Debates—perspectives on socio-hydrology: capturing feedbacks between physical and social processes. Water Resour. Res..

[32] Stone D (2013). The challenge to detect and attribute effects of climate change on human and natural systems. Clim. Change.

[33] Policelli, F. et al. The NASA Global Flood Mapping System. in *Remote Sensing of Hydrological Extremes* (ed. Lakshmi, V.) 47–63 (Springer International Publishing, 2017). 10.1007/978-3-319-43744-6_3.

[34] Mengel, M., Treu, S., Lange, S. & Frieler, K. ATTRICI 1.0—counterfactual climate for impact attribution. *Geosci. Model Dev. Discuss*. 1–26 (2020) 10.5194/gmd-2020-145.

[35] Gosling, S. et al. *ISIMIP2a Simulation Data from Water (global) Sector*. (GFZ Data Services, 2017). 10.5880/PIK.2017.010.

[36] Dirmeyer PA (2006). GSWP-2: multimodel analysis and implications for our perception of the land surface. Bull. Am. Meteorol. Soc..

[37] Sheffield J, Goteti G, Wood EF (2006). Development of a 50-year high-resolution global dataset of meteorological forcings for land surface modeling. J. Clim..

[38] Weedon GP (2011). Creation of the WATCH forcing data and its use to assess global and regional reference crop evaporation over land during the twentieth century. J. Hydrometeorol..

[39] Weedon GP (2014). The WFDEI meteorological forcing data set: WATCH Forcing Data methodology applied to ERA-Interim reanalysis data. Water Resour. Res..

[40] Frieler K (2017). Assessing the impacts of 1.5 °C global warming—simulation protocol of the Inter-Sectoral Impact Model Intercomparison Project (ISIMIP2b). Geosci. Model Dev..

[41] Murakami D, Yamagata Y (2019). Estimation of gridded population and GDP scenarios with spatially explicit statistical downscaling. Sustainability.

[42] Klein Goldewijk K, Beusen A, van Drecht G, de Vos M (2011). The HYDE 3.1 spatially explicit database of human-induced global land-use change over the past 12,000 years. Glob. Ecol. Biogeogr..

[43] Klein Goldewijk K, Beusen A, Doelman J, Stehfest E (2017). Anthropogenic land use estimates for the Holocene – HYDE 3.2. Earth Syst. Sci. Data.

[44] Geiger, T., Murakami, D., Frieler, K. & Yamagata, Y. Spatially-explicit Gross Cell Product (GCP) time series: past observations (1850-2000) harmonized with future projections according to the Shared Socioeconomic Pathways (2010-2100). *GFZ Data Serv*. (2017).

[45] Pokhrel Y (2012). Incorporating anthropogenic water regulation modules into a land surface model. J. Hydrometeorol..

[46] Willner SN, Levermann A, Zhao F, Frieler K (2018). Adaptation required to preserve future high-end river flood risk at present levels. Sci. Adv..

[47] Willner SN, Otto C, Levermann A (2018). Global economic response to river floods. Nat. Clim. Change.

[48] Yamazaki, D., Kanae, S., Kim, H. & Oki, T. A physically based description of floodplain inundation dynamics in a global river routing model. *Water Resour. Res*. **47**, (2011).

[49] Zhao F (2017). The critical role of the routing scheme in simulating peak river discharge in global hydrological models. Environ. Res. Lett..

[50] Hirabayashi Y (2013). Global flood risk under climate change. Nat. Clim. Change.

[51] Scussolini P (2016). FLOPROS: an evolving global database of flood protection standards. Nat. Hazards Earth Syst. Sci..

[52] Aznar-Siguan G, Bresch DN (2019). CLIMADA v1: a global weather and climate risk assessment platform. Geosci. Model Dev..

[53] Golyandina, N. & Zhigljavsky, A. *Singular Spectrum Analysis for Time Series*. (Springer-Verlag, 2013). 10.1007/978-3-642-34913-3.

[54] Sen PK (1968). Estimates of the regression coefficient based on Kendall’s Tau. J. Am. Stat. Assoc..

[55] Chandler, R. E. & Scott, E. M. *Statistical Methods for Trend Detection and Analysis in the Environmental Sciences*. doi:10.1002/9781119991571 (2011).

[56] Hamed KH, Rao AR (1998). A modified Mann-Kendall trend test for autocorrelated data. J. Hydrol..

[57] GRDC. *WMO Basins and Sub-Basins/Global Runoff Data Centre*, GRDC. 3rd rev. ext. ed. (Koblenz, Germany: Federal Institute of Hydrology (BfG)). (2020).

[58] ISIMIP. source_data_flood_attribution. Zenodo, doi:10.5281/ZENODO.4446364 (2021).

[59] Willner, S. Flood Process.(Version v1. 0. 0)., doi:10.5281/ZENODO.1241051 (2018).

[60] Sauer, I. J. et al. flood_attribution_paper v1.1 (Version v1.1). (2021) Zenodo. 10.5281/zenodo.4508783.

