# Supplementary material for: Climate signals in river flood damages emerge under sound regional disaggregation
Source: Nat Commun. 2021 Apr 9;12:2128. doi: 10.1038/s41467-021-22153-9 (PMC8035337; doi:10.1038/s41467-021-22153-9)
Supplement: Supplementary file 1 — Supplementary Information [file 41467_2021_22153_MOESM1_ESM.pdf]

Supplementary Information

Climate signals in river flood damages emerge under sound regional disaggregation

Inga J. Sauer[1,2], Ronja Reese[1], Christian Otto[1*], Tobias Geiger[1,3], Sven N. Willner[1], Benoit Guillod[2,4], David N. Bresch[2,5] , and Katja Frieler[1*]

1 Potsdam Institute for Climate Impact Research, Telegraphenberg A 56, 14473 Potsdam, Germany
2 Institute for Environmental Decisions, ETH Zurich, Universitätstr. 22, 8092 Zurich, Switzerland
3 Deutscher Wetterdienst (DWD), Climate and Environment Consultancy, Güterfelder Damm 87-91, 14532 Stahnsdorf, Germany
4 Institute for Atmospheric and Climate Science, ETH Zurich, Universitätstr. 22, 8092 Zurich, Switzerland
5 Federal Office of Meteorology and Climatology MeteoSwiss, Operation Center 1, P.O. Box 257, 8058 Zurich-Airport, Switzerland

*e-mail: christian.otto@pik.potsdam.de; katja.frieler@pik-potsdam.de

Outline

1 Supplementary Figures 2
2 Supplementary Tables 11
3 Supplementary Methods 17
4 Supplementary Notes 19
5 Supplementary Discussion 21
6 Supplementary References 23

1 Supplementary Figures

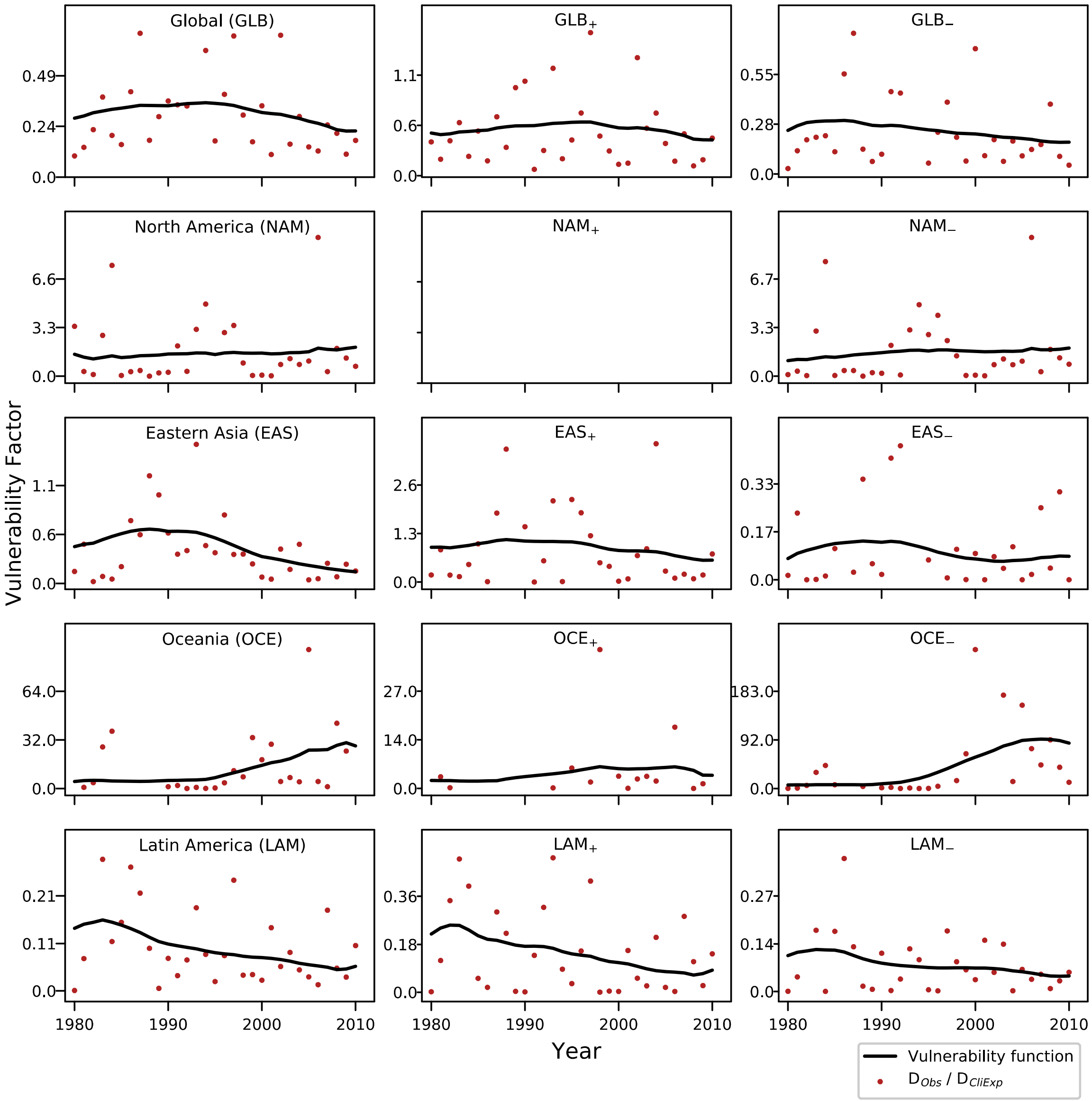

Supplementary Fig. 1: Regional time-varying vulnerability trends (R1-R5). Vulnerability estimates for the period 1980-2010 for the five best explained regions and their subregions. Data points represent the ratio of observed damages from Munich Re's NatCatSERVICE[1] database (D_{Obs}) and modeled damages accounting for time-varying exposure and climate variability and trends (D_{CliExp}).The vulnerability function was obtained applying Singular Spectrum Analysis on the annual vulnerability ratio $\frac{D_{Obs}}{D_{CliExp}}$.

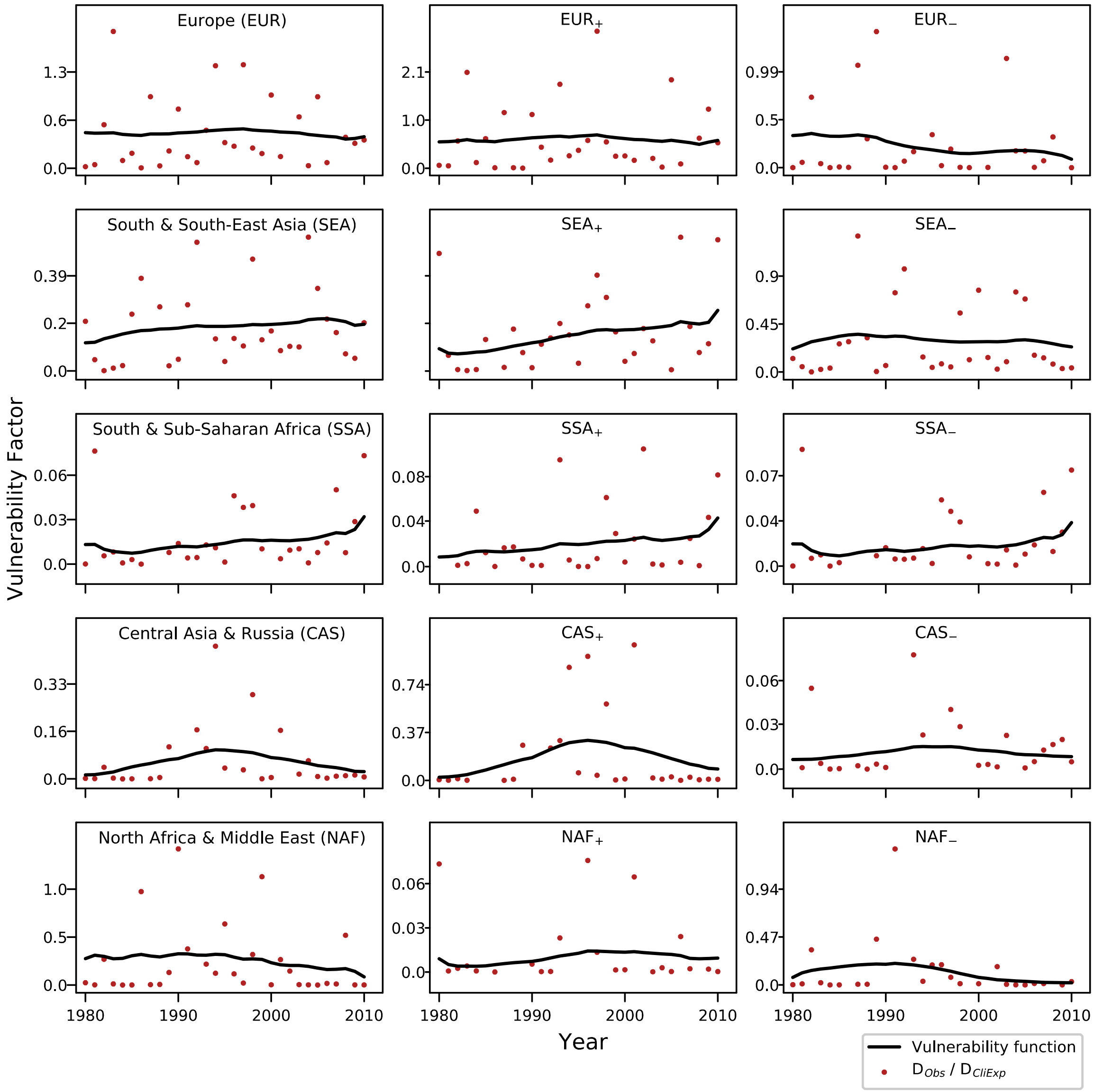

Supplementary Fig. 2: Regional time-varying vulnerability trends (R6-R10). Vulnerability estimates for the period 1980-2010 for the five main regions with low explanatory power and their subregions. Data points are the ratio of observed damages from Munich Re's NatCatSERVICE[1] database (D_{Obs}) and modeled time series accounting for time varying exposure and climate variability and trends (D_{CliExp}). The vulnerability function was obtained applying Singular Spectrum Analysis on the annual vulnerability ratio $\frac{D_{Obs}}{D_{CliExp}}$.

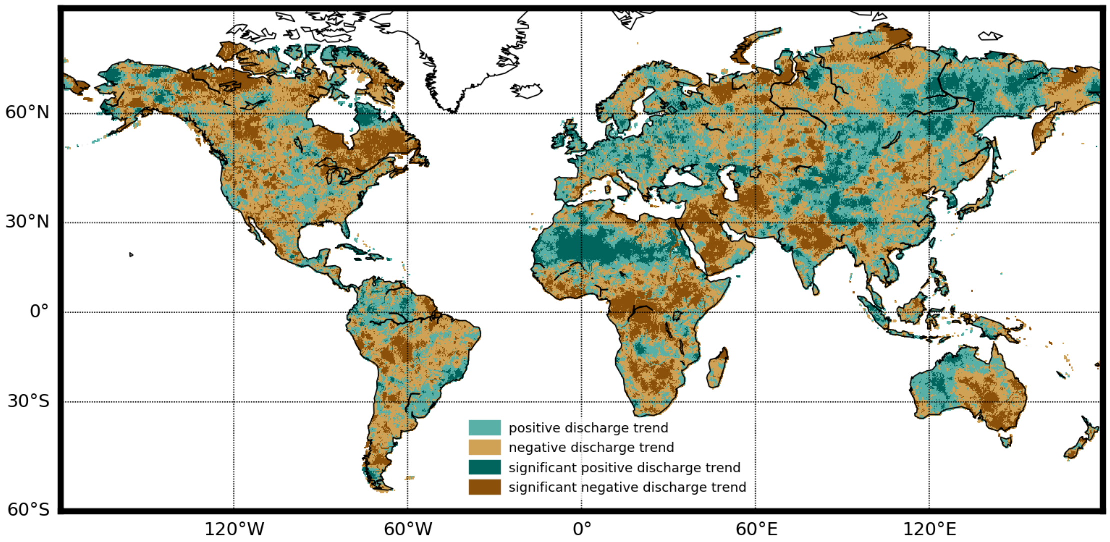

Supplementary Fig. 3: Trends in modeled annual maximum discharge 1971-2010. Global map of discharge trends derived from global hydrological models driven by observed weather data. Dark green (brown) areas indicate significant rising (declining) trends (at least at the 10% level), while the bright areas represent insignificant trends.

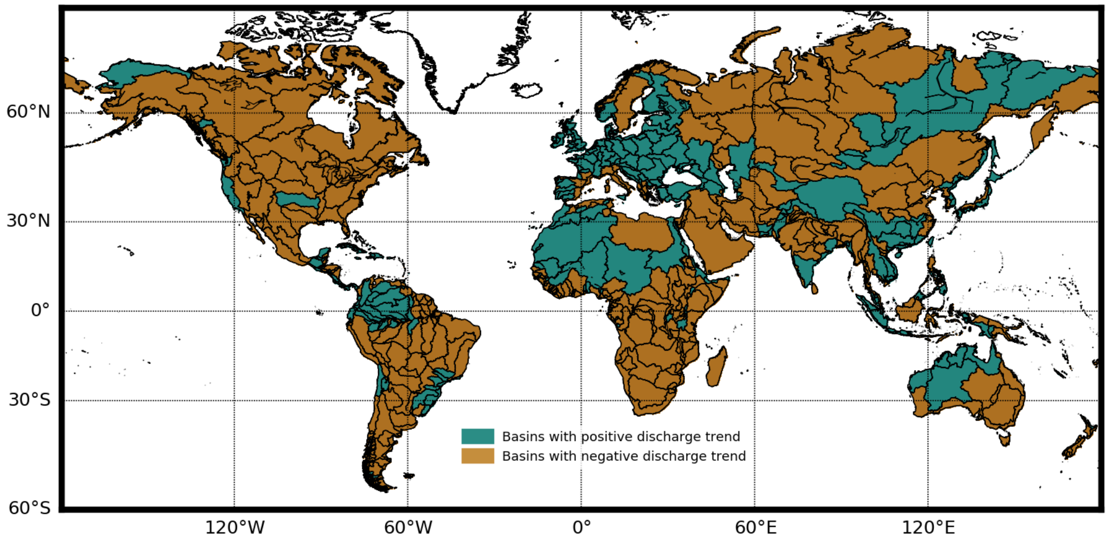

Supplementary Fig. 4: Classification of river basins according to past trends in discharge. Green areas indicate basins with predominantly positive trends in annual maximum discharge, while brown areas represent basins with mainly declining past trends in annual maximum discharge (1971-2010). Global river basins where extracted from the dataset provided by the Global Runoff Data Centre (GRDC)[2].

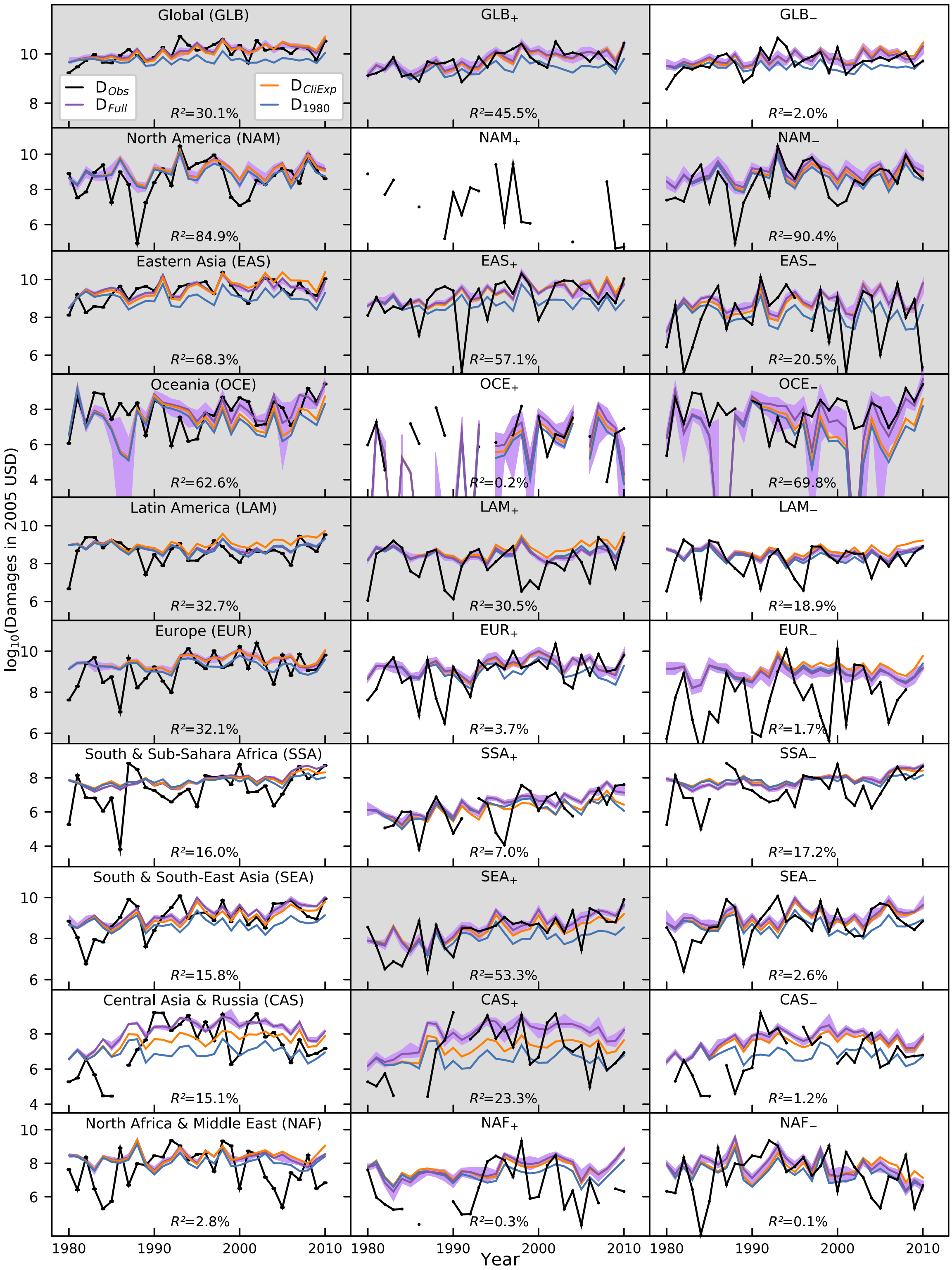

Supplementary Fig. 5: Observed and modeled damage time series with model-spread 1980-2010. Time series of observed damages (D_{Obs}, black) as well as modeled damages (multi-model median) when accounting for changes in i) climate only (constant 1980 socio-economic conditions, D_{1980}, blue), ii) climate and exposure (D_{CliExp}, orange) keeping vulnerability at 1980 conditions, and iii) in climate, exposure, and vulnerability (D_{Full}, purple) over time for the nine world regions (left main panel), as well as their subregions with homogeneous positive and negative trends in river discharge (middle and right main panels) (Fig. 1). Shaded areas mark the ⅓ and the ⅔ quantile of the multi-model output. Grey background colors highlight the regions where the explained variance of the full model R^2 is higher than 20%.

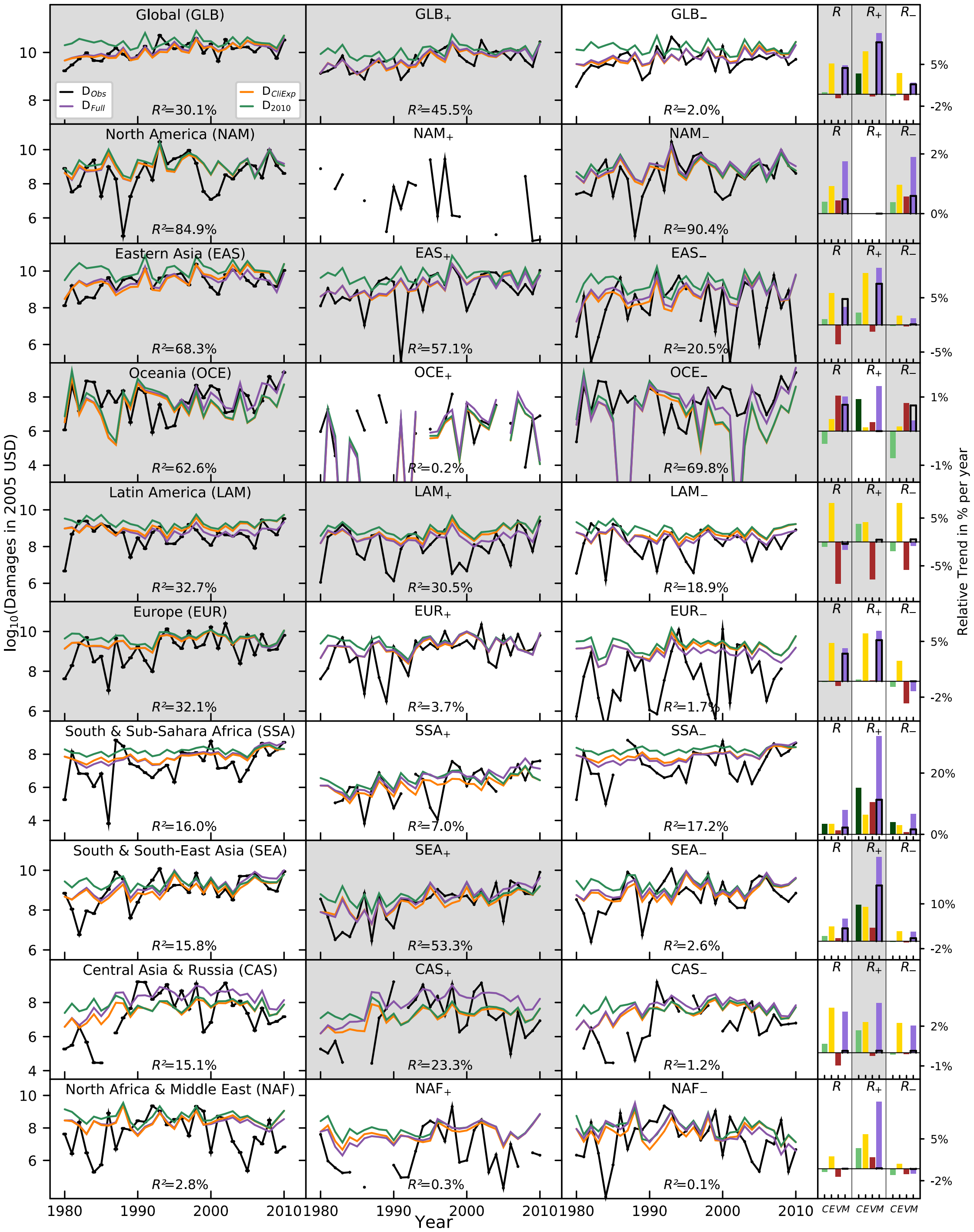

Supplementary Fig. 6: Observed and modeled damage time series (1980 – 2010) and individual contributions of each driver. Time series of observed damages as well as modeled damages (multi-model median) when accounting for changes in i) climate only (constant 2010 socio-economic conditions, D_{2010}, green), ii) climate and exposure (D_{CliExp}, orange) keeping vulnerability at 1980 conditions, and iii) in climate, exposure, and vulnerability (D_{Full}, purple) over time for the nine world regions (left main panel), as well as their subregions with homogeneous positive and negative trends in river discharge (middle and right main panels) (cf. Fig. 1). Left Bars in the side panel on the right indicate the relative trend in annual modeled (M, purple) and observed damages (N, black squares) and the individual contributions of each driver: climate variability (C_{2010}, green), exposure (E, yellow), vulnerability (V, red). R^2 indicates the explained variance of the full model compared to the observed damage and grey background colors highlight the regions where R^2>20%. Dark green bars indicate significant climate contributions and light green bars indicate insignificant contributions.

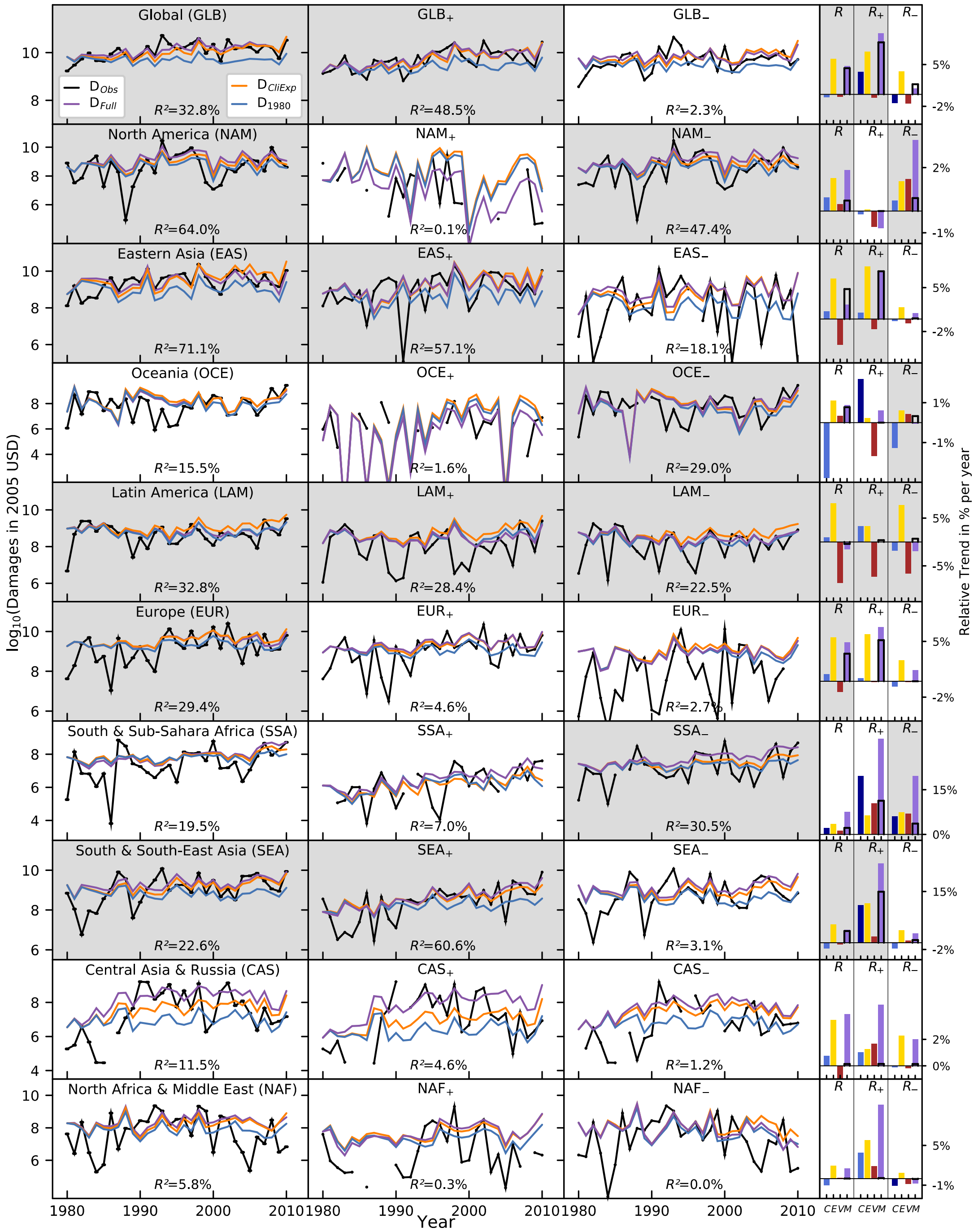

Supplementary Fig. 7: Observed and modeled damage time series for the period 1980 - 2010 and individual contributions of each driver as in Fig. 2 and 3 but using FLOPROS modeled-layer. Time series of observed damages as well as modeled damages (multi-model median) when accounting for changes in i) climate only (constant 1980 socio-economic conditions, D_{1980}, blue), ii) climate and exposure (D_{CliExp}, orange) keeping vulnerability at 1980 conditions, and iii) in climate, exposure, and vulnerability (D_{Full}, purple) over time for the nine world regions (left main panel), as well as their subregions with homogeneous positive and negative trends in river discharge (middle and right main panels) (cf. Fig. 1). Left Bars in the side panel on the right indicate the relative trend in annual modeled (M, purple) and observed damages (black squares) and the individual contributions of each driver: climate variability (C_{1980}, blue), exposure (E, yellow), vulnerability (V, red). R^2 indicates the explained variance of the full model compared to the observed damages and grey background colors highlight the regions where R^2 >20%. Dark blue bars indicate significant climate contributions and light blue bars indicate insignificant contributions.

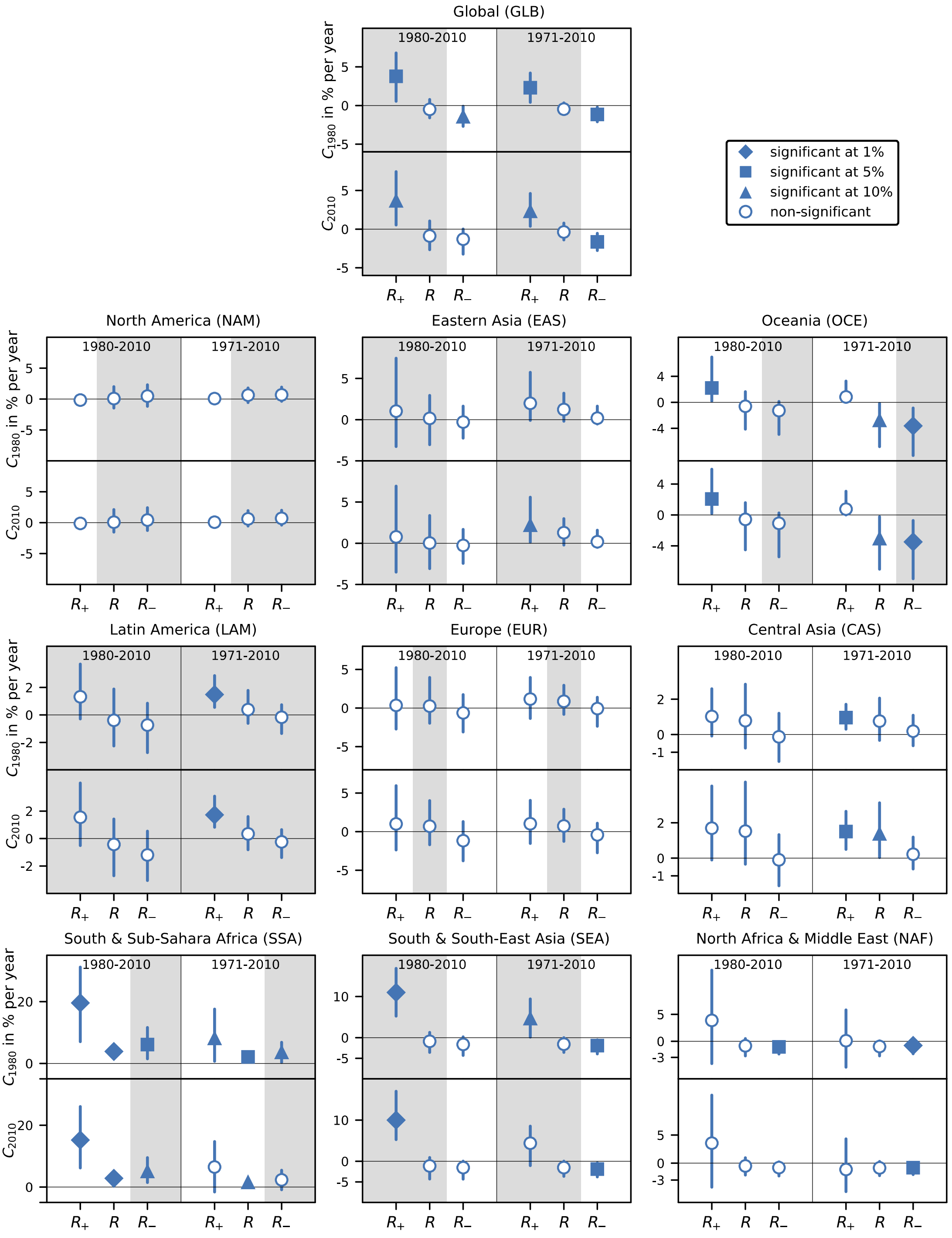

Supplementary Fig. 8: Climate-induced trends in economic damages (Simulations with FLOPROS modeled-layer). Shown are trends for each geographical world region (R) as well as in the subregions with positive R_+ and negative discharge trends R_-. Error bars indicate mark the 90% confidence interval of the Theil-Sen-slope estimation. Symbols indicate the statistical significance of the climate trends at various levels. Grey shadings indicate subregions with high explained variance (R^2 > 20%). Climate-induced trends C derived from simulated damages assuming fixed 1980 exposure (D_{1980}) and fixed 2010 exposure (D_{2010}) are expressed relatively to the recorded annual mean damage of the baseline period 1980-1995 in the region or subregion (C_{1980} and C_{2010}).

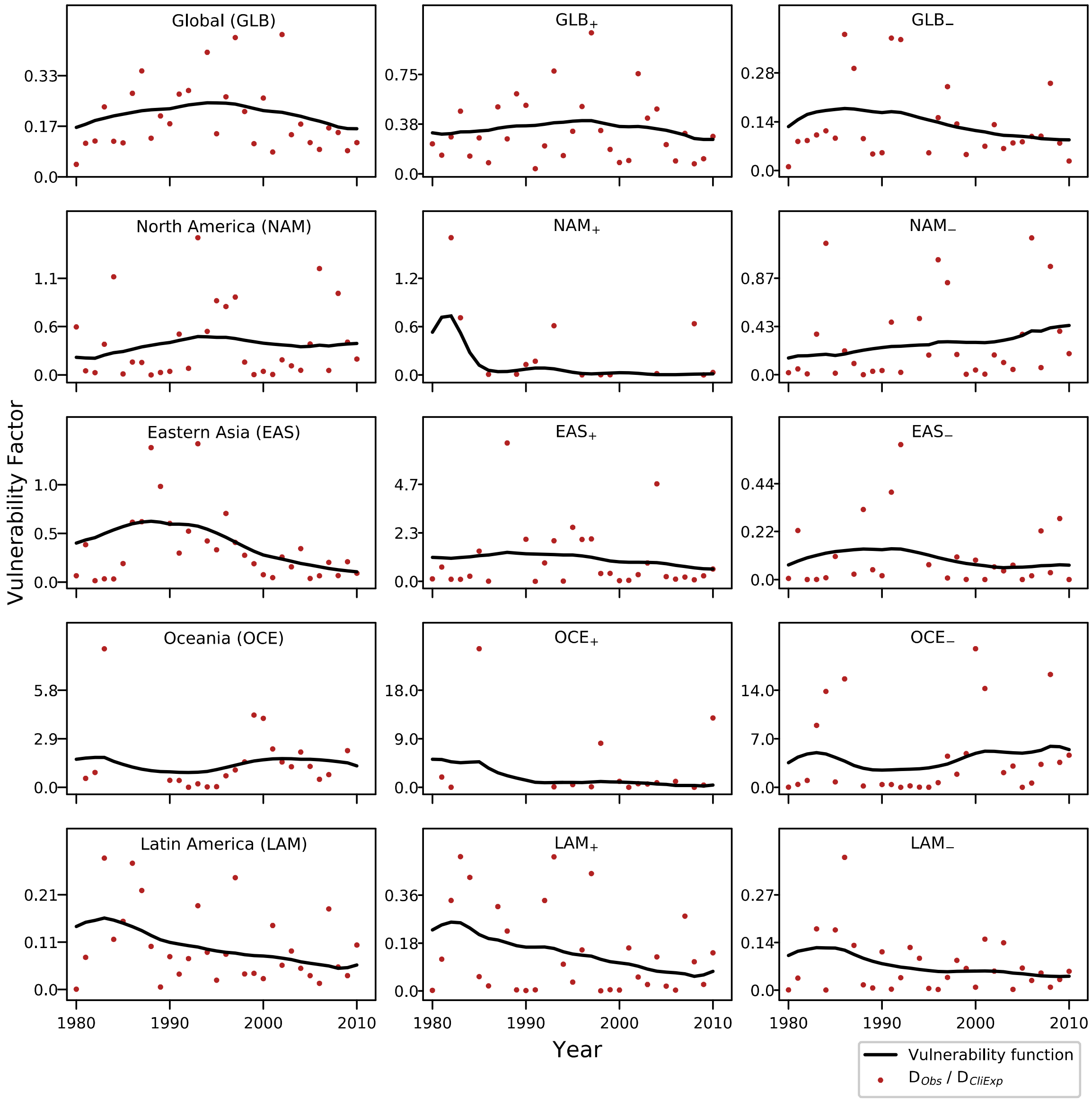

Supplementary Fig. 9: Regional time-varying vulnerability trends (R1-R5) (Simulations with FLOPROS modeled-layer). Vulnerability estimates for the period 1980-2010 for the five best explained regions and their subregions. Data points represent the ratio of observed damages from Munich Re's NatCatSERVICE[1] database (D_{Obs}) and modeled damages accounting for time-varying exposure and climate variability and trends (D_{CliExp}) .The vulnerability function was obtained applying Singular Spectrum Analysis on the annual vulnerability ratio $\frac{D_{Obs}}{D_{CliExp}}$.

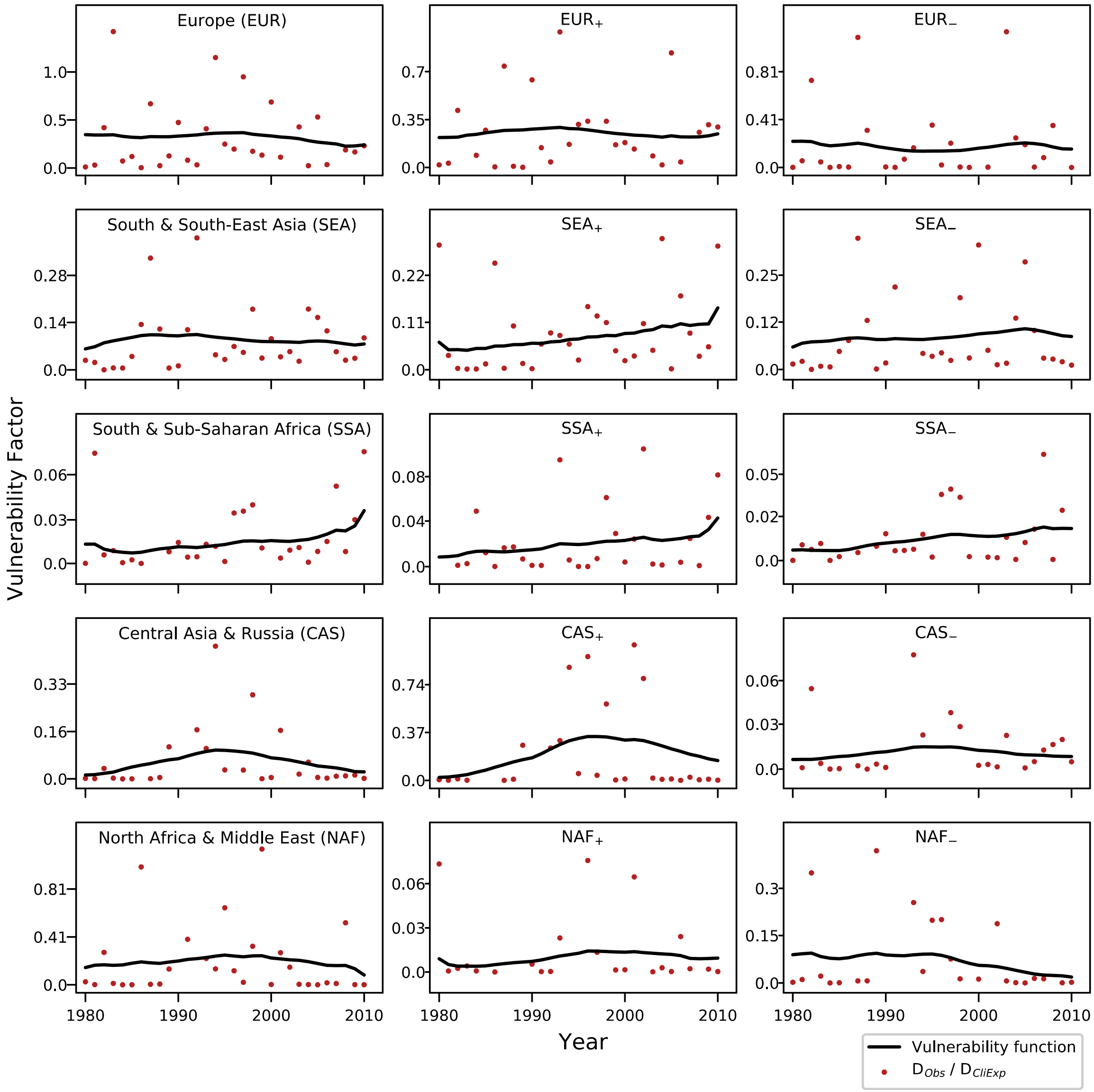

Supplementary Fig. 10: Regional time varying vulnerability trends (R6-R10) (Simulations with FLOPROS modeled-layer). Vulnerability estimates for the period 1980-2010 for the five main regions with low explanatory power and their subregions. Data points are the ratio of observed damages from Munich Re's NatCatSERVICE[1] database (D_{Obs}) and modeled time series accounting for time varying exposure and climate variability and trends (D_{CliExp}). The vulnerability function was obtained applying Singular Spectrum Analysis on the annual vulnerability ratio $\frac{D_{Obs}}{D_{CliExp}}$.

2 Supplementary Tables

Supplementary Table 1: Overview of hydrological model runs used in this study. Columns list observational weather data sets used to drive the different global hydrological models (GHMs) (rows):

	Climate forcing dataset			
**GHM**	**GSWP3**	**PGMFD**	**WATCH**	**WATCH-WFDEI**
CLM4.0	x	x	x	x
DBH	x	x	x	x
H08	x	x	x	x
JULES-W1	x	x	---	x
JULES-B1	x	x	x	---
LPJmL	x	x	x	x
MATSIRO	x	x	x	x
MPI-HM	x	x	x	x
ORCHIDEE	x	x	x	x
PCR-GLOBWB	x	x	x	x
VIC	x	x	x	x
WaterGAP2	x	x	x	x

Supplementary Table 2: Explained variances R^2 for modeled time series when considering different drivers. R^2 is derived from the Pearson-correlation coefficient of the observed damages D_{Obs} and the given modeled time series when accounting for changes in climate (D_{1980}), for changes in climate and exposure (D_{CliExp}) and for changes in climate exposure and vulnerability (D_{Full}). Grey shadings indicate subregions with high explained variance (R^2 > 20%):

Region	R^2 D_{Obs} and D_{1980}	R^2 D_{Obs} and D_{CliExp}	R^2 D_{Obs} and D_{Full}
GLB	21.0%	24.0%	30.1%
GLB_+	27.8%	43.4%	45.5%
GLB_-	6.5%	0.7%	2.0%
NAM	84.1%	84.5%	84.9%
NAM_+	---	---	---
NAM_-	90.6%	88.9%	90.4%
EAS	53.9%	45.2%	68.3%
EAS_+	47.4%	52.4%	57.1%
EAS_-	41.1%	7.1%	20.5%
AUS	2.7%	10.4%	62.6%
AUS_+	0.4%	0.2%	0.2%
AUS_-	2.5%	9.9%	69.8%
LAM	25.7%	28.7%	32.7%
LAM_+	18.7%	38.8%	30.5%
LAM_-	10.9%	5.0%	18.9%
EUR	27.5%	29.1%	32.1%
EUR_+	1.9%	3.6%	3.7%
EUR_-	2.9%	3.2%	1.7%
SSA	6.3%	12.3%	16.0%
SSA_+	4.9%	3.3%	7.0%
SSA_-	9.0%	15.0%	17.2%
SEA	8.1%	13.9%	15.8%
SEA_+	7.8%	33.4%	53.3%
SEA_-	3.4%	1.7%	2.6%
CAS	2.4%	5.2%	15.1%
CAS_+	1.0%	9.1%	23.3%
CAS_-	0.1%	0.8%	1.2%
NAF	2.4%	1.1%	2.8%
NAF_+	0.0%	0.0%	0.3%
NAF_-	0.0%	0.0%	0.1%

Supplementary Table 3: Climate-induced damage increase in 2010 relative to 1980 and 1971. C_{2010} is the annual trend in damage time series accounting only for variable climate and assuming fixed 2010 socio-economic conditions (D_{2010}) and expresses the annual additional climate contribution to damages relative to the baseline annual damage (annual mean of the period 1980-1995). We derive the climate contribution in 2010 to median damages in 2010 as difference between the start level of the regression line 1980 (1971) and its end level (2010) (Δ_{2010}), *, **, and *** denote significance at 10 %, 5%, and 1% levels, respectively. *N* in indicates the trend in observed damages (D_{Obs}) (1980-2010) normalized to the the baseline annual damage. Grey shadings indicate subregions with high explained variance (R^2> 20%):

	Time period 1980-2010			Time period 1971-2010	
**Region**	***N* in % per year**	**C_{2010} in % per year**	**Δ_{2010} in %**	**C_{2010} in % per year**	**Δ_{2010} in %**
GLB	4.39***	0.32	10.0	0.37	14.9
GLB_+	8.70***	3.46*	107.2	2.22	88.8
GLB_-	1.65*	-0.57	-17.8	-0.31	-12.5
NAM	0.48	0.39	12.2	0.21	8.33
NAM_+	---	---	---	---	---
NAM_-	0.59*	0.35	10.9	0.26	10.2
EAS	4.76***	1.02	31.7	1.7*	67.8
EAS_+	7.57**	2.26	69.9	2.64*	105.5
EAS_-	0.10	-0.15	-4.65	0.41	16.4
AUS	0.77	-0.38	-11.7	-1.22	-48.9
AUS_+	0.00	0.94**	29.0	0.24	9.68
AUS_-	0.75	-0.64	-19.9	-1.34**	-53.7
LAM	-0.16	-0.42	-12.9	0.34	13.8
LAM_+	0.19	1.51	46.7	1.73***	69.3
LAM_-	0.22	-1.28	-39.5	-0.28	-11.3
EUR	3.47**	-0.10	-2.96	1.13	45.2
EUR_+	5.16**	0.21	6.60	1.75	70.1
EUR_-	0.00	-0.98	-30.3	-0.31	-12.2
SSA	2.2**	3.35***	103.8	1.68*	67.3
SSA_+	11.3***	15.2***	471.7	6.47	258.8
SSA_-	1.62*	2.95**	91.5	1.34	53.8
SEA	3.44**	1.35	41.7	-0.67	-26.9
SEA_+	14.94***	9.75**	302.3	1.04	41.5
SEA_-	0.79	0.12	3.7	-1.81*	-72.6
CAS	0.14	0.66	20.5	0.81	32.3
CAS_+	0.15	1.67	51.9	1.69**	67.7
CAS_-	0.14**	-0.09	-2.94	0.25	10.0
NAF	0.00	-0.52	-16.2	-1.08	-43.4
NAF_+	0.08	3.5	108.5	-1.44	-57.6
NAF_-	-0.04	-0.79	-24.6	-0.81**	-32.3

Supplementary Table 4: Out-of-sample-errors for GLMs with climate oscillations (ENSO, AMO, NAO, PDO) and global mean temperature (GMT) as predictors. Best out-of-sample errors found for each combination of predictors (large-scale climate oscillations and GMT) (rows) for all well explained regions and subregions with R^2> 20% (columns):

	GLB	GLB_+	NAM	NAM_-	EAS	EAS_+	EAS_-	OCE	OCE_-	LAM	LAM_+	EUR	SEA_+	CAS_+
constant	0.0222	0.0335	0.0301	0.0290	0.0476	0.0256	0.0416	0.0299	0.0303	0.0489	0.0365	0.0469	0.0466	0.0593
ENSO	0.0197	0.0316	0.0313	0.0302	0.0457	0.0255	0.0401	0.0270	0.0274	0.0480	0.0338	0.0490	0.0466	0.0539
AMO	0.0198	0.0208	0.0304	0.0293	0.0427	0.0238	0.0406	0.0277	0.0287	0.0479	0.0123	0.0476	0.0475	0.0576
PDO	0.0227	0.0343	0.0308	0.0300	0.0490	0.0258	0.0428	0.0295	0.0297	0.0507	0.0374	0.0486	0.0431	0.0476
NAO	0.0171	0.0277	0.0308	0.0296	0.0489	0.0259	0.0430	0.0301	0.0308	0.0349	0.0266	0.0475	0.0482	0.0615
ENSO<br>AMO	0.0173	0.0190	0.0316	0.0305	0.0391	0.0231	0.0397	0.0227	0.0197	0.0462	0.0154	0.0494	0.0471	0.0548
ENSO<br>PDO	0.0188	0.0291	0.0313	0.0304	0.0447	0.0238	0.0417	0.0272	0.0276	0.0463	0.0335	0.0508	0.0313	0.0493
ENSO<br>NAO	0.0124	0.0224	0.0322	0.0310	0.0469	0.0252	0.0420	0.0193	0.0171	0.0333	0.0246	0.0488	0.0486	0.0541
AMO<br>PDO	0.0203	0.0205	0.0304	0.0299	0.0433	0.0237	0.0416	0.0278	0.0281	0.0501	0.0136	0.0498	0.0427	0.0459
AMO<br>NAO	0.0168	0.0206	0.0312	0.0299	0.0437	0.0242	0.0421	0.0251	0.0273	0.0376	0.0122	0.0489	0.0495	0.0592
PDO<br>NAO	0.0177	0.0292	0.0314	0.0303	0.0505	0.0264	0.0441	0.0291	0.0262	0.0382	0.0209	0.0492	0.0444	0.0457
ENSO<br>AMO<br>PDO	0.0161	0.0152	0.0309	0.0303	0.0400	0.0208	0.0416	0.0227	0.0198	0.0458	0.0127	0.0516	0.0313	0.0476
ENSO<br>AMO<br>NAO	0.0129	0.0175	0.0323	0.0310	0.0409	0.0237	0.0410	0.0183	0.0198	0.0364	0.0160	0.0518	0.0503	0.0555
ENSO<br>PDO<br>NAO	0.0118	0.0237	0.0321	0.0307	0.0463	0.0237	0.0434	0.0226	0.0277	0.0351	0.0156	0.0508	0.0310	0.0473
AMO<br>PDO<br>NAO	0.0174	0.0211	0.0313	0.0302	0.0455	0.0241	0.0434	0.0267	0.0263	0.0411	0.0138	0.0517	0.0447	0.0464
ENSO<br>AMO<br>PDO<br>NAO	0.0125	0.0145	0.0325	0.0313	0.0433	0.0217	0.0432	0.0230	0.0200	0.0385	0.0136	0.0540	0.0312	0.0480
GMT	0.0218	0.0268	0.0305	0.0294	0.0437	0.0239	0.0406	0.0176	0.0158	0.0498	0.0270	0.0475	0.0477	0.0604
ENSO<br>GMT	0.0191	0.0251	0.0321	0.0309	0.0392	0.0239	0.0385	0.0256	0.0255	0.0485	0.0235	0.0495	0.0475	0.0546
GMT<br>PDO	0.0223	0.0270	0.0310	0.0302	0.0442	0.0237	0.0414	0.0124	0.0277	0.0513	0.0268	0.0496	0.0440	0.0476
GMT<br>NAO	0.0174	0.0249	0.0311	0.0299	0.0456	0.0245	0.0422	0.0288	0.0282	0.0369	0.0240	0.0488	0.0494	0.0629
ENSO<br>GMT<br>PDO	0.0183	0.0220	0.0316	0.0307	0.0404	0.0223	0.0402	0.0256	0.0255	0.0465	0.0204	0.0514	0.0317	0.0496
ENSO<br>GMT<br>NAO	0.0131	0.0198	0.0332	0.0317	0.0421	0.0247	0.0403	0.0258	0.0255	0.0355	0.0095	0.0514	0.0498	0.0544
GMT<br>PDO<br>NAO	0.0180	0.0257	0.0317	0.0307	0.0468	0.0243	0.0435	0.0274	0.0280	0.0404	0.0123	0.0512	0.0462	0.0468
ENSO<br>GMT<br>PDO<br>NAO	0.0128	0.0183	0.0324	0.0316	0.0440	0.0232	0.0424	0.0256	0.0239	0.0376	0.0090	0.0538	0.0315	0.0486

Supplementary Table 5: Explanatory power of best models with climate oscillations and GMT as predictors. We show the explanatory power of the best model from simulations with the predictors NAO, PDO, ENSO, and AMO (R^2_{AMO}) and with the predictors NAO, PDO, ENSO and GMT (R^2_{GMT}) as well as the residual trends and p-values derived from the Mann-Kendall-Test ($\mathrm{p\text{-}val_{AMO}}$ and $\mathrm{p\text{-}val_{GMT}}$). Grey shadings indicate subregions with high explained variance (R^2> 20%):

Region	Significant climate trend in D_{1980}	R^2_{GMT} in %	residual trend (GMT)	residual trend $\mathrm{p\text{-}val_{GMT}}$	R^2_{AMO} in %	residual trend (AMO)	residual trend $\mathrm{p\text{-}val_{AMO}}$
GLB	no	59.5	decreasing	0.718	59.5	decreasing	0.718
GLB_+	yes (increasing)	59.4	decreasing	0.357	65.7	decreasing	0.616
GLB_-	no	33.1	increasing	0.584	27.7	decreasing	0.100
NAM	no	0.00	increasing	0.825	0.00	increasing	0.825
NAM_+	---	---	---	---	---	---	---
NAM_-	no	0.00	increasing	0.753	0.00	increasing	0.753
EAS	yes (increasing)	29.7	decreasing	0.334	32.8	decreasing	0.753
EAS_+	yes (increasing)	61.6	decreasing	0.300	78.7	decreasing	0.718
EAS_-	no	13.4	decreasing	0.492	10.4	decreasing	0.972
AUS	no	73.9	increasing	0.004	87.0	increasing	0.105
AUS_+	no (increasing 1980-2010)	7.0	increasing	0.311	7.0	increasing	0.311
AUS_-	yes (decreasing)	65.5	increasing	0.031	74.4	decreasing	0.159
LAM	no	41.5	increasing	0.916	41.5	increasing	0.916
LAM_+	yes (increasing)	83.6	decreasing	0.735	75.5	decreasing	0.166
LAM_-	no	15.8	decreasing	0.616	15.8	decreasing	0.616
EUR	no	0.00	increasing	0.449	0.00	increasing	0.449
EUR_+	no	0.00	increasing	0.370	0.00	increasing	0.370
EUR_-	no	28.8	increasing	0.370	31.4	increasing	0.718
SSA	yes (increasing)	41.3	increasing	0.370	40.1	increasing	0.825
SSA_+	yes (increasing)	32.6	increasing	0.753	35.8	increasing	0.735
SSA_-	yes (increasing)	37.5	increasing	0.584	41.9	increasing	0.718
SEA	no	27.8	decreasing	1.000	25.7	decreasing	0.188
SEA_+	No (increasing 1980-2010)	37.2	increasing	0.568	37.2	increasing	0.568
SEA_-	no	22.1	decreasing	0.421	4.2	decreasing	0.011
CAS	no	29.8	decreasing	0.972	32.6	decreasing	0.718
CAS_+	yes (increasing)	29.8	increasing	0.600	29.8	increasing	0.600
CAS_-	no	24.2	increasing	0.898	32.3	decreasing	0.735
NAF	no	4.5	increasing	0.139	7.8	decreasing	0.807
NAF_+	no	41.0	decreasing	0.568	41.0	decreasing	0.568
NAF_-	yes (decreasing)	11.5	increasing	0.173	8.67	increasing	0.239

Supplementary Table 6: Summary statistics. The table provides an over view over the number of events, total observed damage, annual mean damage and standard deviation for each region and subregion given in NatCatSERVICE[1] corrected for inflation and converted to USD PPP 2005. The mean vulnerability can be understood as an calibration factor and describes to which degree the uncalibrated model output D_{CliExp} over- or underestimates recorded damages D_{Obs}:

Region	Number events	Tot. observed damage in USD PPP 2005	Annual mean damage in USD PPP 2005	Std. Dev. In USD PPP 2005	Mean vulnerability
GLB	3988	$4.70 \cdot 10^{11}$	$1.52 \cdot 10^{10}$	$1.17 \cdot 10^{10}$	0.29
GLB$_+$	1794	$2.40 \cdot 10^{11}$	$7.75 \cdot 10^{9}$	$7.96 \cdot 10^{9}$	0.48
GLB$_-$	2183	$2.30 \cdot 10^{11}$	$7.43 \cdot 10^{9}$	$8.57 \cdot 10^{9}$	0.23
NAM	272	$7.05 \cdot 10^{10}$	$2.28 \cdot 10^{9}$	$5.44 \cdot 10^{9}$	1.67
NAM$_+$	35	$7.04 \cdot 10^{9}$	$2.27 \cdot 10^{8}$	$6.68 \cdot 10^{8}$	--
NAM$_-$	237	$6.35 \cdot 10^{10}$	$2.05 \cdot 10^{9}$	$5.34 \cdot 10^{9}$	1.66
EAS	296	$1.41 \cdot 10^{11}$	$4.55 \cdot 10^{9}$	$4.94 \cdot 10^{9}$	0.38
EAS$_+$	201	$9.90 \cdot 10^{10}$	$3.19 \cdot 10^{9}$	$4.37 \cdot 10^{9}$	0.87
EAS$_-$	95	$4.21 \cdot 10^{10}$	$1.36 \cdot 10^{9}$	$2.68 \cdot 10^{9}$	0.11
AUS	197	$9.60 \cdot 10^{9}$	$3.10 \cdot 10^{8}$	$5.58 \cdot 10^{8}$	15.0
AUS$_+$	34	$4.11 \cdot 10^{8}$	$1.32 \cdot 10^{7}$	$3.42 \cdot 10^{7}$	5.73
AUS$_-$	158	$9.19 \cdot 10^{9}$	$2.97 \cdot 10^{8}$	$5.57 \cdot 10^{8}$	42.0
LAM	601	$2.53 \cdot 10^{10}$	$8.18 \cdot 10^{8}$	$8.69 \cdot 10^{8}$	0.10
LAM$_+$	286	$1.32 \cdot 10^{10}$	$4.27 \cdot 10^{8}$	$6.46 \cdot 10^{8}$	0.15
LAM$_-$	313	$1.21 \cdot 10^{10}$	$3.91 \cdot 10^{8}$	$4.71 \cdot 10^{8}$	0.07
EUR	704	$1.21 \cdot 10^{11}$	$3.90 \cdot 10^{9}$	$5.64 \cdot 10^{9}$	0.45
EUR$_+$	561	$8.66 \cdot 10^{10}$	$2.79 \cdot 10^{9}$	$4.45 \cdot 10^{9}$	0.64
EUR$_-$	141	$3.43 \cdot 10^{10}$	$1.11 \cdot 10^{9}$	$3.16 \cdot 10^{9}$	0.23
SSA	558	$3.66 \cdot 10^{9}$	$1.18 \cdot 10^{8}$	$1.92 \cdot 10^{8}$	0.02
SSA$_+$	121	$2.05 \cdot 10^{8}$	$6.61 \cdot 10^{6}$	$1.15 \cdot 10^{7}$	0.02
SSA$_-$	436	$3.46 \cdot 10^{9}$	$1.12 \cdot 10^{8}$	$1.88 \cdot 10^{8}$	0.02
SEA	983	$8.01 \cdot 10^{10}$	$2.58 \cdot 10^{9}$	$3.11 \cdot 10^{9}$	0.17
SEA$_+$	408	$2.43 \cdot 10^{10}$	$7.83 \cdot 10^{8}$	$1.51 \cdot 10^{9}$	0.15
SEA$_-$	574	$5.58 \cdot 10^{10}$	$1.80 \cdot 10^{9}$	$2.77 \cdot 10^{9}$	0.27
CAS	154	$8.80 \cdot 10^{9}$	$2.84 \cdot 10^{8}$	$5.12 \cdot 10^{8}$	0.06
CAS$_+$	78	$6.36 \cdot 10^{9}$	$2.05 \cdot 10^{8}$	$4.51 \cdot 10^{8}$	0.18
CAS$_-$	76	$2.43 \cdot 10^{9}$	$7.85 \cdot 10^{7}$	$2.79 \cdot 10^{8}$	0.02
NAF	223	$1.06 \cdot 10^{10}$	$3.41 \cdot 10^{8}$	$5.83 \cdot 10^{8}$	0.23
NAF$_+$	70	$3.09 \cdot 10^{9}$	$9.97 \cdot 10^{7}$	$3.74 \cdot 10^{8}$	0.01
NAF$_-$	153	$7.47 \cdot 10^{9}$	$2.41 \cdot 10^{8}$	$4.82 \cdot 10^{8}$	0.13

*in some regions the number of events in the subregions does not perfectly equal the number in the entire region, this occurs due to zero modeled discharge in some insular regions.

3 Supplementary Methods

1 ISIMIP2a Flood modeling

ISIMIP2a includes four historical (atmospheric) climate data sets at 30arcmin resolution: the Global Soil Wetness Project version 3 (GSWP3; http://hydro.iis.u-tokyo.ac.jp/GSWP3)[3], the Princeton Global Meteorological Forcing Dataset version 2.1 (PGMFD; http://hydrology.princeton.edu/data.pgf.php)[4], the Water and Global Change Forcing Data based on the reanalysis data set ERA-40 (WATCH; http://dx.doi.org/10.1029/2006gl026047, http://dx.doi.org/10.1175/jhm-d-15-0002.1)[5], and the ERA-Interim data (WATCH-WFDEI)[6]. These serve as input for 12 global grid-based hydrological models (GHMs) providing daily runoff at a 30arcmin resolution: CLM4[7], DBH[8], H08[9, 10, 11], JULES-B1[12] (only for GSWP3, PGMFD, WATCH), JULES-W1[13, 14](only for GSWP3, PGMFD, WATCH-WFDEI), LPJmL [15, 16, 17, 18, 19], MATSIRO[20, 21]; MPI-HM[22], ORCHIDEE[23, 24], PCR-GLOBWB[25, 26, 27, 28, 29] , VIC (http://dx.doi.org/10.1029/2006gl026047, http://dx.doi.org/10.1175/jhm-d-15-0002.1)[30], and WaterGAP2 (http://dx.doi.org/10.5194/hess-18-3511-2014)[31].

2 Return period assessment and flood inundation mapping

For the subsequent analysis, we select the annual maximum daily discharge for each grid cell. For each simulation for the available historic period (1971–2010) and each grid cell, we fit the generalized extreme value (GEV) distribution[32] to the historical time series of the annual maximum discharge using L-moment estimators[33] of the distribution parameters as discussed in Willner et al. (2018)[34].

For model bias correction, we follow the approach by Hirabayashi et al. (2013)[35]. We map the return period of each event to the corresponding flood depth in a MATSIRO[21] model run driven by observed climate forcing[36], in bins of 1-year (1 to 100) and 10-year (100 to 1000) return periods (linearly interpolated), providing flood depth at 15arcmin resolution. Results from this observation-driven MATSIRO output have been shown to have realistic consistency in comparison with observation-based data. For this mapping, we further respect a threshold given as current flood protection at the subnational scale. This has recently been compiled in a global database (FLOPROS database) representing the currently best global-scale knowledge in the maximum return period of flood that each country/region can prevent[37]. Here, we use the "Merged layer" of this database, which combines empirical data about existing protection infrastructure ("Design layer"), data about protection standards and requirements set by policy measures ("Policy layer"), and model output from an observed relationship between gross domestic product per capita and flood protection ("Model layer"). This threshold procedure implies that, when the protection level is exceeded, the flood happens as if there was no protection in the first place (for example, dams break); below the threshold no flooding takes place.

For the final assessment, we downscale the resulting flood depth to a 18arcsec resolution using high resolution flood difference maps and re-aggregate to a 2.5arcmin resolution retaining the maximum flood depth as well as the flooded area fraction, defined as the fraction of all underlying high resolution grid cells where the flood depth was larger than zero.

3 Additional information on damage functions

For the purpose of our analysis we assume that the residential damage function is representative of all other damage categories. This is motivated by the fact that (1) residential damages regularly make up the largest fraction of flood damages, (2) the variation in the damage functions for different categories is small, compared to the uncertainty of the regional distribution of specific asset classes on global level, (3) we are presently unable to differentiate the different categories in the socio-economic data within a grid cell or distribute the different categories across grid cells (except for agriculture) as globally consistent data other than GDP is not available at present.

4 Supplementary Notes

1 Comparison of observed and modeled global discharge

Comparing the results of modeled discharge trends (Fig. 1a, Supplementary Fig. 3) to studies on regional and global discharge patterns[38, 39, 40, 41], we see a good correspondence between observed and modeled trends. In contrast to extreme precipitation, there are globally more areas with declining trends than with increasing trends for the period 1971-2010, this result is in line with empirical studies for the period 1966-2005[39]. For this period, there were more stations showing decreasing trends in North America, Australia and Africa and more stations with increasing trends in Europe, Asia and Latin America. Similarly, the modeled discharge map shows greater shares of decreasing discharge trends in North America, Africa and Australia and rather increasing trends in Europe and Asia, while in Latin America there are also slightly more areas with decreasing trends (Fig. 1a). Considering entire world regions, differences are very likely to occur as gauge stations are very unevenly distributed, while modeled results are provided on a regular 0.25° degree global grid. In Europe, the decreasing tendencies in the Mediterranean found by Blöschl et al. (2019)[38] and Gudmundssson et al. (2019)[40] are also reflected in modeled results. While Blöschl et al. find decreases in Eastern Europe, modeled results show rather increasing trends in this area, however, small increasing trends modeled in Central and Northern Europe were also identified by Gudmundssson et al. (2019)[40], all studies show the slightly decreasing trends in some areas of Scandinavia.
For North America observed patterns are similar to modeled ones, with slightly increasing trends in Central Northern America (CNA) in annual maximum streamflows and rather declining trends in the coastal regions, however increasing trends in CNA are mostly insignificant in both models and observations. In Eastern North America (ENA) a significant declining tendency is visible in observations, while in modeled results we also see declining trends which are mostly not significant.

In Latin America observed maximum discharge increases in the Amazonian region and in South-eastern South America, while significant decreases are observed in North-Eastern Brazil (cf. Fig. 1a with Fig. 2 in[40]). While a significant decline in maximum stream flows was observed in South Asia[40], the discharge trend map also shows clearly increasing trends in some areas of India and neighbouring states, however, they are mostly insignificant. In Eastern Asia, observations show significant increases in the time period 1961–2000, which are also present in modeled maximum stream flows, when considering the period 1971-2010. In Southern Australia significant and strong decreases were observed over the same time period, these are especially reflected in South Eastern Australia in the modeled map. In areas where we identify increasing trends there seem to be very few gauge stations available. In their global analysis of peak discharges, Do et al. (2017) do not find strong effects of neither presence of dams nor changes in forest cover[39]. Considering that modeled discharge does not take into account any changes in human river engineering or urbanisation patterns, the overall agreement of signs in the trend analysis also points towards only limited effects of river engineering and urbanisation in this large-scale analysis. The differences in Eastern Europe and India could be caused by human interventions, however, also limitation in hydrological modeling or availability or data quality of observations can explain these differences.

2 Sensitivity of climate-induced trends in damages to the selection of the baseline exposure

In this work, we aim to quantify the contribution of climate to changes in flood damages in the periods 1980-2010 and 1971 -2010. This exercise is known as “impact attribution”. The detection of climate change impacts implies an assessment of the changes in a natural or human system compared to a specified baseline that characterizes its behavior in the absence of climate change. In this definition climate change refers to any long-term trend in climate, irrespective of its cause. To meet the definition of “impact attribution”’ provided in the Chapter 18 of the IPCC[42], the impact of climate change against a non-stationary baseline with temporally varying exposure and vulnerability needs be estimated by comparing observed damages trends against damages in a simulated counterfactual world where exposure and vulnerability vary according to observations but climate is assumed to be stationary.

However, since such an attribution scenario set-up has only recently been added to the ISIMIP3a protocol[43] and associated model simulations are not yet available, we here propose an alternative quantification of climate induced damages where climate induced trends are separated from socio-economic changes by keeping socio-economic drivers constant. We quantify the contribution of climate variability 1980-2010 (or the period 1971-2010) to the expected value of damage in 2010 as the difference between the start level of the trend estimation (1971 or 1980) and its end level (2010) (Equation (5) and Supplementary Table 3). This approach is as close as possible to the definition of attribution in Chapter 18 of the IPCC AR5. As our general modeling approach permits to fix exposure and vulnerability, we can compare climate induced trends as obtained from 1980 fixed exposure with the trends obtained for 2010 fixed exposure. The comparison of the climate-induced trends can be understood as a sensitivity analysis regarding the selection of the baseline scenario. Differences arise when there are assets in 2010 in areas where no assets at all existed in 1980. Assuming 2010-fixed-exposure all damages that were caused on these assets contribute to the climate-induced trend, while changes in damages on these assets in the 1980-fixed-exposure are attributed to the exposure trends. The results of the sensitivity analysis clearly reveal that the derived climate induced trends are robust with regard to the choice of the base year for the exposure.

In follow-up studies based on ISIMIP3a hydrological simulations, the availability of a counterfactual climate scenario will allow for assessments which are independent of the choice of the baseline and allow to clearly distinguish whether impacts on assets are the consequence of shifting climate conditions or of new settlements in flood affected areas.

5 Supplementary Discussion

Limitations and uncertainties

The damage modeling applied in this study shows varying quality across regions (Fig. 2). The NAM region, where the observed damage variability can be almost entirely captured by the modeling chain, highlights the general power of the modeling approach. Resolving the reasons for lower explained variances in other regions will be an important next step to improve the proposed approach as a tool for climate impact attribution. Deficits could be introduced along the entire modeling chain ranging from i) observational climate forcing data whose quality varies by region, ii) translation of daily climate data into discharge, which could arise due to inadequate process-understanding or inadequate representation of direct human influences, iii) translation of discharge into flooded areas, iv) derivation of exposed assets and translation into damages, and v) deficits of reported damages. Here, we discuss sources of uncertainties step-by-step with a focus on potential biases of the climate-induced trends in damages being central for this study:

i) Climate reanalysis data constrain the performance of GHMs and may partly be responsible for the spatial variation in explanatory power[44, 45], e.g., due to spatially and temporarily heterogenous global data coverage. However, the good agreement between observed discharge trends and the median discharge trends derived from the model simulations forced by four different observational data indicates that the uncertainties do not lead to large scale systematic biases in trends (Supplementary Notes 1).

ii) There are several sources of uncertainty associated with the translation of the climate forcing into discharge. They may refer to the exact timing and scaling of peak-runoff[44, 46], and the translation into spatially explicit flood-depth on the basis of Digital Elevation Models that are known to be fraught with high levels of uncertainty[47]. Spatial variations in GHM performance may explain part of the spatial variation in the correlations between observed and simulated damages. Thus, validation studies of the GHMs included in ISIMIP2a indicate a variation in performance across hydrobelts[44, 46] with a better performance in the wetter equatorial and northern hydrobelts than in drier southern hydrobelts[46]. This is partly reflected in our results, as we observe very low explanatory power in regions such as North Africa and better explanatory power in Asian and North American regions or subregions. In addition GHMs show a tendency to overestimate peak-runoff[44, 48]. However, accounting for floodplain storage and backwater effects as implemented in CaMa-flood, that is also applied in this study, leads to a general reduction of peak river discharge and to a better agreement between observed and simulated annual peak discharge[48]. In general the uncertainties of hydrological modeling discussed here are not expected to introduce systematic biases in trends, as modeled and observed discharge trends show very similar patterns (Supplementary Notes 1).

iii) Regarding the exact modeling of flood extents and flooded areas, the river routing model CaMa-flood has been validated in many studies[49, 50, 51], but not yet within the ISIMIP2a context. Previous studies on selected river basins in Nigeria and Mozambique revealed performance differences ranging from poor to good across river basins and a strong dependence on the return period of input flows[50]. The representation of flooded areas may particularly depend on the adequate representation of flood protection levels only coarsely known and implemented based on FLOPROS in this study. We quantify the sensitivity of damages using two different

data versions, the merged-layer (Fig. 2-4) and the modeled-layer of FLOPROS (Supplementary Fig. 7 and 8). Overall, our main findings are robust (explained variances and significance, sign and amplitude of climate-induced trends) with regard to the choice of the FLOPROS layer. Compared to the merged layer, we find slightly higher explained variances in several less developed regions such as LAM and SEA but slightly lower explained variances in NAM when using the modeled-layer of FLOPROS (cf. Fig. 2 to Supplementary Fig. 7). These differences are most likely rooted in an overestimation of policy standards in less developed areas that were used as fill-values for missing data on implemented protection standards in the merged layer. The sensitivity does not address potential biases that could be induced by temporally changes in protection levels not captured by FLOPROS only providing a snapshot. Instead of the explicit modeling through protection levels this effect is captured by the temporal variation in the derived vulnerability factors (Supplementary Fig. 1 and 2).

iv) In this study, we observe a tendency of overestimating damages in low-income areas (e.g., SSA) and of underestimating damages in high-income regions (e.g., OCE) (Supplementary Fig. 1 and 2). The major reasons here are the continental depth-damage functions[52] that assume less vulnerability in high-income countries and the protection standards in the FLOPROS database that are much higher in developed countries. However, it is important to note that these only affect the quantification of the vulnerability trends and do not affect the significance, directions, and magnitude of the climate-induced trends.

v) Finally, the reported damages from the NatCatSERVICE database cannot be assumed to be free of biases[53]. However, a likely underreporting in early years will not introduce systematic biases in the estimation of climate-induced trends, but may affect the time-variable vulnerability estimate.

It is expected that it will become possible to explicitly address iii) and v) when extending the analysis to include the last decade. Thus v) could be addressed by checking whether the correlation between observed and simulated damages increases with time indicating that discrepancies found here are reduced by improved reporting of damages. In addition, simulations of flooded areas could be evaluated based on high-quality satellite data becoming available for 1985 onwards.

Our analysis on the relative importance of different teleconnections and GMT may be limited by the fact that climate oscillations are not completely uncorrelated from changes in GMT, i.e., our predictors cannot be assumed to be completely independent. Therefore, we caution in the main text that the selection of GMT as a relevant driver does not necessarily rule out the additional relevance of climate oscillations.

6 Supplementary References

1. Munich Re. *NatCatSERVICE Database (Munich Reinsurance Company, Geo Risks Research, Munich)*. (2016).
2. GRDC. WMO Basins and Sub-Basins / Global Runoff Data Centre, GRDC. 3rd, rev. ext. ed. Koblenz, Germany: Federal Institute of Hydrology (BfG). (2020).
3. Dirmeyer, P. A. *et al.* GSWP-2: Multimodel analysis and implications for our perception of the land surface. *Bull. Am. Meteorol. Soc.* **87**, 1381–1397 (2006).
4. Sheffield, J., Goteti, G. & Wood, E. F. Development of a 50-Year High-Resolution Global Dataset of Meteorological Forcings for Land Surface Modeling. *J. Clim.* **19**, 3088–3111 (2006).
5. Weedon, G. P. *et al.* Creation of the WATCH Forcing Data and Its Use to Assess Global and Regional Reference Crop Evaporation over Land during the Twentieth Century. *J. Hydrometeorol.* **12**, 823–848 (2011).
6. Weedon, G. P. *et al.* The WFDEI meteorological forcing data set: WATCH Forcing Data methodology applied to ERA-Interim reanalysis data. *Water Resour. Res.* **50**, 7505–7514 (2014).
7. Leng, G., Huang, M., Tang, Q. & Leung, L. R. A modeling study of irrigation effects on global surface water and groundwater resources under a changing climate. *J. Adv. Model. Earth Syst.* **7**, 1285–1304 (2015).
8. Tang, Q., Oki, T., Kanae, S. & Hu, H. Hydrological Cycles Change in the Yellow River Basin during the Last Half of the Twentieth Century. *J. Clim.* **21**, 1790–1806 (2008).
9. Hanasaki, N. *et al.* An integrated model for the assessment of global water resources – Part 2: Applications and assessments. *Hydrol. Earth Syst. Sci.* **12**, 1027–1037 (2008).
10. Hanasaki, N., Yoshikawa, S., Kakinuma, K. & Kanae, S. A seawater desalination scheme for global hydrological models. *Hydrol. Earth Syst. Sci.* **20**, 4143–4157 (2016).

11. Hanasaki, N., Yoshikawa, S., Pokhrel, Y. & Kanae, S. A global hydrological simulation to specify the sources of water used by humans. *Hydrol. Earth Syst. Sci.* **22**, 789–817 (2018).
12. Clark, D. B. *et al.* The Joint UK Land Environment Simulator (JULES), model description – Part 2: Carbon fluxes and vegetation dynamics. *Geosci. Model Dev.* **4**, 701–722 (2011).
13. Best, M. J. *et al.* The Joint UK Land Environment Simulator (JULES), model description – Part 1: Energy and water fluxes. *Geosci. Model Dev.* **4**, 677–699 (2011).
14. Harper, A. B. *et al.* Improved representation of plant functional types and physiology in the Joint UK Land Environment Simulator (JULES v4.2) using plant trait information. *Geosci. Model Dev.* **9**, 2415–2440 (2016).
15. Jägermeyr, J. *et al.* Water savings potentials of irrigation systems: global simulation of processes and linkages. *Hydrol. Earth Syst. Sci.* **19**, 3073–3091 (2015).
16. Rost, S. *et al.* Agricultural green and blue water consumption and its influence on the global water system. *Water Resour. Res.* **44**, (2008).
17. Sitch, S. *et al.* Evaluation of ecosystem dynamics, plant geography and terrestrial carbon cycling in the LPJ dynamic global vegetation model. *Glob. Change Biol.* **9**, 161–185 (2003).
18. Schaphoff, S. *et al.* Contribution of permafrost soils to the global carbon budget. *Environ. Res. Lett.* **8**, 014026 (2013).
19. von Bloh, W., Rost, S., Gerten, D. & Lucht, W. Efficient parallelization of a dynamic global vegetation model with river routing. *Environ. Model. Softw.* **25**, 685–690 (2010).
20. Pokhrel, Y. N. *et al.* Incorporation of groundwater pumping in a global Land Surface Model with the representation of human impacts. *Water Resour. Res.* **51**, 78–96 (2015).
21. Takata, K., Emori, S. & Watanabe, T. Development of the minimal advanced treatments of surface interaction and runoff. *Glob. Planet. Change* **38**, 209–222 (2003).
22. Stacke, T. & Hagemann, S. Development and evaluation of a global dynamical wetlands extent scheme. *Hydrol. Earth Syst. Sci.* **16**, 2915–2933 (2012).

23. Guimberteau, M. *et al.* Testing conceptual and physically based soil hydrology schemes against observations for the Amazon Basin. *Geosci. Model Dev.* **7**, 1115–1136 (2014).
24. Guimberteau, M. *et al.* ORCHIDEE-MICT (v8.4.1), a land surface model for the high latitudes: model description and validation. *Geosci. Model Dev.* **11**, 121–163 (2018).
25. van Beek, L. P. H., Wada, Y. & Bierkens, M. F. P. Global monthly water stress: 1. Water balance and water availability. *Water Resour. Res.* **47**, (2011).
26. Wada, Y. *et al.* Global monthly water stress: 2. Water demand and severity of water stress. *Water Resour. Res.* **47**, (2011).
27. Wada, Y., Beek, L. P. H. van & Bierkens, M. F. P. Nonsustainable groundwater sustaining irrigation: A global assessment. *Water Resour. Res.* **48**, (2012).
28. Wada, Y., Wisser, D. & Bierkens, M. F. P. Global modeling of withdrawal, allocation and consumptive use of surface water and groundwater resources. *Earth Syst. Dyn.* **5**, 15–40 (2014).
29. Wada, Y. *et al.* Modeling global water use for the 21st century: the Water Futures and Solutions (WFaS) initiative and its approaches. *Geosci. Model Dev.* **9**, 175–222 (2016).
30. Liang, X., Lettenmaier, D. P., Wood, E. F. & Burges, S. J. A simple hydrologically based model of land surface water and energy fluxes for general circulation models. *J. Geophys. Res. Atmospheres* **99**, 14415–14428 (1994).
31. Müller Schmied, H. *et al.* Sensitivity of simulated global-scale freshwater fluxes and storages to input data, hydrological model structure, human water use and calibration. *Hydrol. Earth Syst. Sci.* **18**, 3511–3538 (2014).
32. Jenkinson, A. F. The frequency distribution of the annual maximum (or minimum) values of meteorological elements. *Q. J. R. Meteorol. Soc.* **81**, 158–171 (1955).
33. Tasker, G. Review of Regional Frequency Analysis: An Approach Based on L-Moments. *J. Am. Stat. Assoc.* **93**, 1233–1233 (1998).
34. Willner, S. N., Levermann, A., Zhao, F. & Frieler, K. Adaptation required to preserve future high-end river flood risk at present levels. *Sci. Adv.* **4**, eaao1914 (2018).

35. Hirabayashi, Y. *et al.* Global flood risk under climate change. *Nat. Clim. Change* **3**, 816–821 (2013).
36. Kim, H., Yeh, P. J.-F., Oki, T. & Kanae, S. Role of rivers in the seasonal variations of terrestrial water storage over global basins. *Geophys. Res. Lett.* **36**, (2009).
37. Scussolini, P. *et al.* FLOPROS: an evolving global database of flood protection standards. *Nat. Hazards Earth Syst. Sci.* **16**, 1049–1061 (2016).
38. Blöschl, G. *et al.* Changing climate both increases and decreases European river floods. *Nature* **573**, 108–111 (2019).
39. Do, H. X., Westra, S. & Leonard, M. A global-scale investigation of trends in annual maximum streamflow. *J. Hydrol.* **552**, 28–43 (2017).
40. Gudmundsson, L., Leonard, M., Do, H. X., Westra, S. & Seneviratne, S. I. Observed Trends in Global Indicators of Mean and Extreme Streamflow. *Geophys. Res. Lett.* **46**, 756–766 (2019).
41. Mediero, L., Santillán, D., Garrote, L. & Granados, A. Detection and attribution of trends in magnitude, frequency and timing of floods in Spain. *J. Hydrol.* **517**, 1072–1088 (2014).
42. Cramer, W. *et al.* Detection and attribution of observed impacts. *Clim. Clim. Change 2014 Impacts Adapt. Vulnerability* 979–1038 (2014) doi:10.1017/CBO9781107415379.023.
43. Mengel, M., Treu, S., Lange, S. & Frieler, K. ATTRICI 1.0 – counterfactual climate for impact attribution. *Geosci. Model Dev. Discuss.* 1–26 (2020) doi:https://doi.org/10.5194/gmd-2020-145.
44. Beck, H. E. *et al.* Global evaluation of runoff from 10 state-of-the-art hydrological models. *Hydrol. Earth Syst. Sci.* **21**, 2881–2903 (2017).
45. Ruane, A. C., Goldberg, R. & Chryssanthacopoulos, J. Climate forcing datasets for agricultural modeling: Merged products for gap-filling and historical climate series estimation. *Agric. For. Meteorol.* **200**, 233–248 (2015).

46. Zaherpour, J. *et al.* Worldwide evaluation of mean and extreme runoff from six global-scale hydrological models that account for human impacts. *Environ. Res. Lett.* **13**, 065015 (2018).
47. Yamazaki, D., Kanae, S., Kim, H. & Oki, T. A physically based description of floodplain inundation dynamics in a global river routing model. *Water Resour. Res.* **47**, (2011).
48. Zhao, F. *et al.* The critical role of the routing scheme in simulating peak river discharge in global hydrological models. *Environ. Res. Lett.* **12**, 075003 (2017).
49. Bernhofen, M. V. *et al.* A first collective validation of global fluvial flood models for major floods in Nigeria and Mozambique. *Environ. Res. Lett.* **13**, 104007 (2018).
50. Ikeuchi, H. *et al.* Modeling complex flow dynamics of fluvial floods exacerbated by sea level rise in the Ganges–Brahmaputra–Meghna Delta. *Environ. Res. Lett.* **10**, 124011 (2015).
51. Mateo, C. M. R. *et al.* Impacts of spatial resolution and representation of flow connectivity on large-scale simulation of floods. *Hydrol. Earth Syst. Sci.* **21**, 5143–5163 (2017).
52. Huizinga, J., De Moel, H. & Szewczyk, W. *Global flood depth-damage functions: Methodology and the database with guidelines*. https://publications.jrc.ec.europa.eu/repository/handle/111111111/45730 (2017).
53. Guha-Sapir, D. & Below, R. Quality and accuracy of disaster data: A comparative analyse of 3 global data sets. *Work. Pap. Prep. Disaster Manag. Facil. World Bank Bruss.* (2002).
