# Supplementary material for: Climate signals in river flood damages emerge under sound regional disaggregation
Source: Nat Commun. 2021 Apr 9;12:2128. doi: 10.1038/s41467-021-22153-9 (PMC8035337; doi:10.1038/s41467-021-22153-9)
Supplement: Supplementary file 3 — Description of Additional Supplementary Files [file 41467_2021_22153_MOESM3_ESM.pdf]

Description of Additional Supplementary Files

File name: Supplementary Data 1

Description: Data supporting all the findings and results presented in the figures of the study. This includes all the result metrics on regional level presented in the figures and modeled damage time series on regional and country level.
