# Supplementary material for: Climate signals in river flood damages emerge under sound regional disaggregation
Source: Nat Commun. 2021 Apr 9;12:2128. doi: 10.1038/s41467-021-22153-9 (PMC8035337; doi:10.1038/s41467-021-22153-9)
Supplement: Supplementary file 4 — Supplementary Data 1 [file 41467_2021_22153_MOESM4_ESM.zip › supplementary_data/supplementary_data_description.pdf]

Supplementary Data Description

Climate signals in river flood damages emerge under sound regional disaggregation

Inga J. Sauer[1,2], Ronja Reese[1], Christian Otto[1*], Tobias Geiger[1,3], Sven N. Willner[1], Benoit Guillod[2,4], David N. Bresch[2,5] , and Katja Frieler[1*]

1 Potsdam Institute for Climate Impact Research, Telegraphenberg A 56, 14473 Potsdam, Germany
2 Institute for Environmental Decisions, ETH Zurich, Universitätstr. 22, 8092 Zurich, Switzerland
3 Deutscher Wetterdienst (DWD), Climate and Environment Consultancy, Güterfelder Damm 87-91, 14532 Stahnsdorf, Germany
4 Institute for Atmospheric and Climate Science, ETH Zurich, Universitätstr. 22, 8092 Zurich, Switzerland
5 Federal Office of Meteorology and Climatology MeteoSwiss, Operation Center 1, P.O. Box 257, 8058 Zurich-Airport, Switzerland

*e-mail: christian.otto@pik.potsdam.de; katja.frieler@pik-potsdam.de

The dataset provides uncalibrated modeled flood damages on annual resolution for the time period (1971-2010) from 46 combinations of GHMs and climate forcing data sets on the country level. Included are time series accounting for varying exposure and varying climate conditions and time series accounting only for varying climate conditions and fixed exposure conditions from the years 1980 and 2010. Additionally, we provide calibrated aggregated damage time series for the world regions used in the main article for the time period 1980-2010. Finally, the data set includes several metrics from the main analysis supporting the findings of the article including the contributions of different drivers to overall economic damage trends an explanatory power of models. For all datasets we provide damages aggregated to the country or to the regional level and damages separated for subareas with either only increasing hazards or only decreasing hazards as described in the main article. This document lists the content of the files and the meaning of each column and the given units.

Content

Country level damages (modeled) 2
Damage time series aggregated to the regional level (model ensemble median) 3
Result metrics (regional level) 4

Country level damages (modeled)

Data Description Table 1: Units and description of the variables provided in the files *country_damages_multimodel_R.csv* and *country_damages_multimodel_R_pos_R_neg.csv* provided in the Supplementary Data. The files include modeled damages on the country level for all combinations of climate forcings and global hydrological models (GHM).

Column name	Description	Unit
**Year**	Year of damage assessment	--
**Country**	ISO3 code of the country	ISO3
**Region**	World region (OCE, CAS, EAS, EUR, GLB, LAM, NAF, NAM, SSA, SEA)	--
**D_CliExp_raw***	Modeled damage when accounting for varying exposure and varying climate conditions. The damages are **not calibrated** to the observed damage.	inflation adjusted 2005 purchasing power parities (PPP) USD
**D_1980_raw ***	Modeled damage when accounting for varying climate conditions using fixed 1980 socio-economic conditions. The damages are **not calibrated** to the observed damage.	inflation adjusted 2005 (PPP) USD
**D_2010_raw ***	Modeled damage when accounting for varying climate conditions using fixed 2010 socio-economic conditions. The damages are **not calibrated** to the observed damage.	inflation adjusted 2005 (PPP) USD
**GHM**	Name of the GHM used to force CaMa-Flood.	--
**clim_forc**	Name of the climate forcing dataset used to drive the GHM.	--

* In the file *country_damages_multimodel_R_pos_R_neg.csv* the column name is complemented by the ending *_pos* and*_neg* indicating that in the column only damage in river basins with a positive (R_+) or with a negative discharge trend (R_-) is considered.

Damage time series aggregated to the regional level (model ensemble median)

Data Description Table 2: Units and description of the variables provided in the files *region_damages_modelmedian_R.csv* and *region_damages_modelmedian_R_pos_R_neg.csv* provided in the Supplementary Data. The files include the ensemble median of the modeled damages aggregated across world regions (Fig. 2).

Column name	Description	Unit
**Year**	year for which damage was assessed	--
**Region**	World region (OCE, CAS, EAS, EUR, GLB, LAM, NAF, NAM, SSA, SEA)	
**ratios_D_Obs_D_CliExp***	Vulnerability ratio (D_{Obs} / D_{CliExp})[1]	--
**vulnerability_function ***	Vulnerability function (D_{Obs} / D_{CliExp} smoothed with Singular Spectrum Analysis)	--
**D_Full***	Modeled damage when accounting for varying vulnerability, exposure and climate conditions. The damage is **calibrated** to meet the total observed damage of the period 1980-2010. **(Model median)**	inflation adjusted 2005 (PPP) USD
**D_CliExp***	Modeled damage when accounting for varying exposure and varying climate conditions. The damage time series is **normalized** to the starting level of D_Full in 1980. **(Model median)**	inflation adjusted 2005 (PPP) USD
**D_1980***	Modeled damage when accounting only for varying climate conditions assuming 1980 socio-economic conditions. The damage time series is **normalized** to the starting level of D_Full in 1980. **(Model median)**	inflation adjusted 2005 (PPP) USD
**D_2010***	Modeled damage when accounting only for varying climate conditions assuming 2010 socio-economic conditions. The damage time series is **normalized** to the end level of D_Full in 2010. **(Model median)**	inflation adjusted 2005 (PPP) USD
**D_Full_1thrd_quantile***	Modeled damage when accounting for varying vulnerability, exposure and climate conditions. The damage is **calibrated** to meet the total observed damage of the period 1980-2010. Upper limit of the 1/3 quantile of the entire model ensemble.	inflation adjusted 2005 (PPP) USD
**D_Full_2thrd_quantile***	Modeled damage when accounting for varying vulnerability, exposure and climate conditions. The damage is **calibrated** to meet the total observed damage of the period 1980-2010. Upper limit of the 2/3 quantile of the entire model ensemble.	inflation adjusted 2005 (PPP) USD
**D_CliExp_norm_for_trend***	Modeled damage when accounting for varying exposure and varying climate conditions. The damage time series is **normalized** to total observed damage to permit comparable results in the trend analysis. **(Model median)**	inflation adjusted 2005 (PPP) USD
**D_1980_norm_for_trend***	Modeled damage when accounting only for varying climate conditions assuming 1980 socio-economic conditions. The damage time series is **normalized** to total observed damage to permit comparable results in the trend analysis. **(Model median)**	inflation adjusted 2005 (PPP) USD
**D_2010_norm_for_trend***	Modeled damage when accounting only for varying climate conditions assuming 2010 socio-economic conditions. The damage time series is **normalized** to total observed damage to permit comparable results in the trend analysis. **(Model median)**	inflation adjusted 2005 (PPP) USD

* In the file *region_damages_modelmedian_R_pos_R_neg.csv* the column name is complemented by the endings *_pos* and *_neg* indicating that in the column only damage in river basins with a positive (R_+) or with a negative discharge trend (R_-) is considered.

[1] Note that D_{Obs} refers to observed damages from NatCatSERVICE, which cannot be provided in this dataset and need to be requested directly from Munich Re.

Result metrics (regional level)

Data Description Table 3: Units and description of the variables provided in the files *region_result_metrics_R.csv* and *region_result_metrics_ R_pos_R_neg.csv* provided in the Supplementary Data. The files include the result metrics related to the results shown in Fig.3 - 5 and in the Supplementary Information. The metrics provided in column 2-8 provide information on the capacity of the modeled time for reproducing observed data and a summary on observational records. Column 9 – 24 provide all metrics of the trend analysis and the contributions of different drivers. These are basically the results given in Fig. 3 and 4 of the main article and Supplementary Table 3. Column 25 -60 are metrics related to the contribution of GMT and climate oscillations to climate induced trends. Column 25 - 42 present metrics for runs with AMO, ENSO, PDO and NAO as predictors. Column 43 - 60 present metrics for runs with AMO, ENSO, PDO and NAO as predictors. Included here are only metrics for the best selected model of both runs.

	Column name	Description	Unit
1	**Region**	World region (OCE, CAS, EAS, EUR, GLB, LAM, NAF, NAM, SSA, SEA). In region*_result_metrics_ R_pos_R_neg.csv* the R_+ and R_- subregion are given in the column as (OCE_pos, OCE_neg, CAS_pos, CAS_neg, …)	--
2	**R2_D_1980_D_Obs**	Explained variance R^2 (square of the pearson correlation coefficient) when D_{1980} and D_{Obs} are correlated.	%
3	**R2_D_CliExp_D_Obs**	Explained variance R^2 (square of the pearson correlation coefficient) when D_{CliExp} and D_{Obs} are correlated.	%
4	**R2_D_Full_D_Obs**	Explained variance R^2 (square of the pearson correlation coefficient) when D_{Full} and D_{Obs} are correlated.	%
5	**total_observed_damages**	Total observed damage (1980-2010)	inflation adjusted 2005 (PPP) USD
6	**annual_mean_damage_obs**	Annual mean observed damage (1980-2010)	inflation adjusted 2005 (PPP) USD
7	**std_dev_obs**	Standard deviation of annual observed damage (1980-2010)	inflation adjusted 2005 (PPP) USD
8	**mean_vulnerability**	Mean smoothed ratio $D_{Obs}/D_{CliExp.}$ (1980-2010).	--
9	**C_1980_80**	Sen-slope in D_{1980} (1980-2010), normalized to the annual mean observed damage (1980-1995)	% per year
10	**p_val_C_1980_80**	p-val of the Mann-Kendall Test in D_{1980} (1980-2010)	--
11	**C_2010_80**	Sen-slope in D_{2010} (1980-2010), normalized to the annual mean observed damage (1980-1995)	% per year
12	**p_val_C_2010_80**	p-val of the Mann-Kendall Test in D_{2010} (1980-2010)	
13	**C_1980_71**	Sen-slope in D_{1980} (1971-2010), normalized to the annual mean observed damage (1980-1995)	% per year
14	**p_val_C_1980_71**	p-val of the Mann-Kendall Test in D_{1980} (1971-2010)	
15	**C_2010_71**	Sen-slope in D_{2010} (1971-2010), normalized to the annual mean observed damage (1980-1995)	% per year
16	**p_val_C_2010_71**	p-val of the Mann-Kendall Test in D_{2010} (1971-2010)	--
17	**E_exposure**	Sen-slope in D_{1980} (1980-2010) - Sen-slope in D_{CliExp} (1980-2010), normalized to the annual mean observed damage (1980-1995).	% per year
18	**V_vulnerability**	Sen-slope in D_{1980} (1980-2010) substracted from the Sen-slope in D_{CliExp} (1980-2010), normalized to the annual mean observed damage (1980-1995).	% per year
19	**M_damage_modeled**	Sen-slope in D_{Full} (1980-2010), normalized to the annual mean observed damage (1980-1995)	% per year
20	**p_val_M_damage_modeled**	p-val of the Mann-Kendall Test in D_{Full} (1980-2010)	--
21	**N_damage_observed**	Sen-slope in D_{Obs} (1980-2010), normalized to the annual mean observed damage (1980-1995)	% per year
22	**p_val_N_damage_observed**	p-val of the Mann-Kendall Test in D_{Obs} (1980-2010)	--
23	**delta_2010_since80**	Additional damage or damage reduction in 2010 due to changes in climate since 1980. Expressed as percentage of annual mean observed damage (1980-1995).	%
24	**delta_2010_since71**	Additional damage or damage reduction in 2010 due to changes in climate since 1971. Expressed as percentage of annual mean observed damage (1980-1995).	%
25	**pval_ENSO_amo_run**	Significance of the ENSO index. The GLM here used AMO, NAO, ENSO and PDO as predictors.	--

26	**gammaAbs_ENSO_amo_run°**	Partial derivative of the GLM with regard to the predictor ENSO. The GLM here used AMO, NAO, ENSO and PDO as predictors.	-- (data was centered and scaled previously)
27	**pval_ENSOlag_amo_run**	Significance of the ENSO index with one year time lag. The GLM here used AMO, NAO, ENSO and PDO as predictors.	--
28	**gammaAbs_ENSOlag_amo_run°**	Partial derivative of the GLM with regard to the predictor ENSO with one year time lag. The GLM here used AMO, NAO, ENSO and PDO as predictors.	-- (data was centered and scaled previously)
29	**gammaAbs_AMO_amo_run°**	Partial derivative of the GLM with regard to the predictor AMO. The GLM here used AMO, NAO, ENSO and PDO as predictors.	-- (data was centered and scaled previously)
30	**pval_AMO_amo_run**	Significance of the AMO index. The GLM here used AMO, NAO, ENSO and PDO as predictors.	--
31	**gammaAbs_PDO_amo_run°**	Partial derivative of the GLM with regard to the predictor PDO. The GLM here used AMO, NAO, ENSO and PDO as predictors.	-- (data was centered and scaled previously)
32	**pval_PDO_amo_run**	Significance of the PDO index. The GLM here used AMO, NAO, ENSO and PDO as predictors.	--
33	**pval_PDOlag_amo_run**	Significance of the PDO index with one year time lag. The GLM here used AMO, NAO, ENSO and PDO as predictors.	-- (data was centered and scaled previously)
34	**gammaAbs_PDOlag_amo_run°**	Partial derivative of the GLM with regard to the predictor PDO with one year time lag. The GLM here used AMO, NAO, ENSO and PDO as predictors.	-- (data was centered and scaled previously)
35	**pval_NAO_amo_run**	Significance of the NAO index. The GLM here used AMO, NAO, ENSO and PDO as predictors.	--
36	**gammaAbs_NAO_amo_run°**	Partial derivative of the GLM with regard to the predictor NAO. The GLM here used AMO, NAO, ENSO and PDO as predictors.	-- (data was centered and scaled previously)
37	**pval_NAOlag_amo_run**	Significance of the NAO index with one year time lag. The GLM here used AMO, NAO, ENSO and PDO as predictors.	--
38	**gammaAbs_NAOlag_amo_run°**	Partial derivative of the GLM with regard to the individual predictor with one year time lag. The GLM here used AMO, NAO, ENSO and PDO as predictors.	-- (data was centered and scaled previously)
39	**pval_residual_trend_amo_run**	Significance of the trend in the residuals of the prediction of the best GLM and the potential predictors AMO, NAO, ENSO and PDO and $D_{1980.}$	--
40	**residual_trend_amo_run**	Trend in the residuals of the prediction of the best GLM with the potential predictors AMO, NAO, ENSO and PDO and $D_{1980.}$	-- (data was centered and scaled previously)
41	**R2_D_1980_bm_amo_run**	Explained variance of D_{1980} by the best GLM with the potential predictors AMO, NAO, ENSO and PDO.	%
42	**oose_bm_amo_run**	Out-of-sample-error of the best GLM with the potential predictors AMO, NAO, ENSO and PDO.	-- (data was centered and scaled previously)
43	**pval_ENSO_gmt_run**	Significance of the ENSO index. The GLM here used GMT, NAO, ENSO and PDO as predictors	--
44	**gammaAbs_ENSO_gmt_run°**	Partial derivative of the GLM with regard to the predictor ENSO. The GLM here used GMT, NAO, ENSO and PDO as predictors.	-- (data was centered and scaled previously)

45	**pval_ENSOlag_gmt_run**	Significance of the ENSO index with one year time lag. The GLM here used GMT, NAO, ENSO and PDO as predictors.	--
46	**gammaAbs_ENSOlag_gmt_run°**	Partial derivative of the GLM with regard to the predictor ENSO with one year time lag. The GLM here used GMT, NAO, ENSO and PDO as predictors.	-- (data was centered and scaled previously)
47	**gammaAbs_GMT_gmt_run°**	Partial derivative of the GLM with regard to the predictor GMT. The GLM here used GMT, NAO, ENSO and PDO as predictors.	-- (data was centered and scaled previously)
48	**pval_GMT_gmt_run**	Significance of the GMT index. The GLM here used GMT, NAO, ENSO and PDO as predictors.	
49	**gammaAbs_PDO_gmt_run°**	Partial derivative of the GLM with regard to the predictor PDO. The GLM here used GMT, NAO, ENSO and PDO as predictors.	-- (data was centered and scaled previously)
50	**pval_PDO_gmt_run**	Significance of the PDO index. The GLM here used GMT, NAO, ENSO and PDO as predictors.	--
51	**pval_PDOlag_gmt_run**	Significance of the PDO index with one year time lag. The GLM here used GMT, NAO, ENSO and PDO as predictors.	--
52	**gammaAbs_PDOlag_gmt_run°**	Partial derivative of the GLM with regard to the predictor PDO with one year time lag. The GLM here used GMT, NAO, ENSO and PDO as predictors.	-- (data was centered and scaled previously)
53	**pval_NAO_gmt_run**	Significance of the NAO index. The GLM here used GMT, NAO, ENSO and PDO as predictors.	--
54	**gammaAbs_NAO_gmt_run°**	Partial derivative of the GLM with regard to the predictor NAO. The GLM here used GMT, NAO, ENSO and PDO as predictors.	-- (data was centered and scaled previously)
55	**pval_NAOlag_gmt_run**	Significance of the NAO index with one year lag. The GLM here used GMT, NAO, ENSO and PDO as predictors.	--
56	**gammaAbs_NAOlag_gmt_run°**	Partial derivative of the GLM with regard to the predictor NAO with one year time lag. The GLM here used GMT, NAO, ENSO and PDO as predictors.	-- (data was centered and scaled previously)
57	**pval_residual_trend_gmt_run**	Significance of the trend in the residuals of the prediction of the best GLM and the potential predictors GMT, NAO, ENSO and PDO and D_{1980}.	--
58	**residual_trend_gmt_run**	Trend in the residuals of the prediction of the best GLM with the potential predictors GMT, NAO, ENSO and PDO and D_{1980}.	-- (data was centered and scaled previously)
59	**R2_D_1980_bm_gmt_run**	Explained variance of D_{1980} by the best GLM with the potential predictors GMT, NAO, ENSO and PDO.	%
60	**oose_gmt_run**	Out-of-sample-error of the best GLM with the potential predictors GMT, NAO, ENSO and PDO.	-- (data was centered and scaled previously)

°In order to derive γ as presented in Fig. 5 of the main text from the given variable it needs to be divided by the sum of all absolute values for all predictors in the model.
