# Supplementary material for: Climate signals in river flood damages emerge under sound regional disaggregation
Source: Nat Commun. 2021 Apr 9;12:2128. doi: 10.1038/s41467-021-22153-9 (PMC8035337; doi:10.1038/s41467-021-22153-9)
Supplement: Supplementary file 2 — Peer Review File [file 41467_2021_22153_MOESM2_ESM.pdf]

Reviewers' Comments:

Reviewer #1:
Remarks to the Author:
Dear Authors,
The manuscript „Climate signals in river flood damages emerge under sound regional disaggregation" is an important contribution to the debate on flood damage attribution in a global climate-change context. In general it is well-written with informative figures; it comprehensively discusses the results and uncertainty in those results. The study makes the most of available global hydrological and exposure data. I think that those two components of risk were done very well, however I have concerns over the final element, namely vulnerability. The available descriptions and figures cause doubts if vulnerability was calculated correctly. More specifically:
1) The description of converting reported flood damages to a single benchmark (L372-378) is not fully clear and suggests that they may not be converted properly to make them comparable with exposure data. The fastest way to resolve this would be for the authors to show an example calculation for a single event: flood in the UK and Germany on 4-7 January 1982 with losses of 700 mln USD in nominal prices (according to NatCatSERVICE).
2) Later the authors describe how reported losses were aggregated (L378-384). The text states that "we…assign the damage to the risk area of the centroid closest to the geocode location." What is this risk area? Is it a continuous flood extent, and the risk area corresponding to the event is connected with the nearest patch among the various patches of flood extents? NatCatSERVICE is usually is rather imprecise in defining flood extents, like in the abovementioned example where it is given as "United Kingdom, Germany" (at least in the publicly-available website, but I guess the authors have access also to non-public information?). Wouldn't it make more sense to include the flood extents covering the whole 'political' area defined in the NatCatSERVICE rather than potentially incorrectly assigning the flood to a small flood extent patch?
3) Related to the above: the vulnerability estimates in Figs. SI1 and SI2 for developed regions (NAM, WEU, OCE) show vastly higher reported losses compared with observations compared with other parts of the world. I wonder whether the use of FLOPROS flood protection data is the reason here. With a 40-year observation period for discharges there will be not many floods to exceed, say, a 100-year return period. Meanwhile, large parts of NAM and WEU have 100-year or more protection standards according to FLOPROS. Would that narrow down the flood extent and, consequently, exposure too much for the developed countries? This could also explain outliers such 2002 Germany and 2007 UK floods, which had high losses in nominally flood-protected areas. Could the authors show the calculation of vulnerability and modelled spatial extents for the 2002 flood?
4) In Fig. S1 it is noticeable that it is largely singular yearly outliers that are responsible of upward vulnerability trend in NAM and WEU. The authors use a 1000:1 ratio for removing outliers, but that is decidedly to high to capture them. Also, computing the trends the authors put together events of vastly different magnitude, which raises mainly the problem of calculating the correct flood footprint to compare with observations: small floods with broadly defined country-level extent will show little vulnerability and big events will show much higher vulnerability. NatCatSERVICE is hardly comprehensive and varies significantly in precision of flood extent definitions as country or sub-country units. Given those limitations, I don't think it makes sense to use a complex time-varying smoothing method. A simple trend over all data points would improve the confidence in the results somewhat. Otherwise, the authors would need to improve the homogeneity of the vulnerability data.
In summation, the authors need to provide better evidence that their vulnerability calculation is robust enough for inclusion in the analysis. This is the main finding of my review. Some other comments of mine are only minor textual suggestions:
L27-L32: I find the conclusions in the abstract difficult to understand without reading the paper first, as the first sentence seems to contradict the second, leaving the reader confused whether there was a climate change signal detected or not. Consider rewriting for better clarity.
L41: should be "are extreme precipitation events".
L42: break the sentence into two at ", and"

L48: should be "1980s"
L50: change the second "and" to ", with"
Fig. 2: I think the time series graphs are not really helpful in the main paper, as the logarithmic scale makes them anyway look very similar. I suggest moving them to the Supplement and leaving only the bar graphs which are one of the principal findings of the paper, while being at present only squeezed on the side. A full-size figure with only the bar graphs, all with the same vertical scale for comparability, would be better here. Alternatively, the figure could be split into two (line graph and bar graph).
L159: space missing after "R+"
L351: the data are "re-aggregated" to 2.5 arcmin resolution. What was the original resolution then? Also, using the apostrophe (‵) to denote arc minutes would be better.
L360: please indicate the resolution of the grid.
L389: 150 arcmin resolution? That's 2.5 degrees. Is that correct?
Eq. 1: what does "R" denote? What is "Cli-R1980" for instance?
Eq. 1-4: single letters should be used to denote variables in equations; please rewrite them, using appropriate sub-indices where necessary.

I am looking forward to your revision of the manuscript.

Best regards,
Dominik Paprotny

Reviewer #2:
Remarks to the Author:
Manuscript ID# NCOMMS-20-24884-T
Journal: Nature Communications (ISSN: 2041-1723)
Title: Climate signals in river flood damages emerge under sound regional disaggregation

Dear Authors,

The submitted manuscript presents trends (if any) in flood damages at regional/sub-regional scales and their associations with atmospheric/oceanic teleconnections conditioned on socio-economic exposure dynamics.
While there is a dearth of literature that focuses on incorporating multiple data sources (i.e., observational and model-based inputs) for local-to-regional scales of flood damages (risk) assessment across the world, I see a great piece of contribution to the field throughout this work. The readers of Nature Communications with varying backgrounds will benefit to a great extent as this work brings into front the matter of flood damage assessment through the exhibition of a homogenous framework by incorporating exposure, vulnerability, economic information, and climate contributions.
However, I would recommend that the Authors should be more cautious when trying to explain the observed trends and their relationships with climate/exposure/vulnerability information. Most of the discussions are centered around a relatively low-to-moderate level of explaining the variance (R2 of 30% or higher) or a 90% significance level (with linear formulation of damages against the teleconnection indices and an evident significant degree of uncertainty). At the same time, I am completely aware that a low-to-moderate level of R2 for such analysis at a global scale might be sufficiently reasonable. Still, different sources of uncertainties exist when the spatial scale shifts to a region/sub-region scale. I should also underline here that I appreciate the Authors to report some of the critical issues related to the employed flood damages information (e.g., L102–104, L225–232). All that said, this is a well-structured manuscript with a clear storyline and informative illustrations. I am providing a list of major and minor concerns that hopefully will elevate the current scientific soundness and consistency level of this manuscript, as follows:

Major Comments:

I. Unexplained climate-driven or socio-economic driven forcing manifested in flood damages: I was not able to find a concise (and complete) discussions related to this fact the how much variability in each region/sub-region was not able to be captured by either of climate or socio-economic inputs. For instance, how significant was the trend in the remaining residuals in Fig. 4? Did the time-series in each sub-region examine for auto-correlation? Was there a significant trend observed in the residuals of those regions with significant predictors as well? What will be the justifications to explain the negative/positive sign of alpha (Eq. 3–4) for different teleconnections, or proposing a linear model of drivers (without interaction term(s), lag, or higher order of drivers, or embedded information in GMT)? Please add more pieces of information in this regard to the manuscript (Methods or Fig. 4 discussions and/or last paragraph of the Conclusions section).

II. Conditioning on upward/downward trend in discharge: To my understanding, most of the analysis and attributions are performed conditioned on observing a pattern (if any) in discharge over a region/sub-region (Fig. 3?). What do the Authors think for the places such as EAS where both R+ and R- trigger the damages, while the exposure inevitably has increased, and thus the climate shows a significant contribution? I acknowledge that you have considered two fixed exposures (1980 and 2010) to get the relative metric, still how you will explain or quantify the contribution from the changes in climate between these two periods? I presume it will provide more insights to answer this question such that how different was the exposure (or vulnerability) between these two periods per region/sub-region on average?

Minor Comments:

1. L47: "1980s"
2. L94–95: Please define "all three drivers" here. I acknowledge that you have indicated them in the caption of Fig. 2 and the next lines in this section. Still, it will be better to mention those three drivers when you specifically start to discuss them in the text.
3. L102–104: I would suggest moving these lines to the corresponding Methods section(s) to avoid any interruption within the flow of contents.
4. L178: Is it possible to move the panel for Global (and the legend) to somewhere at the top of this figure? In that case, it will be more consistent with Fig. 2, and the reader will be more comfortable.
5. L237–238: How many events were available in the observed database for individual sub-regions?
6. L288–290: Although we should acknowledge that the observational/model outputs feature a low-to-moderate level of quality across these regions.
7. L313–314: Is there any information regarding the sample size or its range over each region/sub-region? While I am aware of the spatial resolution (in different arcmin scales) that you have mentioned in this section, it will make more sense to have such information in numbers/ranges per region/sub-region.
8. L359 and L361 and similar places throughout the manuscript: The word "data" should be used plural: "Gridded GDP data were obtained…", "Downscaled GDP data are available…" and so on.
9. L381: Is that based on a calendar year?
10. L382: What does "geocodes" mean here? Are they different from the longitudinal/latitudinal (geographic) coordinates?
11. L389: I would suggest providing the corresponding scale in 'km-by-km' here and elsewhere when you mention 'arcmins' as well.
12. L404: Please remove the repeated "the" here.
13. L406: Is there a possibility to provide the exact sample size from the MunichRe database?
14. L418–419: How many missing values were there?
15. L440–443: If you are going to specify the sources of observations for the teleconnections provided by NOAA, could you please double-check the links and source of observations for the NAO indices? I assume NAO indices are available through the NOAA/CPC (not NOAA/ESRL).
16. L444: What are those three input climatic forcings? Please be specific.

17. L450: I believe Armal et al. (2018) used (multiple) multilevel regression model, which I would not recommend to cite it here and in this content.
18. L463: The residuals must be checked in terms of periodicity (if any) in addition to the remaining trends. A simple ACF can examine this matter if you have not done this testing.

Thank you, -Reviewer

Reviewer #3:
Remarks to the Author:
This study attempts to detect climate signals from the reported economic damage due to river flooding. The risk driver (i.e., economic damage) was decomposed into three factors (hazards, exposure, and vulnerability), then the trend analysis of these factors was used to detect climate signals. I have the challenging impression for this study, the idea is very good and is of interest. However, I have some questions and suggestions about the regional classification, model uncertainty and validation, calculation of time variation of vulnerability. For this reason, the manuscript can be required revisions before publication. The main points raised are as follows.

1. In this study, they divided into 18 regions based on the geographical classification and long-term trend in river discharge. Why did not the author classified based on river basin units? This long-term trend in simulated river discharge is calculated based on runoff output derived from multiple GHMs forced by bias corrected meteorological data, however the simulated river discharge have uncertainties derived from complicated model chain (e.g., rainfall patrons, land surface hydrological cycle, and digital elevation model).

2. Furthermore, the author forcibly grouped a grid with unclear long-term trend and low statistical significant (e.g., Sahel region, western part of China etc). In addition, although this study used the event location information recorded in NatCatSERVICE, it is difficult to accurately recorded the location of the event. Flood events occur in river basin unit. Therefore, the used regional classification should be based on river basin unit for more extracting robust climate signals.

3. This study validated the long-term trend of river discharge between the simulation and observation, but does not address other factors (e.g., economic damage). The CaMa-Flood, used in this study, has been validated by numerous studies, however this study used different input dataset (i.e., ISI-MIP2) with other studies, therefore the author should state validation of flood water depth and flood extension. In addition, although CLIMADA is used for the damage estimation, the author should describe how much uncertainty there is in the calculation of the economic damage.

4. Previous studies (e.g., Jongman et al., 2015; Tanoue et al., 2016) has shown that long-term variation in vulnerability decreases with income growth and over time. However, this study ignores this effect and calculates the vulnerability on the regional classification. If the vulnerability variation in FigS1 is calculated as the sum of reported and modelled economic damage, the vulnerability will be strongly influenced by countries with higher income. Therefore, vulnerability should interpolate for each year and each country, and then these aggregated. Year-national scale interpolation method of vulnerabilities have been proposed by Kinoshita et al (2018) and Dottori et al (2018).
• Dottori, F. et al. Increased human and economic losses from river flooding with anthropogenic warming. Nature Climate Change 8, 781-786, doi:10.1038/s41558-018-0257-z (2018).
• Kinoshita, Y., Tanoue, M., Watanabe, S. & Hirabayashi, Y. Quantifying the effect of autonomous adaptation to global river flood projections: application to future flood risk assessments. Environmental Research Letters 13, doi:10.1088/1748-9326/aa9401 (2018).
• Jongman, B. et al. Declining vulnerability to river floods and the global benefits of adaptation. Proc Natl Acad Sci U S A 112, E2271-2280, doi:10.1073/pnas.1414439112 (2015).

• Tanoue, M., Hirabayashi, Y. & Ikeuchi, H. Global-scale river flood vulnerability in the last 50 years. Sci Rep 6, 36021, doi:10.1038/srep36021 (2016).

Minor remarks:

1. It is unclear how the estimated vulnerability is used for calculation of economic damage (e.g., Dcliexp).

2. The vulnerability is calculated as a ratio of the reported economic damage to the modeled economic damage. I seem to be seeing some extremely high values (e.g., WEU- and OCE-), this may be because of CPI fixer. To reduce this uncertainty, I recommend the author that estimated and reported nominal values are used for the calculation of vulnerability.

3. The FLOPROS Merged Layer may be too high flood protection level for low-income countries. For example, in Bangladesh, the Policy Layer (i.e., future target protection level) is applied for all over the country. Its value is over 50 years, which is very higher than the Model Layer estimated by income level. For this reason, recent some studies (e.g., Ward et al., 2017) have used the Model Layer of FLOPROS.

• Ward, P. J. et al. A global framework for future costs and benefits of river-flood protection in urban areas. Nature Climate Change 7, 642-646, doi:10.1038/nclimate3350 (2017).

4. How did you convert the river discharge to flood water depth? CaMa-Flood diagnoses the river discharge based on the amount of total water storage including downstream cells, therefore the river discharge cannot be directly converted to flood water depth. In addition, there are sometimes negative values found in simulated river discharge in CaMa-Flood, I wonder whether the GEV parameters have been properly calculated using these values. Therefore, it may be better to estimate the return period using other variables (e.g., total water storage and flood water depth etc).

5. The axis of the bar chart on the right of Fig. 2 is barely visible.

Responses to the reviewers

Reviewer #1 (Remarks to the Author):

Dear Authors,

The manuscript „Climate signals in river flood damages emerge under sound regional disaggregation" is an important contribution to the debate on flood damage attribution in a global climate-change context. In general it is well-written with informative figures; it comprehensively discusses the results and uncertainty in those results. The study makes the most of available global hydrological and exposure data. I think that those two components of risk were done very well, however I have concerns over the final element, namely vulnerability. The available descriptions and figures cause doubts if vulnerability was calculated correctly. More specifically:

We thank the reviewer for the positive assessment of our work. We have substantially revised the manuscript and improved our methodological approach to address the concerns raised by the reviewer with regard to our vulnerability analysis.

1) The description of converting reported flood damages to a single benchmark (L372-378) is not fully clear and suggests that they may not be converted properly to make them comparable with exposure data. The fastest way to resolve this would be for the authors to show an example calculation for a single event: flood in the UK and Germany on 4-7 January 1982 with losses of 700 mln USD in nominal prices (according to NatCatSERVICE).

We thank the reviewer for this comment. Indeed we did not adjust the observed NatCat damages with regard to differences in purchasing power across countries. In the revised manuscript, we follow (Jongman et al., 2015), and ensure that both, observed NatCat damages and the asset data used for the calculator of the modeled damages, are deflated and converted to US dollars at PPP values (base year 2005) to facilitate spatiotemporal comparison. We now detail the conversion procedure in the Section *Socio-economic data sources* of the Methods.

2) Later the authors describe how reported losses were aggregated (L378-384). The text states that "we…assign the damage to the risk area of the centroid closest to the geocode location." What is this risk area? Is it a continuous flood extent, and the risk area corresponding to the event is connected with the nearest patch among the various patches of flood extents? NatCatSERVICE is usually is rather imprecise in defining flood extents, like in the above mentioned example where it is given as "United Kingdom, Germany" (at least in the publicly-available website, but I guess the authors have access also to non-public information?).

Wouldn’t it make more sense to include the flood extents covering the whole ‘political’ area defined in the NatCatSERVICE rather than potentially incorrectly assigning the flood to a small flood extent patch?

We thank the reviewer for this comment. We decided to address it taking also the suggestions of reviewer #3 into account that the comparison of observed and modeled damages should be best done on the level of river basins, because the coordinates of the NatCat database may not be exact. (Indeed we use a non-publicly available version of the database which also includes a single geographical location (longitude and latitude) for each event.).

In the amended version of the manuscript, we first disaggregate the world into river basins following the river basin classification of the Global Runoff Data Centre (GRDC) of the World Meteorological organization (GRDC, 2020). For each basin, we then determine the discharge trends in the historical period separately for each grid cell in the basin. We attribute a positive (negative) discharge trend to the whole basin when the majority of the grid cells have positive (negative) discharge trends. We then divide each of the 9 world regions into subregions R_+ (R_-) subregions aggregating river basins with positive (negative) discharge trends (Figure 1, SI3 and SI4). Attributing damages to river basins instead of individual grid cells as done in the previous version of the manuscript allows us to obtain more robust regression results. In line with the comments of reviewer #3, this makes us confident that river basins are the best spatial unit to compare modeled and observed damages when using NatCatService.

3) Related to the above: the vulnerability estimates in Figs. SI1 and SI2 for developed regions (NAM, WEU, OCE) show vastly higher reported losses compared with observations compared with other parts of the world. I wonder whether the use of FLOPROS flood protection data is the reason here. With a 40-year observation period for discharges there will be not many floods to exceed, say, a 100-year return period. Meanwhile, large parts of NAM and WEU have 100-year or more protection standards according to FLOPROS. Would that narrow down the flood extent and, consequently, exposure too much for the developed countries? This could also explain outliers such 2002 Germany and 2007 UK floods, which had high losses in nominally flood-protected areas. Could the authors show the calculation of vulnerability and modelled spatial extents for the 2002 flood?

We completely agree with the reviewer that we find high vulnerability levels in developed compared to less developed regions. This finding seems to partially disagree with previous studies reporting higher vulnerability levels in less developed countries compared to developed countries. However, we would like to point out that, in contrast to the previous studies, we here compare simulated damages to recorded damages instead of exposed assets to damage record. In consequence, the absolute vulnerability levels of Figs. S1 and S2 cannot be

directly compared between regions since in our simulations, we already account for regional differences in protection levels as given by the FLOPROS database as well as differences in the vulnerability resulting from the region specific debt-damage functions that may result for instance from different housing types. These components of the vulnerability are kept constant throughout the simulations. In consequence, Fig. S1 and Fig. S2 depict only the “transient” component of the vulnerabilities and only changes of these vulnerability components with time should be compared across regions. These trends in vulnerabilities are roughly in line with the vulnerability trends reported in the studies of Jongman et al. (2015) and Tanoue et al. (2016). Though, a direct comparison is difficult because in each of the works different regional aggregations, different time periods, and different methods (e.g, moving averages vs Singular Spectrum Analysis) have been used, the vulnerability trends reported in this study largely align with the earlier studies. For instance, in line with the earlier studies, we find declining vulnerabilities globally and in several less developed regions such as Eastern Asia and Latin America. We would further like to point out that by calculating vulnerabilities on the basis of river basins, the vulnerability analysis has overall become much more robust.

We would agree that due to the high protections standards in developed countries taken from the policy layer of the FLOPROS database, our analysis may underestimate flood extent and flood damage in some cases, for instance when dikes breach in nominally flood protected areas. We therefore repeated our simulations for two additional setups. First, we tested whether the policy layer of the FLOPROS database overestimates flood protection compared to the modeled layer. (While the modeled layer complements missing records for regional protection standards with modeled protection standards based on the regions’ GDP the policy layer uses current protection policies Scussolini et al. (2016). (Our results remain largely robust when choosing the “modeled” instead of the “policy” layer in FLOPROS, especially in developed countries, probably because reporting of protection standards is quite complete in these countries. We now discuss the results obtained for the different FLOPROS layers in the new Section “Limitations and uncertainties” in the Methods. Second, we did the simulations for the limiting case of zero protection levels, which however results in a loss of the year-to-year variability in modeled damages, i.e., one of the main advantages of our modeling approach gets lost. Based on these experiments, we decided that, in line with other recent studies on flood risk (e.g. Dottori et al., 2018), FLOPROS would be the best choice for our approach that aims at obtaining globally consistent flood estimates and at avoiding ad-hoc assumptions such as globally constant protection levels (e.g., 100 years protection) as assumed in previous studies (Hirabayashi et al., 2013; Jongman et al., 2012).

Eventually, to best fit the observed data, we calculate annually averaged vulnerabilities for large world regions based on medians of multi-model damage estimates. Therefore it may be misleading to compare these to the vulnerabilities of single events such as the 2002 Germany or 2007 UK floods.

4) In Fig. S1 it is noticeable that it is largely singular yearly outliers that are responsible of upward vulnerability trend in NAM and WEU. The authors use a

1000:1 ratio for removing outliers, but that is decidedly too high to capture them. Also, computing the trends the authors put together events of vastly different magnitude, which raises mainly the problem of calculating the correct flood footprint to compare with observations: small floods with broadly defined country-level extent will show little vulnerability and big events will show much higher vulnerability. NatCatSERVICE is hardly comprehensive and varies significantly in precision of flood extent definitions as country or sub-country units. Given those limitations, I don’t think it makes sense to use a complex time-varying smoothing method. A simple trend over all data points would improve the confidence in the results somewhat. Otherwise, the authors would need to improve the homogeneity of the vulnerability data. In summation, the authors need to provide better evidence that their vulnerability calculation is robust enough for inclusion in the analysis. This is the main finding of my review. Some other comments of mine are only minor textual suggestions:

We thank the reviewer for this constructive suggestion. We have tested the robustness of our results regarding different choices regarding the vulnerability fits. By design these choices do not at all affect the estimation of the climate induced loss trends which represent the focus of our analysis in contrast to former papers focussing on vulnerability trends. The fit to vulnerability data only indirectly affects our results as it may have an impact on i) the explained variance reached by the full model and ii) the overall trends derived from the full model. Thus, when testing different approaches, we provide a comparison of the explained variances and trends derived from the slightly modified full model now described in the main text (see table below).

Modified full model discussed in the main text: The basin- instead of grid cell-based subdivision of the world regions turned out to already increase the homogeneity of the vulnerability data (cf. Figs. S1 and S2 in SI). Further, we have adjusted the outlier removal when estimating the vulnerability curves: Instead of using a fixed ratio, we now exclude data points that fall into the outer quantiles of the data distribution as detailed in the Methods. In combination these changes suppress influential outliers that would distort the vulnerability trends. For instance, we now find slightly decreasing vulnerability trends in Europe. For the results discussed in the main text, we decided to keep the Singular Spectrum Analysis method to calculate the vulnerabilities instead of using linear trend analysis, as it has been discussed in previous studies that vulnerabilities often have not changed monotonously with time. For instance, Tanoue et al. (2016) observed U-shaped vulnerability curves in many world regions. Therefore, we would consider it preferable to allow for more complex vulnerability developments in the historical fitting period instead enforcing linear trends in each region. The explained variances of the associated full model and trends for the global

assessment are provided in Figure 2 of the main text and included in the table below.

In addition, we have tested moving averages to describe the temporal evolution of vulnerability as also used in Jongman et al. (2015) and Tanoue et al. (2016) and a linear regression (Fig. RL1 and RL2). Both modifications only have minor effects on the explained variances and overall trends and, as mentioned above, do not affect our main conclusion about climate-induced trends at all (numbers for GLB):

	Variance explained by full model	Trend derived from full model
SSA based vulnerability	30.1 %	4.81 %/year
Moving average of vulnerability	32.8 %	4.63%/year
Simple linear trend of vulnerability	25.1 %	4.71%/year

L27-L32: I find the conclusions in the abstract difficult to understand without reading the paper first, as the first sentence seems to contradict the second, leaving the reader confused whether there was a climate change signal detected or not. Consider rewriting for better clarity.

We thank the reviewer for pointing this out. We now provide a more comprehensive abstract, which is easier to understand for the uninformed reader and provides informative insights.

L41: should be “are extreme precipitation events”.

We corrected the sentence as proposed by the reviewer.

L42: break the sentence into two at “, and”

We divided the sentence into two shorter ones following the reviewer’s suggestion.

L48: should be “1980s”

We removed the redundant letters.

L50: change the second “and” to “, with”

The sentence was corrected by replacing the second “and” by “with”.

Fig. 2: I think the time series graphs are not really helpful in the main paper, as the logarithmic scale makes them anyway look very similar. I suggest moving them to the Supplement and leaving only the bar graphs which are one of the principal findings of the paper, while being at present only squeezed on the side. A full-size figure with only the bar graphs, all with the same vertical scale for comparability, would be better here. Alternatively, the figure could be split into two (line graph and bar graph).

We agree with the reviewer that the side panels support major findings of the work. Therefore, we split Fig. 2 into two separate figures, one for the barplot and one for the time-series.

L159: space missing after “R+”

We inserted a space.

L351: the data are “re-aggregated” to 2.5 arcmin resolution. What was the original resolution then? Also, using the apostrophe (‘) to denote arc minutes would be better.

It was re-aggregated from 18arcsec (0.3’) to 2.5’. To explain this, we added the following sentence to the main text, replacing also “arcmin” by the apostrophe:

For the final assessment, we re-aggregate the high resolution flood depth data from 0.3’ to a 2.5’ resolution (~5km x 5km) by retaining the maximum flood depth as well as the flooded area fraction, defined as the fraction of all underlying high resolution grid cells where the flood depth was larger than zero.

L360: please indicate the resolution of the grid.

We added the resolution of the grid (5arcmin).

L389: 150 arcmin resolution? That’s 2.5 degrees. Is that correct?

150arcmin is a typo here, it should be 150arcsec or 2.5 arcmin, we thank the reviewer for making us aware of this mistake.

Eq. 1: what does “R” denote? What is “Cli-R1980” for instance?

It just denotes “region”, in order to clarify that we refer to the time series in one region or subregion. We clarified this throughout the manuscript and used shorter abbreviations.

Eq. 1-4: single letters should be used to denote variables in equations; please rewrite them, using appropriate sub-indices where necessary.

We tried to use single letters, but kept well-known abbreviations, such as GDP, ENSO, AMO. We also clarified the meaning of the sub-indices.

I am looking forward to your revision of the manuscript.
Best regards,

Dominik Paprotny

Reviewer #2 (Remarks to the Author):

Manuscript ID# NCOMMS-20-24884-T

Journal: Nature Communications (ISSN: 2041-1723)

Title: Climate signals in river flood damages emerge under sound regional disaggregation

Dear Authors,

The submitted manuscript presents trends (if any) in flood damages at regional/sub-regional scales and their associations with atmospheric/oceanic teleconnections conditioned on socio-economic exposure dynamics.

While there is a dearth of literature that focuses on incorporating multiple data sources (i.e., observational and model-based inputs) for local-to-regional scales of flood damages (risk) assessment across the world, I see a great piece of contribution to the field throughout this work. The readers of Nature Communications with varying backgrounds will benefit to a great extent as this work brings into front the matter of flood damage assessment through the exhibition of a homogenous framework by incorporating exposure, vulnerability, economic information, and climate contributions.

We thank the reviewer for the positive evaluation of the novelty of our work and that she/he considers it to be from broad interest.

However, I would recommend that the Authors should be more cautious when trying to explain the observed trends and their relationships with climate/exposure/vulnerability information. Most of the discussions are centered around a relatively low-to-moderate level of explaining the variance (R2 of 30% or higher) or a 90% significance level (with linear formulation of damages against the teleconnection indices and an evident significant degree of uncertainty). At the same time, I am completely aware that a low-to-moderate level of R2 for such analysis at a global scale might be sufficiently reasonable. Still, different sources of uncertainties exist when the spatial scale shifts to a region/sub-region scale. I should also underline here that I appreciate the Authors to report some of the critical issues related to the employed flood damages information (e.g., L102–

104, L225–232). All that said, this is a well-structured manuscript with a clear storyline and informative illustrations.

We thank the reviewer for this constructive feedback. We managed to substantially improve the robustness of our regression analysis for the subregions with homogenous discharge trends by aggregating the sub-regions on the basis of river basins instead of individual grid cells as also suggested by reviewer #3. Further, we increased the robustness of the outlier removal before fitting the vulnerabilities and added several robustness checks to the analysis singling out the main predictors to the climate induced trends that we will discuss in the point-to-point rebuttal below. However, as also acknowledged by the reviewer, we have to cope with mostly moderate levels of explained vulnerabilities due to the global nature of our study.

I am providing a list of major and minor concerns that hopefully will elevate the current scientific soundness and consistency level of this manuscript, as follows:

Major Comments:

I. Unexplained climate-driven or socio-economic driven forcing manifested in flood damages: I was not able to find a concise (and complete) discussions related to this fact the how much variability in each region/sub-region was not able to be captured by either of climate or socio-economic inputs. For instance, how significant was the trend in the remaining residuals in Fig. 4? Did the time-series in each sub-region examine for auto-correlation? Was there a significant trend observed in the residuals of those regions with significant predictors as well? What will be the justifications to explain the negative/positive sign of alpha (Eq. 3–4) for different teleconnections, or proposing a linear model of drivers (without interaction term(s), lag, or higher order of drivers, or embedded information in GMT)? Please add more pieces of information in this regard to the manuscript (Methods or Fig. 4 discussions and/or last paragraph of the Conclusions section).

We thank the reviewer for raising these clarifying questions and constructive suggestions which helped us to improve the presentation of our results and to render the analysis of the climate-induced trend more comprehensive and robust.

Explained variability. We have added a table listing the explained variances after accounting for a) changes in hazard b) changes in hazard and exposure and c) changes in hazard, exposure, and vulnerability for each region and subregion (Table SI2). In addition, we summarize the conclusions from the table in the first part of the results, which now reads:

'In most regions and subregions accounting for climate-induced variability and trends is key for reproducing observed damages. In most cases, the explained variance only gets slightly improved by additionally considering changes in either only exposure or both exposure and variability (Table SI2) with the exception of SEA_{+}, $OCE$and OCE_{-} where explained variance increases strongly, and EAS_{-} and LAM_{-} where it decreases.'

In addition, we now discuss the limitations of our approach in the new sections "Limitations and Uncertainties" of the Methods.

Autocorrelation of climate driven damage time series. In the revised version of the manuscript, we now test all regional and subregional damage time series for autocorrelation applying an ACF based on a full convolution. However, in none of the regions we find relevant autocorrelations, which we discuss in the Methods.

Residual trends after adjusting for climate oscillations and GMT changes or AMO. We now provide a table listing the explained variance that is captured by the best model and the corresponding residual trends in the SI (Table SI5). As soon as AMO and GMT are included in the model, in none of the subregions a significant residual trend remains for the best model.

Signs of coefficients in the model accounting for climate oscillations and changes in global warming. We added the following paragraph to the result section explaining the meaning of the sign of the coefficients of GMT change or AMO, respectively:

The sign of the coefficient of GMT or AMO are always in line with the underlying long-term trend in the damage time series, in the sense that a positive trend in observed damages is associated with a positive coefficient of GMT or AMO, respectively and vice versa for negative trends.As the predictors have been normalized to all vary from 0 to 1 in the considered period the magnitude of the coefficients also inform about the strength of the influence of the individual predictors.

We also further deepened the discussion on the choice of our regression approach in the Methods.

Inclusion of time lags. Our regression approach is based on previous publications on the role of large scale oscillations (Najibi and Devineni, 2018) and global mean temperature (GMT) as predictors of damage variations (Armal et al., 2018). Initially, we did not include lag times in our analysis, because in the literature lags are mostly used when analysing intra-seasonal variability e.g. Guimarães Nobre et al. (2020) and we use of annual data. However, following the suggestion by the reviewer, we now allow for lags for the short-term and decadal oscillations ENSO, PDO and NAO, to allow for the possibility that predictors in the previous year may influence damages in the current year due to seasonal lags. We do not consider time lags in the GMT and AMO responses as they basically represent increasing trends.
The modification increases the estimated relevance of ENSO in line with (Ward

et al., 2014). However, it does not affect our main conclusion that even accounting for variations in ENSO, PDO and NAO there remains a monotonous trend in damages that is captured by either the increase in GMT or AMO.

II. Conditioning on upward/downward trend in discharge: To my understanding, most of the analysis and attributions are performed conditioned on observing a pattern (if any) in discharge over a region/sub-region (Fig. 3?). What do the Authors think for the places such as EAS where both R+ and R- trigger the damages, while the exposure inevitably has increased, and thus the climate shows a significant contribution? I acknowledge that you have considered two fixed exposures (1980 and 2010) to get the relative metric, still how you will explain or quantify the contribution from the changes in climate between these two periods? I presume it will provide more insights to answer this question such that how different was the exposure (or vulnerability) between these two periods per region/sub-region on average?

We thank the reviewer for the constructive comments which provided us with good guidance for revising the corresponding section of the manuscript. Indeed, we define the subregions as regions with homogeneous (positive or negative) discharge trends in the historical period. In the initial version of the manuscript, we aggregated the subregions from the grid cell level. Following the suggestion by Rev. 3, we now aggregate across river basins with positive or negative median discharge trends (see modified methods section).

The reason for classifying subregions with regard to positive/negative trends in annual maximum discharge is the assumption that annual maximum discharge is a good proxy for flood hazards and associated damages. Then the applied separation should allow for the detection of individual positive/negative trends in damages that may cancel out without separation.

The identification of positive / negative climate induced trends in damages in subregions of positive / negative trends in annual maximum discharge basically supports the assumption that the latter is a good proxy for trends in flood hazards. The positive climate induced trend observed damage in SSA- represents an exception form the general rule and suggests that, in these regions, annual maximum discharge may not be an adequate proxy for flood hazards. Thus, one can imagine a situation where median annual maximum discharges decrease but at the same time positive discharge extremes become more pronounced (Fig. RL3). In this situation, the region is subject to a negative trend in annual maximum discharge but rising damage trends. Indeed, there is good empirical evidence that

the risk of high end floods has increased in the last decades in SSA (Fig. 3 and Tab. SI3).

Differences in climate induced trends in damages assuming 1980 and 2010 exposure. In general the impact of climate change of climate change against a non-stationary baseline with temporally varying exposure and vulnerability could be estimated by comparing observed damages trends against damages in a simulated counterfactual world where exposure and vulnerability vary according to observations but climate is assumed to be stationary. This is in fact the definition of 'impact attribution' in Chapter 18 of the IPCC AR5 (Cramer et al., 2014). However, as such an attribution scenario set-up has only recently been added to the ISIMIP3a protocol and associated model simulations are not yet available, we decided for the alternative quantification of climate induced damages where climate induced trends are separated from socio-economic changes by keeping constant socio-economic drivers. This differentiation and the linkage is now explicitly mentioned in the text:

'The calculation of the trends for fixed 2010 exposure further allows for the quantification of the contribution of climate change from 1980-2010 (or 1971 - 2010) to median damages in 2010 as difference between the start level of the regression line and its end level (Tab. SI3). This is closest to the definition of 'climate impact attribution' as defined in Ch18 of the WGII contribution to the IPCC AR534 which would require the comparison of observed damages to damages in a counterfactual situation assuming observed socio-economic conditions but a stationary climate.'

We now also explain the potential differences between climate-induced trends when either fixing 1980 or 2010 socio-economic conditions. Both estimates can be considered legitimate estimates of climate-induced trends in damages where the differences have to be mentioned explicitly, as now done in the text:

'Differences in these two trends arise when there are assets in 2010 in areas where no assets at all were exposed in 1980. Assuming 2010-fixed-exposure all damages that were caused on these assets contribute to the climate-induced trend, while changes in damages on these assets in the 1980-fixed-exposure are attributed to the exposure trends. However, differences between both estimates of 'climate induced damage trends' are found to be minor (Fig. 4).'

Minor Comments:

1. L47: “1980s”

2. L94–95: Please define “all three drivers” here. I acknowledge that you have indicated them in the caption of Fig. 2 and the next lines in this section. Still, it will be better to mention those three drivers when you specifically start to discuss them in the text.

We thank the reviewer for making us aware of this incomprehensive section and followed the suggestion of mentioning the drivers explicitly again.

3. L102–104: I would suggest moving these lines to the corresponding Methods section(s) to avoid any interruption within the flow of contents.

We have added this part fragment of the uncertainty discussion to the extended discussion of Model Limitations and Uncertainties in the Methods.

4. L178: Is it possible to move the panel for Global (and the legend) to somewhere at the top of this figure? In that case, it will be more consistent with Fig. 2, and the reader will be more comfortable.

We thank the reviewer for the comment and amended Fig. 4 accordingly.

5. L237–238: How many events were available in the observed database for individual sub-regions?

We now provide a table in the SIthe exact numbers of events reported in each region/subregion (Table SI6), and refer to this Table in the Methods

6. L288–290: Although we should acknowledge that the observational/model outputs feature a low-to-moderate level of quality across these regions.

We now mention this limitation in the corresponding paragraph and discuss it in the Conclusions.

7. L313–314: Is there any information regarding the sample size or its range over each region/sub-region? While I am aware of the spatial resolution (in different arcmin scales) that you have mentioned in this section, it will make more sense to have such information in numbers/ranges per region/sub-region.

We would completely agree with the reviewer and have added a table with

summary statistics for the observed and modeled damages in each region or subregion to the SI (Table SI6).

8. L359 and L361 and similar places throughout the manuscript: The word “data” should be used plural: “Gridded GDP data were obtained…”, “Downscaled GDP data are available…” and so on.

We corrected this throughout the manuscript.

9. L381: Is that based on a calendar year?

Throughout the manuscript, we refer to calendar years. We added this information in the text.

10. L382: What does “geocodes” mean here? Are they different from the longitudinal/latitudinal (geographic) coordinates?

It refers to geographic coordinates. We changed this throughout the manuscript.

11. L389: I would suggest providing the corresponding scale in ‘km-by-km’ here and elsewhere when you mention ‘arcmins’ as well.

We would agree and now provide the resolution in km-by-km.

12. L404: Please remove the repeated “the” here.

We changed this accordingly.

13. L406: Is there a possibility to provide the exact sample size from the MunichRe database?

We now provide the summary statistics for each subregion in a Table in the SI (Table SI6).

14. L418–419: How many missing values were there?

In entire regions, we find a low level of missing data, with a maximum of 4 in Central Asia, mostly likely due to an underreporting before 1990 leading to zero vulnerabilities. In some subregions, data coverage is somewhat worse, especially in OCE-, where we find 17 missing values and NAF+ where we find 8 missing values. However, in the subregions included in the trend analysis (R^2 > 20%) there are a maximum of 6 values missing in CAS+ and in OCE-. We added this information to the methods section in the manuscript.

15. L440–443: If you are going to specify the sources of observations for the teleconnections provided by NOAA, could you please double-check the links and

source of observations for the NAO indices? I assume NAO indices are available through the NOAA/CPC (not NOAA/ESRL).

We thank the reviewer for pointing us to this error, we corrected this throughout the manuscript.

16. L444: What are those three input climatic forcings? Please be specific.

In the revised version of the manuscript, we added these information to the Methods.

17. L450: I believe Armal et al. (2018) used (multiple) multilevel regression model, which I would not recommend to cite it here and in this content.

We revised the corresponding paragraph in the manuscript, to make clear that we cite Arman et al. only because they also include GMT as prediction in their analysis in addition to the teleconnections.

18. L463: The residuals must be checked in terms of periodicity (if any) in addition to the remaining trends. A simple ACF can examine this matter if you have not done this testing.

We thank the reviewer for bringing up this point. We have tested all residuals for periodicity applying an ACF basing on a full convolution. In most regions we do not find periodicity, but low values of autocorrelation in the regions in (GLB, GLB_+, AUS, AUS_-, CHN). Additionally, we applied a Hamed and Rao Modified Mann-Kendall Test in these regions, which did not change the statements on significance compared to the previous analysis.

Thank you, -Reviewer

Reviewer #3 (Remarks to the Author):

This study attempts to detect climate signals from the reported economic damage due to river flooding. The risk driver (i.e., economic damage) was decomposed into three factors (hazards, exposure, and vulnerability), then the trend analysis of these factors was used to detect climate signals. I have the challenging impression for this study, the idea is very good and is of interest. However, I have some questions and suggestions about the regional classification, model uncertainty and validation, calculation of time variation of vulnerability. For this

reason, the manuscript can be required revisions before publication. The main points raised are as follows.

We thank the reviewer for considering our work to be of broad interest. We have substantially revised the manuscript and improved our methodological approach to address all concerns raised by the reviewer. For instance, we managed to substantially improve the robustness of our regression analysis for the subregions with homogenous discharge trends by aggregating the sub-regions on the basis of river basins instead of individual grid cells following the reviewer's suggestion as detailed below.

1. In this study, they divided into 18 regions based on the geographical classification and long-term trend in river discharge. Why did not the author classified based on river basin units? This long-term trend in simulated river discharge is calculated based on runoff output derived from multiple GHMs forced by bias corrected meteorological data, however the simulated river discharge have uncertainties derived from complicated model chain (e.g., rainfall patrons, land surface hydrological cycle, and digital elevation model).

We thank the reviewer for the constructive suggestion to classify sub-(regions) based on river basins. We would fully agree that given the current data limitations (e.g., not exact geographic locations of reported damages in the NatCat database), river basin units are the most appropriate unit for our global study. We repeated all calculations based on the basin classification published recently by the Global Runoff Data Centre (GRDC, 2020). Given that for damage assessment country-level socio-economic variables are also important, we did not completely reject our original regional classification based on aggregating countries into 9 world regions. However, instead of choosing subregions on the basis of discharge trend in individual grid cells, we now classify all river basins in the world region according to their dominant discharge trends in the historical period and then aggregate them to the positive and negative discharge subregions (Fig. 1b and Fig. SI4).

This classification based on river basins significantly improved the robustness of our regression analysis in the subregions. We revised the manuscript accordingly and added details of the classification procedure to the Methods.

We would further agree that our GHM based approach to derive past discharge trends is subject to model uncertainties. However, we would like to stress that multi-model assessments have proved to be a valid tool for large-scale long-term changes in discharge trends and are much more robust regarding long-term trends than for prediction of the exact magnitude of specific events (Zaherpour et al., 2018; Hattermann et al., 2017). Thereby, coupling GHMs with a river routing scheme such as CaMa-Flood, which is able to account for floodplain storage capacities and backwater effects, leads to a better agreement with observed discharge than relying only on routing schemes in most GHMs (Zhao et al., 2017). A detailed qualitative comparison of our modeling discharge trends to observed

discharged trends may be found in Sec. 4 of SI. However, on a global level using records of past discharges do not seem to be the better alternative since reporting quality (e.g., record length, distance between stations) strongly differs across the globe (Do et al., 2017; Gudmundsson et al., 2019) which makes it harder to obtain comparable discharge trends on a global level. Further, observed discharge trends also account for river engineering and catchment changes (urbanisation, deforestation...), which makes it more difficult to extract climate related discharge trends than in modeling approaches where we can control for these factors.

2. Furthermore, the author forcibly grouped a grid with unclear long-term trend and low statistical significant (e.g., Sahel region, western part of China etc). In addition, although this study used the event location information recorded in NatCatSERVICE, it is difficult to accurately recorded the location of the event. Flood events occur in river basin unit. Therefore, the used regional classification should be based on river basin unit for more extracting robust climate signals.

We thank the reviewer for pointing us towards the limited accuracy of the event location in the NatCatSERVICE database. In the revised version of the manuscript, we now assign the NatCat damages to the river basin surrounding their event location. Overall, the results in a better agreement of modeled and observed damages, especially on global level and in South & South Eastern Asia (Fig. 2). In the other regions, we observe only little changes in the explained variance (differences in other regions are caused by changes in the vulnerability estimates (see discussion below) and a PPP adjustment of damage records). Significance of climate trends remains largely unchanged.

We are very grateful for the reviewer’s suggestion to choose river basins as the basic unit of our analysis and will also employ this classification in follow-up studies.

3. This study validated the long-term trend of river discharge between the simulation and observation, but does not address other factors (e.g., economic damage). The CaMa-Flood, used in this study, has been validated by numerous studies, however this study used different input dataset (i.e., ISI-MIP2) with other studies, therefore the author should state validation of flood water depth and flood extension. In addition, although CLIMADA is used for the damage estimation, the

author should describe how much uncertainty there is in the calculation of the economic damage.

The paper now include a detailed discussion of potential uncertainties associated with our approach with a focus on potential systematic effects on the estimated climate induced trends in observed damages:

'Limitations and uncertainties.
*The damage modeling applied in this study shows varying quality across regions (Fig.2)**.** The NAM region where the observed damage variability can be almost entirely captured by the modeling chain highlights the general power of the modelling approach. Resolving the reasons for lower explained variances in other regions will be an important next step to improve the proposed approach as a tool for climate impact attribution. Deficits could be introduced along the entire modelling chain ranging from i) observational climate forcing data whose quality varies from region to region, ii) translation of daily climate data into discharge that could be due to inadequate process-understanding or inadequate representation of direct human influences, iii) translation of discharge into flooded areas, iv) derivation of exposed assets and translation into damages, and v) deficits of reported damages. Here we discuss sources of uncertainties step-by-step with a focus on potential biases of the climate induced trends in damages being central for this study:*
i) Climate reanalysis data constrain the performance of GHMs and may partly be responsible for the spatial variation in explanatory power (Beck et al., 2017; Ruane et al., 2015), e.g., due to spatially and temporarily heterogenous global data coverage. However, the good agreement between observed discharge trends and the median discharge trends derived from the model simulations forced by four different observational data indicates that the uncertainties do not lead so large scale systematic biases in trends (SI Sec. 4).
ii) There are several sources of uncertainty associated with the translation of the climate forcing into discharge. They may refer to the exact timing and scaling of peak-runoff (Beck et al., 2017;Zaherpour et al., 2018) and the translation into spatially explicit flood-depth on the basis of Digital Elevation Models that are known to be fraught with high levels of uncertainty (Yamazaki et al., 2011). Spatial variations in GHM performance may explain part of the spatial variation in the correlations between observed and simulated damages. Thus, validation studies of the GHMs included in ISIMIP2a indicate a variation in performance across hydrobelts (Beck et al., 2017; Zaherpour et al., 2018) with a better performance in the wetter equatorial and northern hydrobelts than in drier southern hydrobelts (Zaherpour et al., 2018). This is partly reflected in our results, as we observe very low explanatory power in regions such as North Africa and better explanatory power in Asian and North American regions or subregions. In addition GHMs show a tendency to overestimate peak-runoff (Zhao et al., 2017; Beck et al., 2017). However, accounting for floodplain storage and backwater effects as implemented into CaMaFlood that is also applied in this study leads to a general reduction of peak river discharge and to a better agreement between observed and simulated annual peak discharge(Zhao et al., 2017). In general the uncertainties of hydrological modelling discussed here are not expected to introduce systematic biases in trends, as modeled and observed discharge trends show very similar patterns (SI Sec. 4).

iii) Regarding the exact modeling of flood extents and flooded areas, the river routing model CaMa-flood has been validated in many studies (Bernhofen et al., 2018; Ikeuchi et al., 2015; Mateo et al., 2017), but not yet within the ISIMIP2a context. Previous studies on selected river basins in Nigeria and Mozambique revealed performance differences ranging from poor to good across river basins and a strong dependence on the return period of input flows (Bernhofen et al., 2018). The representation of flooded areas may particularly depend on the adequate representation of flood protection levels only coarsely known and implemented based on FLOPROS in this study. We quantify the sensitivity of damages using two different data versions, the merged-layer (Figs. 2-4) and the modeled-layer of FLOPROS (Figs. SI7 and SI8). Overall, our main findings are robust (explained variances and significance, sign and amplitude of climate-induced trends) with regard to the choice of the FLOPROS layer. Compared to the merged layer, we find slightly higher explained variances in several less developed regions such as LAM and SEA but slightly lower explained variances in NAM when using the modeled-layer of FLOPROS (cf. Fig. 2 to Fig. SI7). These differences are most likely rooted in an overestimation of policy standards in less developed areas that used as fill-values for missing data on implemented protection standards in the merged layer. The sensitivity does not address potential biases that could be induced by temporally changes in protection levels not captured by FLOPROS only providing a snapshot. Instead of the explicit modelling through protection levels this effect is captured by the temporal variation in the derived vulnerability factors.

iv) In this study, we observe a tendency of overestimating damages in low-income areas (e.g., SSA) and of underestimating damages in high-income regions (e.g., OCE) (Figs. SI1 and SI2). The major reasons here are the continental depth-damage functions that assume less vulnerability in high-income countries (Huizinga et al., 2017) and the protection standards in the FLOPROS database that are much higher in developed countries. However, it is important to note that these only affect the quantification of the vulnerability trends and do not affect the significance, directions, and magnitude of the climate-induced trends.

v) Finally, the reported damages from the NatCatSERVICE database cannot be assumed to be free of biases. However, a likely underreporting in early years will not introduce systematic biases in the estimation of climate-induced trends, but may affect the time-variable vulnerability estimate.

It is expected that it will become possible to explicitly address iii) and v) when extending the analysis to include the last decade. Thus v) could be addressed by checking whether the correlation between observed and simulated damages increases with time indicating that discrepancies found here are reduced by improved reporting of damages. In addition, simulations of flooded areas could be evaluated based on high-quality satellite data becoming available for 1985 onwards.
Our analysis on the relative importance of different teleconnections and GMT may be limited by the fact that climate oscillations are not completely uncorrelated from changes in GMT, i.e., our predictors cannot be assumed to be completely independent. Therefore, we caution in the main text that the selection of GMT as a relevant driver does not necessarily rule out the additional relevance of climate oscillations.'

We acknowledge that an appropriate validation of the performance of CaMa-Flood for the GHMs participating in ISIMIP2a would be beneficial for this study. However, we would like to point out that, as validation studies on flood extends remain limited to selected single basins, it would be difficult to draw conclusions on the overall global validity of the CaMa-Flood output that is used in this study. We currently work on a detailed validation study that quantifies the capacity of CaMa-Flood driven by ISIMIP2a data to reproduce flood extents of major past flood events in different river basins, also pointing towards varying performance between river basins.

4. Previous studies (e.g., Jongman et al., 2015; Tanoue et al., 2016) has shown that long-term variation in vulnerability decreases with income growth and over time. However, this study ignores this effect and calculates the vulnerability on the regional classification. If the vulnerability variation in Fig. S1 is calculated as the sum of reported and modelled economic damage, the vulnerability will be strongly influenced by countries with higher income. Therefore, vulnerability should interpolate for each year and each country, and then these aggregated. Year-national scale interpolation method of vulnerabilities have been proposed by Kinoshita et al (2018) and Dottori et al (2018).

• Dottori, F. et al. Increased human and economic losses from river flooding with anthropogenic warming. Nature Climate Change 8, 781-786, doi:10.1038/s41558-018-0257-z (2018).

• Kinoshita, Y., Tanoue, M., Watanabe, S. & Hirabayashi, Y. Quantifying the effect of autonomous adaptation to global river flood projections: application to future flood risk assessments. Environmental Research Letters 13, doi:10.1088/1748-9326/aa9401 (2018).

• Jongman, B. et al. Declining vulnerability to river floods and the global benefits of adaptation. Proc Natl Acad Sci U S A 112, E2271-2280, doi:10.1073/pnas.1414439112 (2015).

• Tanoue, M., Hirabayashi, Y. & Ikeuchi, H. Global-scale river flood vulnerability in the last 50 years. Sci Rep 6, 36021, doi:10.1038/srep36021 (2016).

It is important to note that our study does not aim for providing vulnerability estimates as e.g. area weighted average of individual vulnerability across all exposed assets. In our case vulnerability can be considered *defined* as the ratio of regionally aggregated observed damages to simulated damages and designed to reproduce observed damages. The calculated ratios is expected to emerge from potentially highly variable individual vulnerabilities of assets e.g. in terms of income variations within the large scale regions. We now highlight that aspect

clearly in the main text.

'It is important to note that the applied definition of regional vulnerability as the ratio of regionally aggregated observed damages and associated simulated damages cannot be considered a spatial average of individual vulnerabilities of assets that may be subject to strong variations according to income levels (Jongman et al., 2015)*. As the aggregated simulated exposed assets may be dominated by highly valuable assets in high income regions the vulnerability factor derived here is also expected be strongly influenced by associated individual vulnerabilities.'*

We are aware and cite the previous studies in our work that show vulnerabilities to decrease with income. We do not neglect this dependence in our study but choose a different approach to estimate vulnerabilities which we would consider better suited for the main purpose of this paper. In our analysis, we focus on the detection and quantification of climate induced trends in economic damages and we aim to determine the underlying drivers of these trends (e.g., large scale oscillations and increases in global mean temperature (GMT)). In contrast, the previous studies focused on gaining a better understanding of the underlying drivers of changes in vulnerabilities. The dependence of vulnerability trends on capita GDP and time was then employed by Kinoshita et al. (2018) to project future vulnerability changes along the Shared Socioeconomic Pathways. For our aim, our method has the advantage to be more flexible; it allows different kinds of vulnerability changes, instead of "forcing a linear trend" or assuming a dependence of any predictor. This allows for better vulnerability estimates in regions where the above relationship is not valid. For instance, Tanoue et al. (2016) show that there are regions with U-shaped vulnerabilities.

We would agree that our method to calculate the vulnerability in each region and year by dividing aggregated observed by aggregate modeled losses attributes a higher weight to events in wealthy regions. However, we think that this is a general problem when analysing economic damages and not a vulnerability specific issue, but rather a question of finding representative impact indicators. Also in recorded damages devastating flood events cause higher nominal damages in wealthy countries than in poor countries.
To minimize this bias we now adjust both, recorded damages from the NatCatSERVICE database as well as modeled damages to 2005 PPP levels (similar to Jongman et al. (2015)). This partially removes the bias towards wealthy countries. We want to highlight that calculating vulnerability on the regional level, in first place, attributes a higher weight to bigger countries or countries which are more affected and are more populated, which we consider a desirable effect, seeing that we want to provide the best empirical estimate for the entire region. Further, summing up damages for the entire region before calculating vulnerability is indispensable for the comparison of the vulnerability changes with other drivers, as also exposure and climate trends will be dominated by bigger and more populated countries. We are aware that we thereby reduce effects of vulnerability changes in small and less affected countries, but think that for a reasonable geographical comparison of the different drivers it is even essential to accept this bias to provide a consistent assessment of the three drivers.

We acknowledge that our analysis in the initial submission was partly outlier driven.In order to achieve more robust vulnerabilities we applied a consistent outlier removal by eliminating data points exceeding a distinct distance to the inner quantile:

$$Q_{0.3} - [5 \cdot (Q_{0.7} - Q_{0.3})] < \frac{D_{obs}}{D_{CliExp}} < Q_{0.7} + [5 \cdot (Q_{0.7} - Q_{0.3})]$$

Minor remarks:

1. It is unclear how the estimated vulnerability is used for calculation of economic damage (e.g., Dcliexp).

We thank the reviewer for pointing this out, so that we were able to clarify this in the methods section. We have inserted a clear description, how the full model is derived from D_{CliExp} using the vulnerability function derived from the ratios $\frac{D_{obs}}{D_{CliExp}}$. The function basically provides a factor for each year that we multiply with D_{CliExp} in order to account for changes in variable climate and exposure modeling.

2. The vulnerability is calculated as a ratio of the reported economic damage to the modeled economic damage. I seem to be seeing some extremely high values (e.g., WEU- and OCE-), this may be because of CPI fixer. To reduce this uncertainty, I recommend the author that estimated and reported nominal values are used for the calculation of vulnerability.

We completely agree with the reviewer that in the submitted version of the manuscript there were some very high vulnerability values, especially in developed regions. First of all, we would like to point out that by repeating our analysis on “river basin units” as suggested by the reviewer, we now find substantially lower values for the fitted vulnerabilities in developed regions and we find a much smaller spread among regional vulnerability values, i.e., less outliers.

However, the observation that vulnerabilities are generally higher in developed than in developing regions remains valid. At the first glance, this finding is apparently in disagreement with several previous studies reporting higher vulnerabilities in developing countries (Jongman et al., 2015; Kinoshita et al., 2018; Tanoue et al., 2016). In these studies, vulnerabilities were estimated as the ratio of observed damages to exposed assets. In contrast, in this study we calculate the vulnerability as the ratio between observed and modeled damages.

Our simulated damages already accounts for regional differences in "static" vulnerabilities as provided by the empirical flood-damage functions and

the FLOPROS protection standards, i.e., "static" vulnerabilities are usually lower in developed regions. These "static" component of the vulnerability remain constant throughout the simulations. In consequence, Fig. S1 and Fig. S2 depict only the "transient" components of the vulnerability. Thus, in contrast to previous studies these vulnerabilities cannot be directly compared across regions, and only changes of these vulnerabilities over time should be compared across regions. These trends in the "transient"vulnerabilities are roughly in line with the vulnerability trends reported in the studies of Jongman et al. (2015) and Tanoue et al. (2016). Though, a direct comparison is difficult because in each of the works different regional aggregations, different time periods, and different methods (e.g, moving averages vs Singular Spectrum Analysis) have been used, the vulnerability trends reported in this study largely align with the earlier studies. For instance, in line with the earlier studies, we find declining vulnerabilities globally and in several less developed regions such as Eastern Asia and Latin America.

In the revised manuscript, we follow Jongman et al. (2015)and adjust both, observed damages and exposed assets, with regard to inflation and to differences in purchasing power and provide both quantities in inflation adjusted 2005 purchasing power parity (PPP) USD. We do not think that extreme values of the vulnerability in the first version of the manuscript resulted from jumps in the consumer price index but rather from the inexact NatCat event locations in combination with the aggregation of subregions from individual grid cells, an issue which is now solved by using "river basin units" following the suggestion by the reviewer.

3. The FLOPROS Merged Layer may be too high flood protection level for low-income countries. For example, in Bangladesh, the Policy Layer (i.e., future target protection level) is applied for all over the country. Its value is over 50 years, which is very higher than the Model Layer estimated by income level. For this reason, recent some studies (e.g., Ward et al., 2017) have used the Model Layer of FLOPROS.

• Ward, P. J. et al. A global framework for future costs and benefits of river-flood protection in urban areas. Nature Climate Change 7, 642-646, doi:10.1038/nclimate3350 (2017).

We thank the reviewer for this constructive comment. We repeated our analysis using the modeled layer of FLOPROS, and find indeed higher explained variances in Latin America and South-& South East Asia. However, we decided

to show the results as obtained for the merged layer of FLOPROS in the main text since we find in most regions a better agreement with the observations. However, we now show the results as obtained for the modeled layer in the SI (Fig. SI7 and SI8), and added a discussion on the uncertainties introduced by FLOPROS in the Section on limitations and uncertainties in the Methods.

4. How did you convert the river discharge to flood water depth? CaMa-Flood diagnoses the river discharge based on the amount of total water storage including downstream cells, therefore the river discharge cannot be directly converted to flood water depth. In addition, there are sometimes negative values found in simulated river discharge in CaMa-Flood, I wonder whether the GEV parameters have been properly calculated using these values. Therefore, it may be better to estimate the return period using other variables (e.g., total water storage and flood water depth etc).

We would completely agree with the reviewer that that river discharge cannot directly be converted to flood water depth. Thus, we here rely on a look up table based on a specific MATSIRO run, which takes observations as a driver into account. We thereby rely on the method as in Hirabayashi et al. (2013), who also argue that, though not perfect, discharge as output by CaMa-Flood is the best basis for return period estimation. In the distribution fit we take negative values into account (as also these distributions by definition have a positive probability for negative values).

5. The axis of the bar chart on the right of Fig. 2 is barely visible.

We divided Fig. 2 in two plots to achieve better visualization of some key messages. We introduced a clearly labeled legend with colors, instead of labeling the axis.

References

Armal, S., Devineni, N., Khanbilvardi, R., 2018. Trends in Extreme Rainfall Frequency in the Contiguous United States: Attribution to Climate Change and Climate Variability Modes. J. Clim. 31, 369–385. https://doi.org/10.1175/JCLI-D-17-0106.1

Beck, H.E., van Dijk, A.I.J.M., de Roo, A., Dutra, E., Fink, G., Orth, R., Schellekens, J., 2017. Global evaluation of runoff from 10 state-of-the-art hydrological models. Hydrol. Earth Syst. Sci. 21, 2881–2903. https://doi.org/10.5194/hess-21-2881-2017

Bernhofen, M.V., Whyman, C., Trigg, M.A., Sleigh, P.A., Smith, A.M., Sampson, C.C., Yamazaki, D., Ward, P.J., Rudari, R., Pappenberger, F., Dottori, F., Salamon, P., Winsemius, H.C., 2018. A first collective validation of global fluvial flood models for major floods in Nigeria and Mozambique. Environ.

Res. Lett. 13, 104007. https://doi.org/10.1088/1748-9326/aae014

Cramer, W., Yohe, G.W., Auffhammer, M., Huggel, C., Molau, U., Dias, M. a. F.S., Leemans, R., 2014. Detection and attribution of observed impacts. Clim. Clim. Change 2014 Impacts Adapt. Vulnerability 979–1038. https://doi.org/10.1017/CBO9781107415379.023

Do, H.X., Westra, S., Leonard, M., 2017. A global-scale investigation of trends in annual maximum streamflow. J. Hydrol. 552, 28–43. https://doi.org/10.1016/j.jhydrol.2017.06.015

Dottori, F., Szewczyk, W., Ciscar, J.-C., Zhao, F., Alfieri, L., Hirabayashi, Y., Bianchi, A., Mongelli, I., Frieler, K., Betts, R.A., Feyen, L., 2018. Increased human and economic losses from river flooding with anthropogenic warming. Nat. Clim. Change 8, 781–786. https://doi.org/10.1038/s41558-018-0257-z

GRDC, 2020. BfG - Geospatial Data Products - WMO Basins and Sub-Basins, 3rd ed. (GRDC, 2020) [WWW Document]. URL https://www.bafg.de/GRDC/EN/02_srvcs/22_gslrs/223_WMO/wmo_regions_2020.html?nn=201570 (accessed 10.26.20).

Gudmundsson, L., Leonard, M., Do, H.X., Westra, S., Seneviratne, S.I., 2019. Observed Trends in Global Indicators of Mean and Extreme Streamflow. Geophys. Res. Lett. 46, 756–766. https://doi.org/10.1029/2018GL079725

Guimarães Nobre, G., Moel, H. de, Giuliani, M., Bischiniotis, K., Aerts, J.C.J.H., Ward, P.J., 2020. What Will the Weather Do? Forecasting Flood Losses Based on Oscillation Indices. Earths Future 8, e2019EF001450. https://doi.org/10.1029/2019EF001450

Hattermann, F.F., Krysanova, V., Gosling, S.N., Dankers, R., Daggupati, P., Donnelly, C., Flörke, M., Huang, S., Motovilov, Y., Buda, S., Yang, T., Müller, C., Leng, G., Tang, Q., Portmann, F.T., Hagemann, S., Gerten, D., Wada, Y., Masaki, Y., Alemayehu, T., Satoh, Y., Samaniego, L., 2017. Cross- scale intercomparison of climate change impacts simulated by regional and global hydrological models in eleven large river basins. Clim. Change 141, 561–576. https://doi.org/10.1007/s10584-016-1829-4

Hirabayashi, Y., Mahendran, R., Koirala, S., Konoshima, L., Yamazaki, D., Watanabe, S., Kim, H., Kanae, S., 2013. Global flood risk under climate change. Nat. Clim. Change 3, 816–821. https://doi.org/10.1038/nclimate1911

Huizinga, J., De Moel, H., Szewczyk, W., 2017. Global flood depth-damage functions: Methodology and the database with guidelines (EUR - Scientific and Technical Research Reports). Publications Office of the European Union.

Ikeuchi, H., Hirabayashi, Y., Yamazaki, D., Kiguchi, M., Koirala, S., Nagano, T., Kotera, A., Kanae, S., 2015. Modeling complex flow dynamics of fluvial

floods exacerbated by sea level rise in the Ganges–Brahmaputra–Meghna Delta. Environ. Res. Lett. 10, 124011. https://doi.org/10.1088/1748-9326/10/12/124011

Jongman, B., Ward, P.J., Aerts, J.C.J.H., 2012. Global exposure to river and coastal flooding: Long term trends and changes. Glob. Environ. Change 22, 823–835. https://doi.org/10.1016/j.gloenvcha.2012.07.004

Jongman, B., Winsemius, H.C., Aerts, J.C.J.H., Perez, E.C. de, Aalst, M.K. van, Kron, W., Ward, P.J., 2015. Declining vulnerability to river floods and the global benefits of adaptation. Proc. Natl. Acad. Sci. 112, E2271–E2280. https://doi.org/10.1073/pnas.1414439112

Kinoshita, Y., Tanoue, M., Watanabe, S., Hirabayashi, Y., 2018. Quantifying the effect of autonomous adaptation to global river flood projections: application to future flood risk assessments. Environ. Res. Lett. 13, 014006. https://doi.org/10.1088/1748-9326/aa9401

Mateo, C.M.R., Yamazaki, D., Kim, H., Champathong, A., Vaze, J., Oki, T., 2017. Impacts of spatial resolution and representation of flow connectivity on large-scale simulation of floods. Hydrol. Earth Syst. Sci. 21, 5143–5163. https://doi.org/10.5194/hess-21-5143-2017

Najibi, N., Devineni, N., 2018. Recent trends in the frequency and duration of global floods. Earth Syst. Dyn. 9, 757–783. https://doi.org/10.5194/esd-9-757-2018

Ruane, A.C., Goldberg, R., Chryssanthacopoulos, J., 2015. Climate forcing datasets for agricultural modeling: Merged products for gap-filling and historical climate series estimation. Agric. For. Meteorol. 200, 233–248. https://doi.org/10.1016/j.agrformet.2014.09.016

Scussolini, P., Aerts, J.C.J.H., Jongman, B., Bouwer, L.M., Winsemius, H.C., de Moel, H., Ward, P.J., 2016. FLOPROS: an evolving global database of flood protection standards. Nat. Hazards Earth Syst. Sci. 16, 1049–1061. https://doi.org/10.5194/nhess-16-1049-2016

Tanoue, M., Hirabayashi, Y., Ikeuchi, H., 2016. Global-scale river flood vulnerability in the last 50 years. Sci. Rep. 6, 36021. https://doi.org/10.1038/srep36021

Ward, P.J., Jongman, B., Kummu, M., Dettinger, M.D., Weiland, F.C.S., Winsemius, H.C., 2014. Strong influence of El Niño Southern Oscillation on flood risk around the world. Proc. Natl. Acad. Sci. 111, 15659–15664. https://doi.org/10.1073/pnas.1409822111

Yamazaki, D., Kanae, S., Kim, H., Oki, T., 2011. A physically based description of floodplain inundation dynamics in a global river routing model. Water Resour. Res. 47. https://doi.org/10.1029/2010WR009726

Zaherpour, J., Gosling, S.N., Mount, N., Schmied, H.M., Veldkamp, T.I.E., Dankers, R., Eisner, S., Gerten, D., Gudmundsson, L., Haddeland, I.,

Hanasaki, N., Kim, H., Leng, G., Liu, J., Masaki, Y., Oki, T., Pokhrel, Y., Satoh, Y., Schewe, J., Wada, Y., 2018. Worldwide evaluation of mean and extreme runoff from six global-scale hydrological models that account for human impacts. Environ. Res. Lett. 13, 065015. https://doi.org/10.1088/1748-9326/aac547

Zhao, F., Veldkamp, T.I.E., Frieler, K., Schewe, J., Ostberg, S., Willner, S., Schauberger, B., Gosling, S.N., Schmied, H.M., Portmann, F.T., Leng, G., Huang, M., Liu, X., Tang, Q., Hanasaki, N., Biemans, H., Gerten, D., Satoh, Y., Pokhrel, Y., Stacke, T., Ciais, P., Chang, J., Ducharne, A., Guimberteau, M., Wada, Y., Kim, H., Yamazaki, D., 2017. The critical role of the routing scheme in simulating peak river discharge in global hydrological models. Environ. Res. Lett. 12, 075003. https://doi.org/10.1088/1748-9326/aa7250

Figure RL1: Vulnerability estimates comparing SSA and an 11-year-running mean.

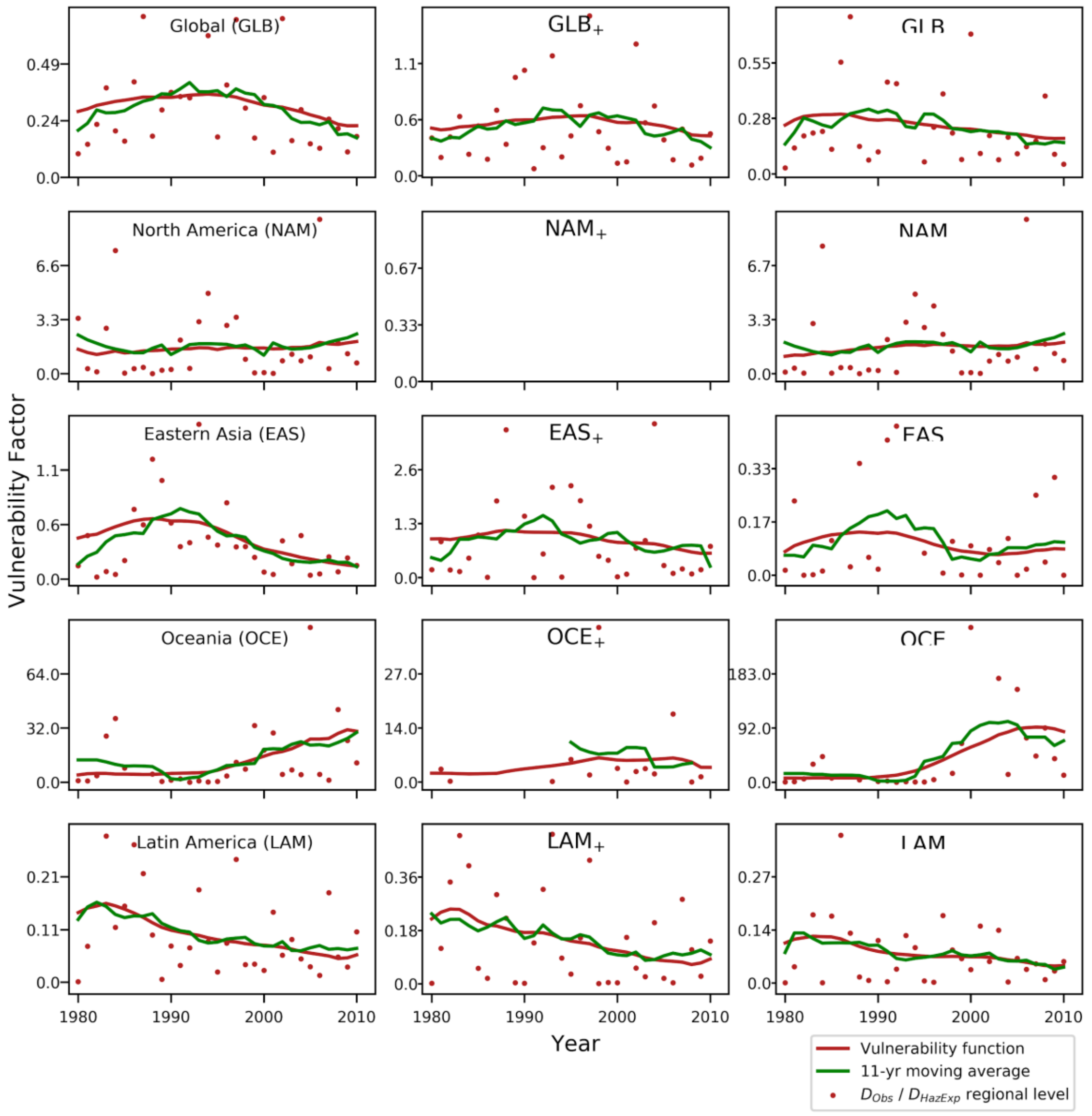

Figure RL2: Vulnerability estimates comparing SSA and an 11-year-running mean.

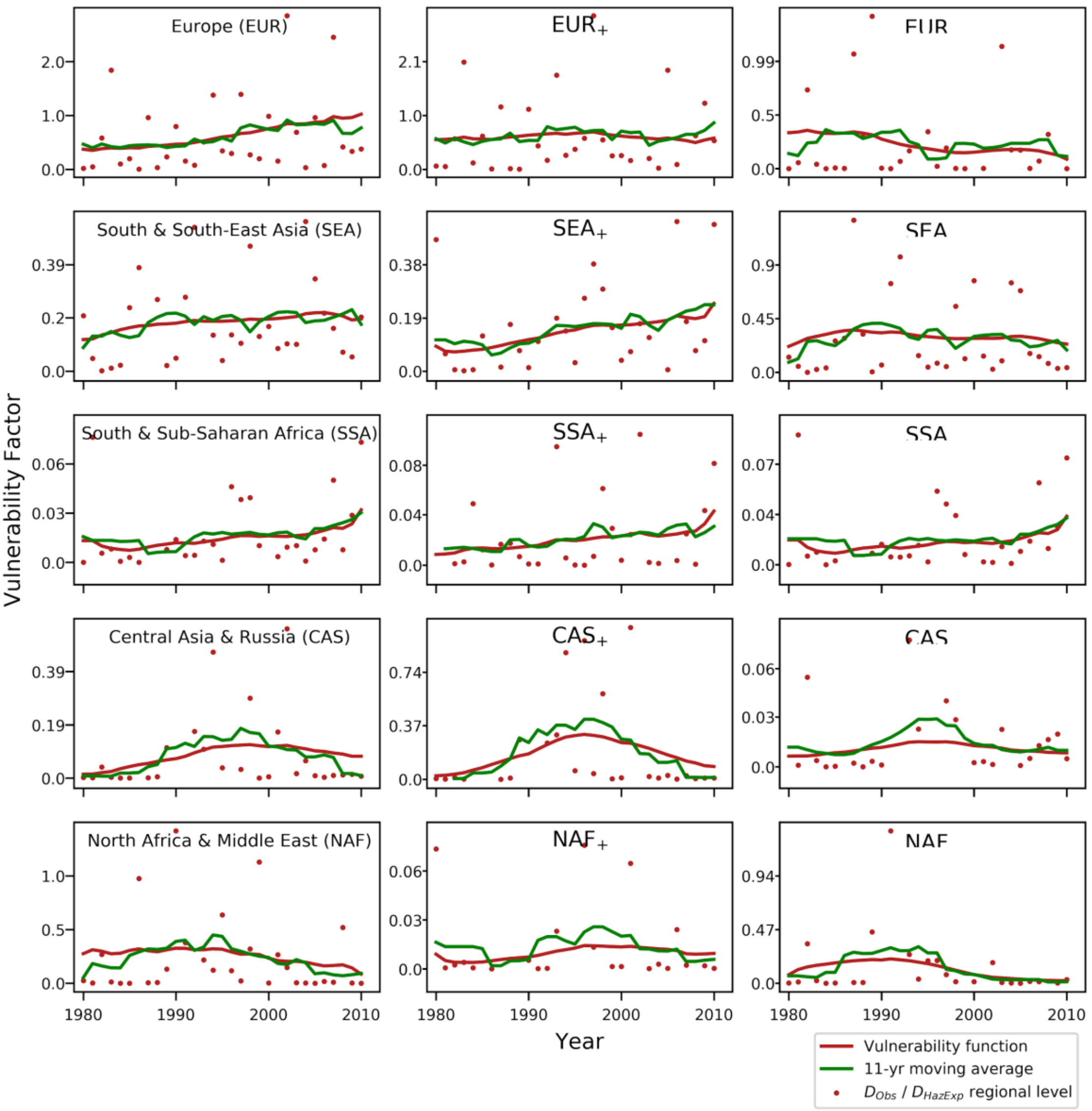

Figure RL3: Scenario for rising damage trends despite declining discharge trend. Extremely low maximum discharges in the end of the time period affect the trend in discharge, but not on the damage trends, as they are below the protection level. Higher maximum discharge in some later years lead to an increasing damage trend.

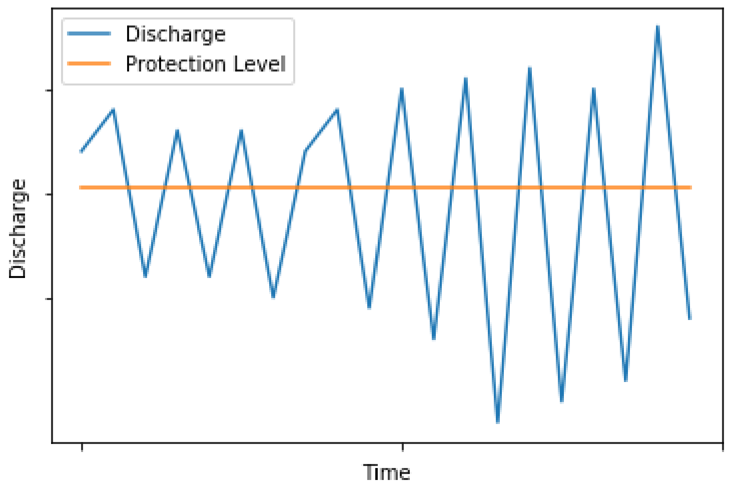

Reviewers' Comments:

Reviewer #1:
Remarks to the Author:
Dear authors,

thank you for revising the manuscript. The changes are considerable, but all in the right direction. The results are now much better aligned to previous research and state of knowledge, the overall uncertainty is lower and more results are statistically significant. All comments were addressed well and I don't really have anything to add; the manuscript is good for publication as it is now.

Best regards
Dominik Paprotny

Reviewer #2:
Remarks to the Author:
Thank you for addressing my concerns and comments given during the first iteration. I thoroughly evaluated the revised manuscript file, your response to the reviewers, and provided tables/figures in the supplementary materials. I appreciate the authors' efforts in improving the initial version of this work from different angles. I can notice that the manuscript has been significantly improved, and remarks made throughout the manuscript are supported with sufficient pieces of evidence. I have no further concerns or comments at this point.
I congratulate the authors for this contribution.

Nasser Najibi

Reviewer #3:
Remarks to the Author:
Dear Author,

The authors responded to my comments and included additional text to explain their results based on the sub-river basin divisions. However, I feel the overall impression that the manuscript is slightly complicated with lots of data and information. For this reason, the manuscript is required to reorganized. My comments and suggestions are as follows:

1. I would suggest that a description of the sensitivity experiment related to Figure 4 be provided in Supplementary Information.
2. The order of the methods should be organized. The order of data, models, and methods of analysis is not consistent, and these should be organized. Also, some paragraphs should be moved to Supplementary Information (e.g., "Limitation and uncertainties").
3. It is also necessary to organize the figures. In particular, as R, R+ and R- in Figure 4 is drawn on a horizontal axis, it is not necessary to classify the data by color. Are the units in Fig. 1a and Fig. 2 correct? The unit of damage in Log 10 in Figure 2 is dimensionless. What is the unit of "a" of "%/a" in Figure 3 and Figure 4?
4. It is necessary to use indices such as "i" and "j" instead of years and countries in Equations 1 and 2.

Best Regards,

Reviewer #1 (Remarks to the Author):

Dear authors,

thank you for revising the manuscript. The changes are considerable, but all in the right direction. The results are now much better aligned to previous research and state of knowledge, the overall uncertainty is lower and more results are statistically significant. All comments were addressed well and I don't really have anything to add; the manuscript is good for publication as it is now.

Best regards
Dominik Paprotny

We thank the reviewer for the acknowledgement of the changes in our work and for the useful previous comments and suggestions, which helped to improve especially the quality of the vulnerability analysis as well as other aspects of our work.

Reviewer #2 (Remarks to the Author):

Thank you for addressing my concerns and comments given during the first iteration. I thoroughly evaluated the revised manuscript file, your response to the reviewers, and provided tables/figures in the supplementary materials. I appreciate the authors' efforts in improving the initial version of this work from different angles. I can notice that the manuscript has been significantly improved, and remarks made throughout the manuscript are supported with sufficient pieces of evidence. I have no further concerns or comments at this point.
I congratulate the authors for this contribution.

Nasser Najibi

We thank the reviewer again for the constructive comments, questions and suggestions, which helped us to improve the quality of our manuscript substantially and for the final positive evaluation of our work.

Reviewer #3 (Remarks to the Author):

Dear Author,

The authors responded to my comments and included additional text to explain their results based on the sub-river basin divisions. However, I feel the overall impression that the manuscript is slightly complicated with lots of data and information. For this reason, the manuscript is required to reorganized. My comments and suggestions are as follows:

We thank the reviewer for pointing out these remaining shortcomings in our manuscript and provide a reorganised version according to the suggestions.

1. I would suggest that a description of the sensitivity experiment related to Figure 4 be provided in Supplementary Information.

We would agree with the reviewer that more background information on attribution definition and the sensitivity analysis will be helpful for the reader. We added a paragraph to the Supplementary Information (SI5).

2. The order of the methods should be organized. The order of data, models, and methods of analysis is not consistent, and these should be organized. Also, some paragraphs should be moved to Supplementary Information (e.g., "Limitation and uncertainties").
We followed the reviewer's suggestion and restructured the Methods. It now contains the following sections and subsections:

Data

- Climate forcings and hydrological data
- Socio-economic data sources
- Climate oscillations and global mean temperature

Flood and damage modeling

- Flood modeling
- Economic damage assessment
- Assessing and accounting for vulnerability

Methods of analysis

- Trend estimation
- Definition of subregions by discharge trends.
- Attributing damages to individual drivers.
- Contribution of global mean temperature and climate oscillations to climate-induced damage trends

Code availability

Data availability

As suggested by the reviewer, we moved several sections including the section on Limitations and uncertainties into the SI.

3. It is also necessary to organize the figures. In particular, as R, R+ and R- in Figure 4 is drawn on a horizontal axis, it is not necessary to classify the data by color. Are the units in Fig. 1a and Fig. 2 correct? The unit of damage in Log 10 in Figure 2 is dimensionless. What is the unit of "a" of "%/a" in Figure 3 and Figure 4?

We agree with the reviewer that the units need to be corrected in some of the Figures. In Figure 1a we show a trend in maximum discharge, so we corrected it to $m^3/s/year$. The y-axis in Figure 2 is dimensionless, but we take the log10 of a damage given in 2005$ and clarified this in the labeling. In Figure 3 and 4 we used 'a' as the unit of year, but changed it

now to ‘year’, as we agree that ‘a’ is not a commonly used unit. Further, we removed the color classification from Figure 4.

4. It is necessary to use indices such as "i" and "j" instead of years and countries in Equations 1 and 2.

We substituted country and year by i and j in the equations.

Reviewers' Comments:

Reviewer #1:
Remarks to the Author:
Dear authors,

the minor changes made after reviewer #3's comments are alright, but I noticed that something is wrong the y axis of Fig. 2, and graph lines are cut by the boxes or spill over. Please correct this, otherwise the paper is fine.

Best regards
Dominik Paprotny

Reviewer #2:
Remarks to the Author:
Thanks.

Reviewer #3:
Remarks to the Author:
Dear Authors,

Thank you for addressing my comments.
By the revision, the manuscript is more clear, easy to understand, and well structured.
I do not have additional comments.

Best regards,

Letter to the reviewers

Reviewer #1 (Remarks to the Author):

Dear authors,

the minor changes made after reviewer #3's comments are alright, but I noticed that something is wrong the y axis of Fig. 2, and graph lines are cut by the boxes or spill over. Please correct this, otherwise the paper is fine.

Best regards
Dominik Paprotny

We thank the reviewer for pointing out this error. We adjusted the y-axis again to represent the time series as good as possible in Fig.2 as well as in Supplementary Fig. 5, 6 and 7.

Reviewer #2 (Remarks to the Author):

Thanks.

We thank the reviewer again for the constructive feedback that allowed us to improve our work.

Reviewer #3 (Remarks to the Author):

Dear Authors,

Thank you for addressing my comments.
By the revision, the manuscript is more clear, easy to understand, and well structured.
I do not have additional comments.

Best regards,

We thank the reviewer for the overall positive evaluation of the manuscript and for the constructive comments that helped us to improve the manuscript substantially.
